# Concise, stereodivergent and highly stereoselective synthesis of *cis-* and *trans*-2-substituted 3-hydroxypiperidines – development of a phosphite-driven cyclodehydration

**DOI:** 10.3762/bjoc.10.35

**Published:** 2014-02-11

**Authors:** Peter H Huy, Julia C Westphal, Ari M P Koskinen

**Affiliations:** 1 Aalto University, School of Chemical Technology, Laboratory of Organic Chemistry, Kemistintie 1, 02015 Espoo, Finland; 2 University of Cologne, Department of Chemistry, Organic Chemistry, Greinstrasse 4, 50939 Cologne, Germany

**Keywords:** amino acids, asymmetric synthesis, cyclodehydration, hydroxypiperidines, natural products, one-pot

## Abstract

A concise (5 to 6 steps), stereodivergent, highly diastereoselective (dr up to >19:1 for both stereoisomers) and scalable synthesis (up to 14 g) of *cis*- and *trans*-2-substituted 3-piperidinols, a core motif in numerous bioactive compounds, is presented. This sequence allowed an efficient synthesis of the NK-1 inhibitor L-733,060 in 8 steps. Additionally, a cyclodehydration-realizing simple triethylphosphite as a substitute for triphenylphosphine is developed. Here the stoichiometric oxidized P(V)-byproduct (triethylphosphate) is easily removed during the work up through saponification overcoming separation difficulties usually associated to triphenylphosphine oxide.

## Introduction

**Figure 1:**
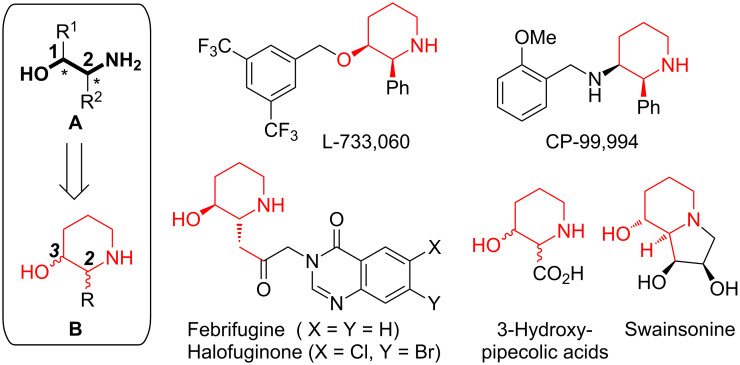
Natural products and other bioactive piperidine derivatives of type **B**.

1,2-Amino alcohols of the type **A** (Figure 1) represent a frequent core motif of many pharmacologically active natural products [1–9], chiral auxiliaries [10–11] and catalysts for asymmetric synthesis [12–14]. Especially the 2-substituted 3-hydroxypiperidine scaffold of the general structure **B** (as one type of an 1,2-amino alcohol) can be found in numerous natural products and other bioactive compounds [1–7]. Selected examples are given in Figure 1: The non-peptidic human neurokinin-1 (NK1) substance P receptor antagonists L-733,060 [15–16] and CP-99,994 [17–19], the natural product febrifugine (antimalarial) [20–21] and antiprotozoal agent halofuginone (commercial trade names Halocur^®^ (lactate salt) and Stenorol^®^ (hydrobromide salt)) [22]. Other relevant examples are 3-hydroxypipecolic acids, which serve as (conformationally restricted) substitutes of proline and serine [23–24] and have been incorporated into diverse bioactive peptidomimetics [25–26], and the iminosugar swainsonine, a new potential chemotherapeutic agent [27–28]. Recently, analogs of halofuginone were discovered as inhibitors of tRNA synthetases [29–31].

The majority of the reported syntheses [2–4,32–35] are elaborate (far more than 10 steps), specific on one of the targets depicted in Figure 1 and therefore on one relative configuration (either *cis*- or *trans*), and have not been proven to be scalable. In our opinion the following examples represent the most efficient synthesis of 3-piperidinols of type **B** in terms of step-economy (<10 steps to establish the core motif **B**): 1. Charette [36] prepared the less bioactive enantiomers of L-733,060 and CP-99,994 through nucleophilic additions to chiral pyridylium salts and subsequent hydrogenation. 2. Cossy and Pardo [37–38] synthesized L-733,060 (formal total synthesis) and two (epimeric) hydroxypipecolic acid through ring expansions of 2-(α-hydroxyalkyl)pyrrolidines deduced to proline. 3. Based on the ring expansion of Cossy and Pardo [37], O´Brien [39] realized the synthesis of L-733,060 (80% ee). The 2-(α-hydroxyalkyl)pyrrolidine precursor was prepared from a protected pyrrolidine through sparteine-mediated enantioselective lithiation and subsequent hydroxyalkylation. 4. Recently, Pansare [40] reported the synthesis of L-733,060, CP-99,994 and a hydroxypipecolic acid through asymmetric organocatalytic vinylogous aldol addition as the key step.

While the syntheses by Charette [38] and Pansare [40] are restricted to piperidinols **B** in *cis*-configuration (dr >19:1 and 8:1, respectively), the sequences of Cossy and Pardo [35] and O´Brien [39] are stereodivergent. Nevertheless, the observed diastereomeric ratios are low (1:1 and 2.3:1 for *trans*-**B**, respectively) at least for one of the epimers. Considering the versatile pharmacological activities of compounds based on the 3-piperidinol scaffold, a step-economic, scalable and stereodivergent synthesis of both *cis-* and *trans-*diastereomers of **B** in good diastereoselectivities is highly desirable.

In the syntheses of potentially new drug candidates scalability is a significant factor to provide sufficient substance amounts for clinical tests [41–42]. Additionally, alternatives in reactions driven by the formation of phosphine oxides from phosphines (e.g. the Appel and Mitsunobu reaction) are highly desired to improve atom economy (reduced waste amounts) and to circumvent difficulties in the separation of these by-products as demonstrated by a number of reviews [43–45]. Numerous protocols have been developed to improve these issues, mostly based on polymer supported or otherwise modified (more complex) phosphines [43–45]. Surprisingly, in this context simple and inexpensive phosphites (P(OR)_3_) have only been applied as phosphine substitutes in one single example: Beal [46] utilized tri-isopropylphosphite in a Mitsunobu condensation of a guanine-derived nucleoside analog with benzylic alcohols providing simplified byproduct separation through improved water solubility (of O=P(OiPr)_3_). In our case we were not able to remove stoichiometric amounts of OP(OEt)_3_ (which is more hydrophilic than OP(OiPr)_3_) through an aqueous work up (without saponification). Moreover, pentavalent P(OEt)_5_ prepared from P(OEt)_3_ with diethylperoxide and ethylbenzenesulfonate, respectively, in an additional step, was reported to effect cyclodehydration of diols to furans and pyrans [47–48] (for recent examples for cyclodehydration protocols see [49–50]). Thereby, the volatile products were separated from O=P(OEt)_3_ through distillation.

After our initial short communication [51] about the step-economic and stereodivergent synthesis of *trans*- and *cis*-2-substituted 3-piperidinols **B**, we want to report the development of this sequence in more detail with a focus on the phosphite-mediated cyclodehydration. Additionally, the synthesis of a side chain functionalized piperidinol derived from methionine and studies towards the preparation of glutamic and aspartic acid derived heterocycles are presented.

**Figure 2:**
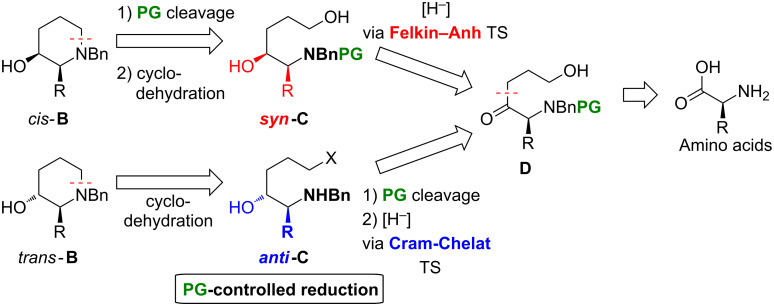
Retrosynthetic analysis of piperidines **B** (X = OH or leaving group, PG = protecting group).

Following the retrosynthetic analysis in Figure 2 the relative configuration of **B** (*cis*/*trans*) should be controlled through targeted protecting group (PG) manipulation: Reduction of the common precursor ketone **D** (derived from commercial available amino acids) should deliver the *syn*-amino alcohol **C** proceeding though a Felkin–Anh transition state [52–53] (due to the sterically demanding –NBnPG function). Further PG cleavage and cyclodehydration would give rise to *cis*-**B**. On the other hand, initial deprotection of **D** (to liberate the Lewis-basic –NHBn moiety) and subsequent reduction passing through a Cram-chelate transition state [54] should deliver the *anti*-amino alcohol **C**.

After subsequent cyclisation *trans*-**B** would result. Noteworthy, this strategy would completely circumvent configurationally labile amino aldehyde intermediates [55–56]. Basically any carbamate or amide protecting group (= PG) orthogonal to the benzyl moiety would be suitable for this strategy. Furthermore, we decided to surrender protection of the OH functions in the synthesis of intermediates **D** and **C** in order to minimize the number of steps of the sequence. Thus far only four examples following a related strategy to establish the *syn*- and *anti*-configuration of 1,2-aminoalcohol motifs have been reported [57–60].

## Results and Discussion

### Synthesis of hydroxyketone intermediates D

**Scheme 1:**
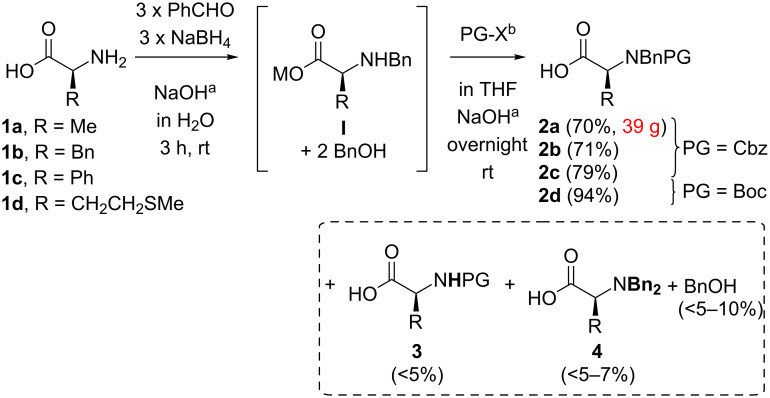
Synthesis of the protected amino acids **2**. (a) KOH for **1b**. b) PG–X = Cbz–Cl (**1a–c**), Boc_2_O (**1d**).

In the first step L-alanine, phenylalanine, phenylglycine and methionine **1a–d** were converted to their *N*-benzyl-*N*-carbamate-protected derivatives **2a–d** (PG = Cbz, Boc) in a practical one pot procedure through combination of Quitt´s reductive benzylation protocol [61] and a Schotten*–*Baumann acylation [62–63] in 70–95% yield (Scheme 1). While we choose a Cbz protecting group for the amino acids **1a–c** due to the mild cleavage conditions (hydrogenolysis), we decided to introduce a Boc group at the *N*-terminus of methionine **1d** to avoid desulfuration (–EtSMe → –Et) in the later deprotection.

In order to suppress the formation of the only carbamate protected amino acid derivatives **3** (remaining as impurity in the isolated products **2**), quantitative benzylation (**1**→**I**) was ensured by successive addition of three portions of benzaldehyde/NaBH_4_ (Quitt´s procedure [61] → two portions) and by maintaining the pH at a value of 10–11. The extractive separation (washing of a basic aqueous solution of the acids **2**) of the two equivalents of BnOH formed during the reductive amination (**1**→**I**) proved to be challenging: Due to the high amphiphilicity of carboxylate salts of **2**, mixtures in water and an organic solvent tend to form three distinct phases separating poorly. Nevertheless, washing of an aqueous solution of the polar lithium salts of **2** (crude **2** in aq. LiOH) with organic solvents of increasing polarity (Et_2_O→EtOAc) allowed to remove BnOH almost completely (<10% of BnOH referred to **2**). Residual benzyl alcohol diminished the yield in the following amidation (**2**→**5**) due to competitive formation of the corresponding benzyl esters. Depending on the NaBH_4_ batch the dibenzyl-protected derivatives **4** formed as side products (<10% referred to **2**). The residual impurities (BnOH and **4**) were separated either through work up (of amide **5a**) or chromatographic purification (of amides **5b–d**) after the following amidation (**2**→**5**).

Importantly, through this procedure not only one isolation step was avoided, but also the overall yield was improved significantly: For the transformation from **1a** to **2a** (**I** isolated as free acid) under (optimized) literature conditions we achieved a yield of 44% over two steps (59% and 74%, respectively) compared to 70% according to our direct conversion from **1a** to **2a**.

**Scheme 2:**
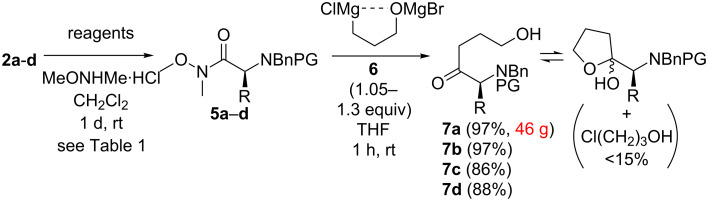
Synthesis of hydroxy ketones **7** (R = Me (**a**), Bn (**b**), Ph (**c**) and EtSMe (**d**); PG = Cbz (**a–c**), Boc (**d**)).

Next the carboxylic acids **2** were transformed to the Weinreb amides **5** with DCC and MeONHMe in 77–91% yield (Scheme 2). While no racemisation occurred with substrates **2a**, **2b** and **2d** (Et_3_N/DMAP or NMM as bases; Table 1, entries 1 and 2), the protected phenylglycine derivative **2c** showed high configurational lability: Under standard conditions (DCC/Et_3_N/0.3 equiv DMAP) the amide **5c** was obtained in a diminished ee of 49% (Table 1, entry 3). HOBt as nucleophilic catalyst proved to be even worse than DMAP, because under otherwise identical conditions the product **5c** was isolated almost as a racemate (5% ee, entry 4). By replacing Et_3_N with the less basic NMM (in the presence of DMAP) the ee increased clearly (49→75%, entry 5). However, without any nucleophilic catalyst the desired amide **5c** was isolated with a very good ee of 95% (entry 6) [64].

**Table 1:** Ees and isolated yields of Weinreb amides **5**.

entry	substrate	R	reagents	ee^a^	yield

1	**2a**	–Me	DCC, NMM	99%	91%^b^
2	**2b**	–Bn	DCC, Et_3_N, DMAP (cat.)^d^	99%	77%^c^

3	**2c**		DCC, Et_3_N_,_ DMAP (cat.)^d^	49%	n.d.
4			DCC, Et_3_N_,_ HOBt (cat.)^d^	5%	n.d.
5		–Ph	DCC, NMM, DMAP (cat.)^d^	75%	n.d.
6			DCC, NMM	95%	81%^b^
7			T3P, pyridine^e^	74%	n.d.

8	**2d**	–EtSMe	DCC, NMM	99%^f^	91%^b^

^a^The ee was determined via HPLC on a chiral stationary phase. ^b^Isolated crude yield, purity >90% according ^1^H NMR. ^c^Isolated yield after chromatography. ^d^cat. = 0.3 equiv. ^e^Solvent EtOAc instead of CH_2_Cl_2_. ^f^The ee was determined to be ≥99% at the later stage the piperidinol *cis*-**11d**. DCC = *N,N*´-dicyclohexylcarbodiimide; NMM = *N*-methylmorpholine; DMAP = *N,N*-dimethylaminopyridine, HOBt = 1-hydroxybenzotriazole; T3P = *n*-propylphosphonic acid anhydride; n.d. = not determined.

The high racemisation sensitivity of **2c** is further underlined by a T3P/pyridine (*n*-propylphosphonic acid anhydride) [65–66] mediated amidation, which has been reported to suppress racemisation [67]. With substrate **2c** the condensation product **5c** was formed in a moderate ee of 74% (Table 1, entry 7). The configurational lability of the phenylglycine derivative was not surprising, as phenylglycine itself is 60 times more prone to racemisation than alanine [68]. The optical purity of amides **5a–c**, hydroxyketone **7c** and piperidins *cis-***11a–d** and *trans*-**11a** (Table 2, Scheme 6 and 7) was determined with HPLC on a chiral stationary phase and comparison with racemic samples (alanine **1a** and phenylglycine **1c** derived substrates) or in analogy to the aforementioned amino acid derivatives (phenylalanine **1b** and methionine **1d** deduced substrates).

In the next step, addition of a slight excess of the 3-chloropropanol-derived Grignard reagent **6** (final concentration 0.25–0.3 mol/L), which was found to be superior in the final concentration to the reported ClMg*n*PrOMgCl derivative (0.1–0.2 mol/L in our hands) [69], to amides **5a–d** resulted in ketones **7a–d** in good to excellent yields (86–97%, Scheme 2). In contrast to the reported procedure (2–3 h of reflux) [69], short reflux times to form **6** (20–30 min) were crucial to avoid decomposition of this dianionic reagent. Furthermore, a slight excess of MeMgBr in the deprotonation step of 3-chloropropanol was found to assist the Grignard formation. Notably, with this strategy we saved additional protection and deprotection steps of the free hydroxy group of **7**. Thereby, only the phenylglycine derivative **7c** displayed a slight decrease of ee (95→92%), the other hydroxy ketones **7a**, **7b** and **7d** did not show any racemisation at all, not even after a longer time of storage. For the latter three the enantiomeric excess was not determined on this step: Further conversion as depicted in Scheme 4 and Table 2 delivered piperidinols **11a**, **11b** and **11d** in ≥99% ee. As no intermediate in the conversion of **7** to **11** was crystallized, the ketones **7a**, **7b** and **7d** must have been enantiopure.

Residual 3-chloropropanol (<15 mol % referred to **7**), which was difficult to separate chromatographically, was removed after the next step either during the work up (diols **9**) or chromatographically (on the sequence leading to **15a**). Interestingly, the ketones **7a–d** existed in an equilibrium with their cyclic hemiacetal tautomers as shown in Scheme 2. The predominant keto form possessed a clear singlet around 205–210 ppm for the quaternary carbonyl carbon and the furan form was indicated by two weak signals at 105–110 ppm for the hemiacetal carbon (two diastereomers) in the ^13^C NMR.

**Scheme 3:**
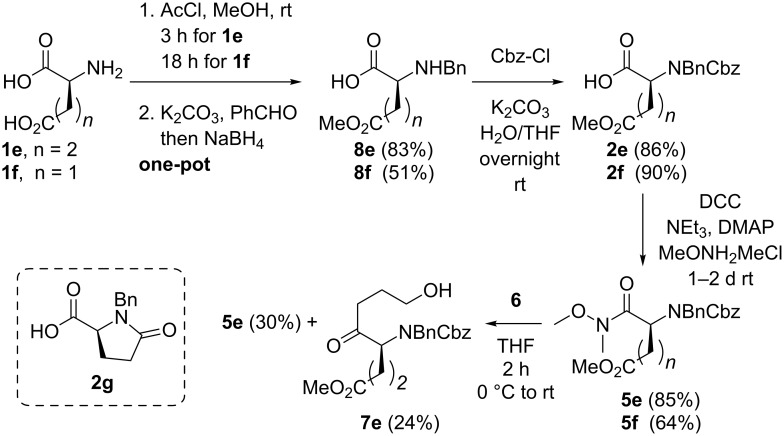
Synthesis of amides **5e** and **5f** and ketone **7e**.

Additionally, a short 3 step route to the functionalized glutamic and aspartic acid derived Weinreb amides **5e** and **5f** was coined as outlined in Scheme 3: At the outset both amino acids (**1e** and **1f**) were subjected to esterification of the sterically less hindered side chain carboxyl function with acetyl chloride in MeOH [70–72]. Neutralization of the reaction mixture with K_2_CO_3_ and reductive benzylation [73] in one-pot then delivered the benzyl amines **8e** and **8f**. While glutamic acid showed selective mono esterification after 3 h at room temperature, the aspartic acid mono ester was obtained in a 5:1 ratio with the diester (not shown) after approximately 18 h of reaction time. Shorter reaction times in the esterification step of **1f** decreased the yield of **8f**.

The amines **8e** and **8f** were converted in a straightforward manner to the carbamates **2e** and **2f** under Schotten*–*Baumann conditions [62–63]. Thus, K_2_CO_3_ (→ pH = 10) as the base prevented saponification of the side chain methyl ester functions (as observed with hydroxide salts). Nevertheless, in the acylation of the glutamic acid derivative **8e** the corresponding pyroglutamic acid derivative **2g** resulting from lactamisation of the ester function was observed as a side product in traces (<5%). Next, amidation of the acids **2e** and **2f** gave the amides **5e** and **5f** in good yields under standard coupling conditions. Unfortunately, the ketone **7e** was obtained in only 24% yield through addition of the Grignard reagent **6** to amide **5e** (along with 30% of reisolated starting material), illustrating the tendency of reagent **6** to attack the side chain ester moiety.

### Synthesis of *syn*-amino alcohols C

**Scheme 4:**
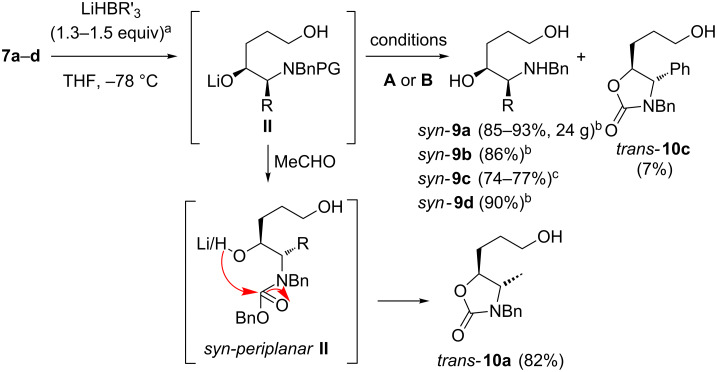
Synthesis of amino alcohols *syn*-**9a–d** and oxazolidinone **10a**. (for **7a–c** conditions **A**: H_2_ (1 atm), Pd/C, HCl, MeOH (THF/MeOH 1:1), rt, 3 h; for **7d** conditions **B**: HCl (aq.; 4 N HCl in THF/H_2_O), rt, 6 h) ^a^R´= *s*Bu for **7a**, **7b** and **7d** (L-Selectride); R´= Et for **7c** (Superhydride). ^b^dr ≥ 19:1. ^c^dr = 3.7–4:1.)

Already the NaBH_4_ reduction (in MeOH at −40/0 °C) of ketones **7a** and **7b** and subsequent hydrogenolysis of the Cbz-group in one-pot delivered the *syn*-amino alcohols **9a** and **9b** in good diastereomeric ratios (around 11:1 *syn*/*anti*, compare Scheme 4). In contrast the NaBH_4_ reduction of ketone **7c** under identical conditions gave diol **9c** without any selectivity (dr = 1.3:1). To our delight, reduction of the ketones **7a** and **7b** with L-Selectride [74] (or N-Selectride) and subsequent hydrogenolysis of the Cbz-group in one-pot delivered the benzyl amines *syn*-**9a** and **9b** in accordance with the Felkin–Anh model in excellent yields and as pure diastereomers as indicated by 400 MHz ^1^H NMR (Scheme 4). Unfortunately, L-Selectride proved to be too unreactive at low temperatures towards the phenylglycine derived ketone **7c**, at higher temperatures mainly decomposition of the starting material was observed. Nevertheless, Superhydride [75–76] reduction and successive Cbz-cleavage gave the desired amino alcohol *syn*-**9c** in useful diastereomeric ratios of 3.7–4:1 *syn*/*anti*. The slightly diminished yield of **9c** (74–77%) compared to **9a** and **9b** (85–93%) is rationalized by formation of the oxazolidinone **10c** as sideproduct. This cyclic carbamate results from the condensation of the alcoholate moiety of **II** with the Cbz-function passing the *syn-periplanar* conformation indicated in Scheme 4. With a bulky phenyl substituent (= R) this conformation is higher populated than with smaller R groups (e.g. Me and Bn) due to increased steric repulsion between the *n-*PrOH and R moiety. This explains the more facile formation of oxazolidinones of type **10** with the phenylglycine derived carbamate **7c**. Indeed, if the excess of Selectride was quenched with acetaldehyde after reduction of **7a**, the oxazolidine **10a** was obtained (under the basic reaction conditions) as the major product.

The outcome of the Cbz-cleavage strongly depended on the activity of the commercial Pd/C batch: While an active catalyst led to a quantitative cleavage after 3 h (all substrates), less reactive batches led to considerably increased reaction times (2 d for **7a**). For **7b** and **7c** the isolated yields of the amines **9** even dropped to 10–20% after 2 d reaction time due to incomplete Cbz-cleavage. However, Pd/C freshly prepared from Pd(OAc)_2_ and activated charcoal according to Felpin [77] delivered consistent results (reaction time < 3 h). Although the Cbz-cleavage beside a benzyl moiety in alcohols as solvent is known (for selected examples of the chemoselective Cbz-cleavage of Bn, Cbz-protected amines see [78–80]), we only achieved high chemoselectivities in the presence of an excess of HCl.

In a similar manner the methionine derived Boc-protected ketone **7d** was converted through L-Selectride reduction and Boc-cleavage induced by aqueous HCl solution in one pot to the *syn*-aminoalcohol **9d** as a single diastereomer according to ^1^H NMR. Importantly for up-scaling, only a slight excess of L-Selectride (1.3 equiv) or Superhydride (1.5 equiv), respectively, was required for the quantitative conversion of **7a–d** to the intermediate **II**. Therefore the deprotonation of the free OH-group of **7** must be significantly slower than the desired reduction of the carbonyl function. Practically, the residual 3-chloropropanol remaining from the prior Grignard addition **5**→**7** step (vide supra) and *sec*-Bu_3_B/BEt_3_ from the reduction were easily separated through washing with Et_2_O of an (HCl) acidic, aqueous phase of the products **9** resulting in the crude diols **9a–d** in a circa 90% purity according ^1^H NMR. Hence, the crude amino alcohols **9** were not further purified before the following cyclodehydration.

### Synthesis of *cis*-piperidinols B

Cyclodehydration of amino alcohol *syn-***9a** to piperidinol *cis-***11a** under Appel conditions (I_2_, PPh_3_) [81–82] surpassed by far Mitsunobu conditions and sulfonation (with MsCl, TsCl) induced cyclisations (see Supporting Information File 1 for more details): Under optimized conditions (1.1 equiv I_2_, Et_3_N in MeCN at −40 °C) the cyclic products **11a–c** were isolated in 68–77% yield and 90–99% ee (Table 2 entry 1, 10, 12). For work up the reaction mixture was simply absorbed on silica gel (ca. 9 fold amount of starting material **9**) through evaporation of the solvent and directly subjected to chromatographic purification.

**Table 2:** Cyclodehydration of amino alcohols **9a–d** to piperidinols **11a–d**.

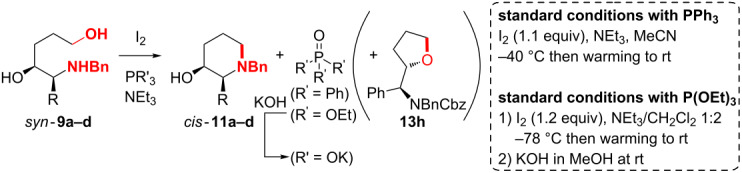					

entry	substrate	R	PR_3_	deviation from standard conditions	yield

1	*syn*-**9a**	–Me	PPh_3_	–	77%^a,b,c^
2			P(OEt)_3_	–	82%^a,b,c^ (14 g)
3			P(OEt)_3_	reflux instead of rt in saponification	52%^a,c^
4			P(OEt)_3_	DIPEA instead of Et_3_N^e^	57%^c,d^
5			P(OEt)_3_	lutidine instead of Et_3_N^e^	50%^c,d^
6			P(OEt)_3_	imidazole instead of Et_3_N^e^	30%^c,d^
7			P(OEt)_3_	Et_3_N/CH_2_Cl_2_ 1:10	79%^c,d^
8			P(OEt)_3_	THF instead of CH_2_Cl_2_^e^	58%^c,d^
9			P(OEt)_3_	−20 °C then warming to 0 °C	41%^a,c^

10	*syn*-**9b**	–Bn	PPh_3_	–	68%^a,b,c^
11			P(OEt)_3_	–	74%^a,b,c^

12	*syn*-**9c**	–Ph	PPh_3_	–	77%^a,e,f^ (2.3 g)
13			P(OEt)_3_	Et_3_N/CH_2_Cl_2_ 1:1.3	68%^a,e,f^
14			P(OEt)_3_	–	49%^a,f^
15			P(OEt)_3_	Et_3_N/CH_2_Cl_2_ 1:3	22%^a,f^

16	*syn*-**9d**	–EtSMe	P(OEt)_3_	–	74%^a,b,c^

^a^Isolated yield after chromatographic purification. ^b^ee ≥ 99%, determined by HPLC on a chiral stationary phase. ^c^dr >19:1 *cis/trans*. ^d^Yield determined with naphthalene as NMR-standard. ^e^Ratio base/solvent 1:10. ^f^ee = 90%; dr = 4.0:1 (entry 12), 5.3:1 (entry 13), 3.7.1 (entry 14), 3.0:1 (entry 15) *cis/trans*. DIPEA = di-isopropylethylamine; lutidine = 2,6-dimethylpyridine. All reactions were run until full conversion of the starting material **9**.

Unfortunately, the stoichiometric byproduct triphenyl phosphine oxide was only separable by chromatography requiring significantly increased amounts of silica gel (the crude product weight usually obtained 300–400% of the theoretical yield after aqueous work up). Attempts to crystallize OPPh_3_ beside the piperidine **11a** or of the fumaric acid salt of **11a** for instance failed, only an oily mixture of **11a** and OPPh_3_ precipitated. Separation of OPPh_3_ through washing of an aqueous solution of hydrochlorides of **11** led to a significant loss of piperidines **11** (especially with **11b** and **11c** bearing lipophilic side chains R).

**Scheme 5:**
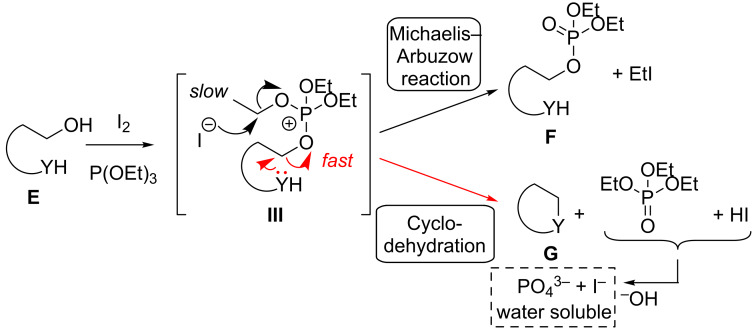
Competition between the Michaelis–Arbuzow process and the desired cyclodehydration of amino alcohols and diols **E** (Y = e.g. O, NBn).

In general, the reaction of alcohols with alkyl phosphites (P(OR)_3_), activated through oxidants such as iodine, have been reported to give the corresponding phosphates in a Michaelis–Arbuzow type reaction [83–85] (Scheme 5). In order to improve atom economy and side product separation, we rationalized that in the phosphonium intermediate **III** (originating from activation of the diol/amino alcohol **E**) the intramolecular substitution by the amino function (delivering the desired heterocycles of type **G**) should be significantly faster than the bimolecular reaction of the iodide ion with intermediate **III** as indicated (resulting in the formation of the corresponding undesired phosphates **F**). The formation of heterocycles **G** passing phosphates **F** through substitution of the phosphate leaving group by the nucleophilic YH moiety would be in principal also a plausible explanation for the formation of **F**, but can most likely be excluded (see Supporting Information File 1).

Indeed, under optimized conditions (1.2 equiv I_2_, P(OEt)_3_, Et_3_N (5–6 equiv)/CH_2_Cl_2_ 1:2; −78 °C then warming to rt) the piperidinols **11a–d** were isolated after saponification (during work up) of triethylphosphate and chromatographic purification in 68–82% yield and high enantiomeric excess (90–99%) (Table 2, entries 2, 11, 13 and 16).

For both cyclodehydration methods (with PPh_3_ and P(OEt)_3_) neat iodine was simply added to a solution of the amino alcohols **9a–d**, the phosphorus reagent and Et_3_N in the reaction solvent at the indicated temperature (−40 °C and −78 °C, respectively) and stirred at this temperature, until I_2_ had dissolved completely (1–2 h with PPh_3_ and 2–5 h with P(OEt)_3_). Then the usually heterogeneous mixture (through precipitated Et_3_NH^+^Cl^−^) was allowed to warm to room temperature to reach full conversion of the starting material **9**. Due to the ammonium salt precipitation and the solid iodine for the large scale preparation of piperidinol **11a** (14 g, Table 2, entry 2) mechanical stirring was preferred. For saponification, saturated KOH solution in MeOH (ca. 4 N) was chosen, because aqueous KOH or NaOH solution would result in biphasic mixtures. A quantitative hydrolysis of the triethylphosphate side product required evaporation of CH_2_Cl_2_ and Et_3_N (and MeOH) after the addition of the methanolic KOH-solution in vacuo, dilution with MeOH, stirring at room temperature for ca. 1 h, and a second concentration under reduced pressure. Most likely, the solubility of KOH in the CH_2_Cl_2_/Et_3_N/MeOH mixture is too low resulting in incomplete hydrolysis of the phosphate without evaporation of the solvent. Saponification at reflux on the other hand (in order to circumvent the solvent evaporations) led to partial decomposition of the product **11a** (isolated yield 52% after several hours of reflux, see Table 2, entry 3). Chromatographic purification of the crude piperidinols **11a–d**, which were already isolated in 85–90% purity (from the phosphite cyclodehydration), was best achieved with halogen free iPrOH/Et_3_N/hexane eluent mixtures (ratio 0.8–3:2–4:100 depending on the polarity of the product). Although the diastereomers of piperidinol **11c** (dr ca. 4:1 *cis*/*trans*) were chromatographically separable, we decided to separate them after protecting group exchange at the stage of the Boc carbamate **16c** (see Scheme 8), because the epimers of the latter one were much easier to isolate.

Interestingly, we observed a high specificity in the base: With pyridine, NMM, DMAP and DBU (1,8-diaza[5.4.0]bicyclo-undec-7-ene), respectively, (instead of Et_3_N) the desired heterocycle **11a** was only formed in traces. DIPEA, lutidine and imidazole delivered **11a** in clearly diminished yields of 30–57% (Table 2, entries 4–6) compared to Et_3_N (79%, entry 7). For comparison we ran the condensation of amino alcohol **9a** to piperidinol **11a** in the same concentration as in Table 2, entries 4–6 and 8 (as opposed to the standard conditions) and determined the yield also through NMR-standard: The yield assigned in that way (79%, entry 7) shows a decent match to the 82% isolated yield obtained under standard conditions (entry 2). Hence, the comparability of Table 2 entry 2 to entries 4–8 is established. In conclusion NMM, pyridine and imidazole might be too weak bases, DBU might be too strong and a base favoring other reaction pathways then the desired cyclodehydration. DIPEA and lutidine could be sterically too hindered and DMAP too nucleophilic inducing side reactions.

Moreover, we observed a strong influence of the ratio of Et_3_N/CH_2_Cl_2_ with substrate **9c**: The phenylglycine-derived hydroxypiperidine **11c** was obtained in 68% yield in Et_3_N/CH_2_Cl_2_ in a 1:1.3 ratio, while a 1:3 solvent mixture gave **11c** in only 22% yield (Table 2, entries 13–15). Indeed, the furan **13h** (see Table 2) resulting from nucleophilic substitution of the primary (activated) OH-function through the secondary hydroxy group of **9c** formed in significant amounts in the cyclisation of amino diol **9c**. This is explained by steric shielding of the amino function (→ decreased nucleophilicity) through the bulky phenyl group in α-position (compared to the less demanding Me and Bn side chains R of substrates **9a** and **9c**). As the R-substituent is in the β-position of the secondary OH-function of diols **9**, this hydroxy moiety is less shielded. For substrate **9c** the yield could not be improved further, because in higher ratios Et_3_N/CH_2_Cl_2_ iodine did not completely dissolve (react) at −78 °C in a reasonable time (<12 h).

This strong influence of the ratio of Et_3_N/CH_2_Cl_2_ can be attributed to the lower solubility of iodine in Et_3_N (which leads to a slower and thus more selective reaction) and general base catalysis: Simultaneous deprotonation (through Et_3_N) in the cyclisation step strongly favours the desired reaction pathway to piperidines **11**. Due to this effect we chose a ratio of Et_3_N/CH_2_Cl_2_ 1:2 as standard conditions for the phosphite mediated cyclodehydration. Typically, 5 to 6 equivalents of Et_3_N resulting in a reasonable concentration of the substrates **11** and **12** (see Table 3) of 0.4–0.5 mol/L (in Et_3_N/CH_2_Cl_2_) were sufficient enough to guarantee magnetically stirring, as the viscosity of the reaction mixture increases through ammonium salt precipitation during the preceding reaction. Noteworthy, Et_3_N is as cheap as common solvents such as THF and CH_2_Cl_2_.

Furthermore, in THF the yield of **11a** decreased to 58% (Table 2, entry 8), whereas in MeCN low conversions were observed most likely through reaction of the solvent with the I–P(OEt)_3_^+^ intermediate and in Et_3_N as the sole solvent the solubility of iodine is too low. Even at room temperature iodine did not dissolve (= react) completely. Also a larger excess of iodine (>1.2 equiv) has a negative effect on the yield, because the secondary hydroxy function probably is activated as well. With the more atom economic P(OMe)_3_ complex product mixtures were obtained (in the case of substrate **9a**). Most likely the differentiation between the Michaelis–Arbuzow and cyclodehydration pathway (see Scheme 5) is declined. Moreover, the reaction temperature has a strong influence: When the condensation of **9a** had been performed at −20 °C for instance, piperidine **11a** was isolated in only 41% yield (Table 2, entry 9). Interestingly, the activation of the primary OH function of substrates **9** in the presence of the secondary OH group proceeded (with PPh_3_ and P(OEt)_3_) in very high chemoselectivity most likely due to steric effects. The corresponding aziridine of **9a** clearly resulting from activation of the secondary hydroxy group and subsequent fast 3-*exo-trig* cyclisation was only obtained as a sideproduct at higher temperatures (I_2_, PPh_3_, imidazole, CH_2_Cl_2_ at 0 °C; see Supporting Information File 1 for more details).

Significantly, 14 g of alanine derivative *cis-***11a** were obtained (cyclodehydration with P(OEt)_3_) in one batch with no purification of the intermediates (**2a**, **5a**, **7a** and *syn-***9a**) at all and an overall yield of 44%, demonstrating the scalability and high practicability of our sequence. The relative configurations of compounds **9** and **11** were proven by NOE spectroscopy of piperidinols *cis-***11a–d**, *trans*-**11a**, *cis-* and *trans-***16c**, L-733,060^.^HCl and of oxazolidinones *trans-***10a** and *trans-***10c** (see Supporting Information File 1 for more details).

### Synthesis of other heterocycles through phosphite-mediated cyclodehydration

We next wanted to demonstrate the generality of the phosphite-mediated cyclodehydration (Table 3). Towards this end, a range of amino alcohols and diols **12a,b** and **12d–h** were converted in usually very good yields (>80%) to pyrrolidines, piperidines and furans **13a,b** and **13d–h**. The established cyclodehydration procedure only had to be slightly modified regarding the work up: As most of the products **13a**,**b** and **13d–h** are volatile, the crude reaction mixture was concentrated under reduced pressure (50 mbar) after quenching with the methanolic KOH solution and kept at this pressure at the rotatory evaporator for ca. 0.5 h. Then the residue was portioned between water and *n*-pentane (high volatility and low solubility of OP(OEt)_3_!) and the organic phase was washed with five portions of water to remove the remaining phosphate (after saponification **13** contained 20–30 mol % of the phosphate). Finally, the heterocycles **13** were isolated without a trace of the phosphoric acid ester (“traceless cyclodehydration”) in >90% purity according to ^1^H NMR.

**Table 3:** Cyclodehydration of amino alcohols and diols **12a–h** to heterocycles **13a–h**.

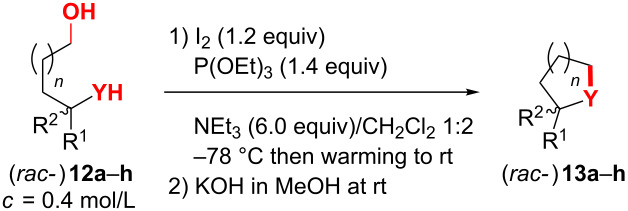							

entry	substrate	YH	R^1^	R^2^	*n*	deviation from standard conditions	yield

1	**12a**	NBn	H	H	1	–	80%^a^
2						washing of **13-HCl** (aq.) with EtOAc instead of saponification	81%^b^

3	**12b**	NBn	H	H	2	washing **13b-HCl** (aq.) with EtOAc instead of saponification	63%^b^
4	**12c**	NBoc	H	H	2	2 d at rt	n. r.

5	*rac-***12d**	OH	Ph	H	1	–	92%^b^
6	*rac-***12e**	OH	*o-*ClPh	H	1	–	85%^b^

7	*rac-***12f**	OH	*p-*BrPh	H	1	–	83%^b^
8						P(OiPr)_3_ instead of P(OEt)_3_	67%^a^
9						P(OPh)_3_ instead of P(OEt)_3_	83%^a^

10	*rac-***12g**	OH	Mes	H	1	–	84%^b^

11	**12h**	OH	Ph	Ph	1	–	82%^b^
12						PPh_3_/MeCN instead of P(OEt)_3_/CH_2_Cl_2_	75%^a^

^a^Yield determined with naphthalene as NMR-standard. ^b^Isolated yields, purity >90% according to crude ^1^H NMR.

We first investigated the amino alcohols **12a–c**: Pyrrolidine **13a** was formed in 80% yield after hydrolysis of the phosphate and in 81% yield after extraction of triethylphosphate with EtOAc (×5) while keeping the product **13a** as the hydrochloride salt in the aqueous phase (Table 3, entries 1 and 2). Surprisingly, with the higher homologue **12b** the piperidine **13b** was obtained in only 63% yield (entry 3). The lower yield compared to the hydroxypiperidines **11a**,**b** and **11d** (≥74%, see Table 2) might be explained by the preference of a conformation of the phosphonium intermediate **III** of **11a**,**b** and **11d** (Scheme 5), which is favourable for the cyclisation. This arrangement might be stabilized through a hydrogen bridge between the NH proton and the O atom of the secondary OH group and a (weak) Thorpe–Ingold effect by the substituent R. The much less nucleophilic Boc-carbamate **12c** was not converted to the desired piperidine **13c** (entry 4). Here only starting material was re-isolated (probably originating from hydrolysis of the corresponding phosphate of alcohol **12c**).

The α-aryl furans **13d–f** were formed in excellent yields (83–92%, Table 3, entries 5–7). Interestingly, the cyclodehydration of *rac-***12f** was also mediated by P(OiPr)_3_ and P(OPh)_3_ in 67% and 83% yield, respectively (entries 8–9). Whereas OP(OiPr)_3_ was hydrolyzed in the work up, OP(OPh)_3_ was not saponified and therefore still remained in the isolated product **13f**. However, considering the atom economy these phosphites do not represent an alternative to P(OEt)_3_. Even the diols *rac*-**12g** and **12h** with a sterically demanding mesityl and two phenyl substituents, respectively, gave cleanly the furans *rac*-**13g** and **13h** in good yields (Table 3, entries 10 and 11). Also in terms of isolated yield the phosphite-mediated cyclodehydration of substrate **12h** was superior (82%, Table 3, entry 11) to the phosphine driven conditions (75%, entry 12).

### Synthesis of a *trans*-piperidinol B

**Scheme 6:**
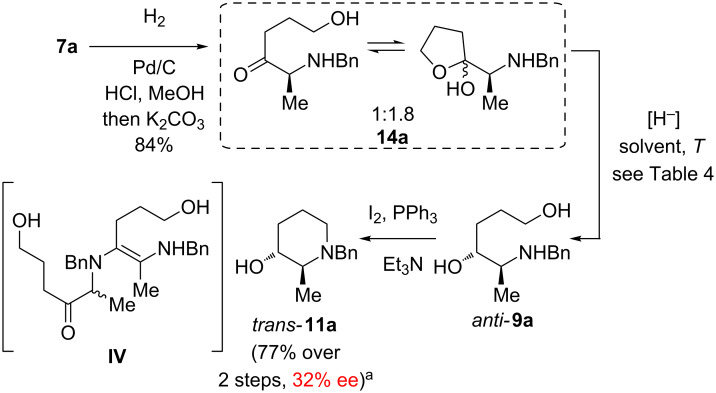
Initial synthesis of the *trans*-piperidinol **11a** in diminished enantiopurity. ^a^The amino alcohol **9a** obtained through L-Selectride reduction according to entry 6 in Table 4 (dr = 25:1) was subjected to cyclodehydration.

We initially investigated the diastereoselectivity of the reduction of the secondary benzylamino ketone **14a**, which was synthesized from hydroxyketone **7a** through Cbz-cleavage and basic work up (Scheme 6 and Table 4). According to ^1^H NMR the hemiacetal of **14a** (ca. 1:1 ratio of its epimers) forms an equilibrium with its ketone tautomer in a 1.8:1 ratio. In contrast to hydroxyketones **7a–d** the furan tautomer of **14a** is thermodynamically more stable than the keto form. This might be rationalized by a lower steric strain in the heterocyclic form due to the smaller NHBn side chain (compared to NBnCbz in **7a–d**).

**Table 4:** Diastereoselectivity in the reduction of ketone **14a**.

entry	[H–]	solvent	*T* [°C]	dr (*anti*/*syn*)

1	NaBH_4_	MeOH	0	1:1^a^
2	NaBH_4_, HCl	MeOH	0	2.6:1^a^
3	NaBH_4_, CeCl_3_	MeOH	−78	2.9:1^a^
4	DIBALH	divers^b^	−78	1.4–2.6:1^c^
5	L-Selectride	THF	−78	6:1^d^
6	L-Selectride	CH_2_Cl_2_/THF^d^	−78	25:1^e^
7	N-Selectride	THF	−78	>50:1^e^

^a^The amino alcohol **9a** was isolated in 87% (entry 1), 85% (entry 2), 93% (entry 3) yield and >90% purity according to ^1^H NMR. ^b^CH_2_Cl_2_, *n-*Hex/THF or *n-*Hex/CH_2_Cl_2_. ^c^40–60% conversion were achieved, starting material was not separated. ^d^A solution of the starting material **14a** in CH_2_Cl_2_ was treated with a commercial solution of L-Selectride in THF. ^e^60–80% conversion were achieved, starting material was not separated.

Disappointingly, in the reduction with NaBH_4_ no selectivity was observed at all (dr = 1:1 *anti*/*syn*, Table 4, entry 1). However, in the presence of one equivalent of HCl the diol **9a** was isolated in a moderate dr of 2.6:1 (entry 2), which demonstrated the formation of a Cram chelate transition state stabilized through a hydrogen bond of the hydrochloride of **14a**. Under Luche conditions (NaBH_4_, CeCl_3_) [86] a similar result was attained (dr = 2.9:1, Table 4, entry 3).

DIBALH delivered amino alcohol **9a** (various solvents tested) only in poor selectivities (dr up 2.6:1, Table 4, entry 4), which is in harsh contrast to previously reported reductions of related *para*-methoxybenzylamino and benzylamino ketones with DIBALH giving rise of the best diastereoselectivities [59–60]). Albeit up to four equivalents of DIBALH were utilized, only 40–60% conversion of **14a** was reached. Here the complexation by –AliBu_2_ after the initial deprotonation of the OH and NH function of **14a** might shield the carbonyl group and encumber reduction.

However, L-Selectride reduction resulted in better stereoselectivities with dr = 6:1 *anti*/*syn* (Table 4, entry 5). If the starting material **14a** was dissolved in the less polar CH_2_Cl_2_ (rather than THF), the dr further increased to >19:1 (entry 6). Moreover, N-Selectride provided the product **9a** almost as a pure diastereomer (entry 7, solvent THF), only a very small trace of the *syn*-diastereomer was visible in the ^1^H NMR (400 MHz). Nevertheless, even with 4 equivalents of Selectride conversions of only up to 80% were observed. Unfortunately, subsequent Appel reaction of the diol *anti*-**9a** (dr = 25:1), synthesized through L-Selectride reduction in THF/CH_2_Cl_2_ (Table 4, entry 6), gave piperidinol **11a** in a significantly diminished ee of 32% (determined via HPLC on a chiral stationary phase and comparison with a racemic sample). This racemisation might be rationalized by an intermolecular enamine formation of the secondary amino ketone **14b** as depicted in the intermediate **IV**, Scheme 6.

**Scheme 7:**
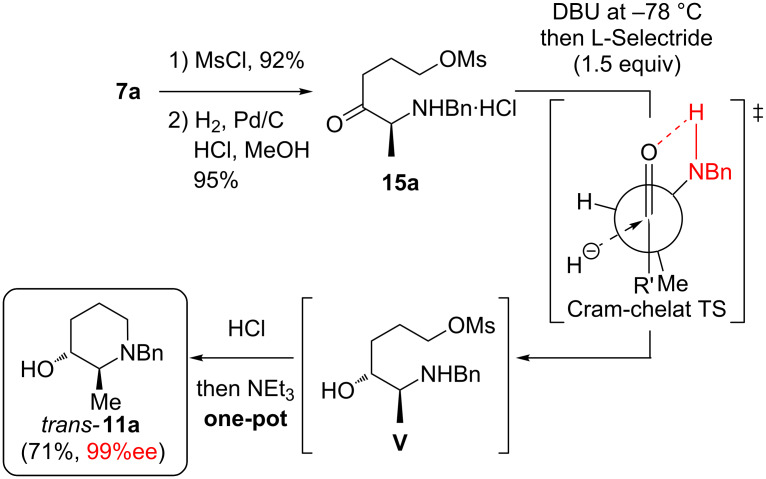
Synthesis of *trans*-piperidinol **11a** in excellent ee.

At this point we realized that a hydrochloride of the secondary amino ketone **14a** or a derivative should be unable to racemise through autocatalytic enamine formation (due to the protonation of the amino function). However, the hydrochloride of amine **14a** was poorly soluble in organic solvents, so that its isolation proved to be difficult. Straightforward, mesylation of hydroxyketone **7a** and subsequent Cbz-cleavage (H_2_, Pd/C) in the presence of HCl delivered the hydrochloride salt **15a**, which was easily isolated through filtration and solvent evaporation (Scheme 7). To our delight, subsequent liberation of the free amine through DBU at low temperature, immediate L-Selectride reduction (giving intermediate **V**), HCl quenching and Et_3_N-induced cyclisation afforded the piperidine *trans*-**11a** in an excellent ee (≥99%) and as a single diastereomer according to crude ^1^H NMR. Although the reduction is performed in the presence of a secondary amino function bearing an N–H-proton and one equivalent of DBU-H^+^, only 1.5 equivalents of L-Selectride were required for a quantitative conversion. Thus we assume the Cram chelate transition state is formed through an amine N–H proton rather than an amide N–Li lithium cation as shown in Scheme 7 (which would result from deprotonation of the amino group by L-Selectride and would thus consume at least 2 equivalents of the reducing agent).

### Synthesis of L-733,060

**Scheme 8:**
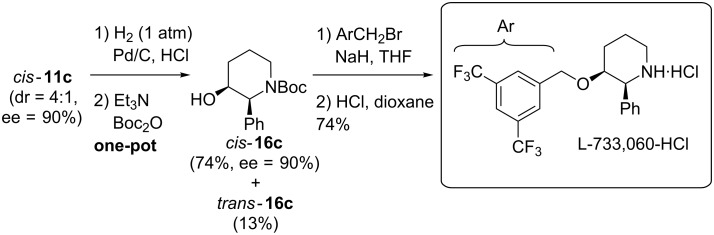
Synthesis of L-733,060·HCl.

In order to probe the practicability of our sequence we synthesized L-733,060 as shown in Scheme 8. After cleavage of the Bn-group under 1 atm of hydrogen and subsequent Boc-protection in one pot, the diastereomers *cis*- and *trans*-**16c** were easily separated by flash chromatography. Thereby, we found it advantageous to perform the hydrogenolysis in the presence of HCl to protonate the released amine and then induce Boc protection after neutralisation of the acid by Et_3_N rather than to run the hydrogenolysis in the presence of Boc_2_O. As already observed in the reduction/Cbz-cleavage **7**→**9** (Scheme 4) the quality of the Pd/C batch had a high influence on the hydrogenolysis: No Bn cleavage was observed with Pd/C charges of a low activity, more catalytically active batches and freshly prepared Pd/C [77] led to quantitative conversion within 1–2 d (1 atm H_2_). The resulting alcohol *cis*-**16c** was subjected to Williamson etherification and subsequently the Boc-group was cleaved under acidic conditions (HCl in dioxane). We decided to isolate L-733,060 as its hydrochloride salt, because it is a non-hygroscopic solid (rather than an oil) and can be easily extracted with organic solvents (e.g. EtOAc) from an aqueous phase. With 8 steps, our sequence represents one of the shortest syntheses reported to date [38–40]. Additionally, with the carbamate *cis*-**16c** (synthesized in 6 rather than 8 steps) we also achieved a formal total synthesis of CP-99,994 [87].

Although the phenylalanine and phenylglycine-derived piperidinols **11b** and **11c** bear “unfunctionalized” side chains, phenyl groups represent masked carboxylic acid functions. For instance, the enantiomers of piperidine *cis-***11c** and its *N*-deprotected derivative were converted to (2*S,*3*R*)-3-hydroxypipecolic acid through protecting group manipulation and oxidative cleavage of the phenyl group with RuCl_3_ and NaIO_4_ [38,40].

## Conclusion

Herein we presented a highly stereodivergent (dr up to 19:1), scalable and practical (up to 14 g of *cis-***11a** without any purification of intermediates) synthesis of *cis*- and *trans*-configured 3-piperidinols **11**, which represent a key structural motive in various natural products and other bioactive target compounds. Moreover, a high step-economy (5–6 steps) was guaranteed by several novel one-pot procedures (**1**→**2**, **7**→*syn-***9**, **15a**→*trans*-**11a**) and surrendering any protection of OH functions. To probe the efficiency of this sequence piperidinol **11c** was converted to the NK-1 inhibitor L-733,060 in three further steps. Additionally, a unique cyclodehydration procedure replacing PPh_3_ through P(OEt)_3_ to improve atom economy (166 compared to 262 g/mol) and to allow separation of the oxidized side product (OP(OEt)_3_) by saponification (no similar literature precedents known) was implemented. Ongoing research is focusing on the transformation of the methionine-derived piperidinol **11d** to other pharmacologically relevant targets on a gram scale.

## Supporting Information

File 1 Experimental and characterisation data.
