# Supplementary material for: Concise, stereodivergent and highly stereoselective synthesis of cis- and trans-2-substituted 3-hydroxypiperidines – development of a phosphite-driven cyclodehydration
Source: Beilstein J Org Chem. 2014 Feb 11;10:369–83. doi: 10.3762/bjoc.10.35 (PMC3943667; doi:10.3762/bjoc.10.35)
Supplement: File 1 — Experimental and characterisation data. [file Beilstein_J_Org_Chem-10-369-s001.pdf]

Supporting Information

for

Concise, stereodivergent and highly stereoselective synthesis of cis- and trans-2-substituted 3-hydroxypiperidines – development of a phosphite-driven cyclodehydration

Peter H. Huy*[1§], Julia C. Westphal[2] and Ari M. P. Koskinen*[1]

Address: [1]Aalto University, School of Chemical Technology, Laboratory of Organic Chemistry, Kemistintie 1, 02015 Espoo, Finland and [2]University of Cologne, Department of Chemistry, Organic Chemistry, Greinstrasse 4, 50939 Cologne, Germany

Email: Ari. M. P. Koskinen - ari.koskinen@aalto.fi; Peter. H. Huy - peter.huy@uni-saarland.de

*Corresponding author

§Current Address: Saarland University, Institute of Organic Chemistry, Mailbox 151150, 66041 Saarbrücken, Germany

Experimental and characterisation data

Index

1 Additional information S3

1.1 Synthesis of *cis*-piperidinols **11** (Table 2) S3

1.2 Synthesis of further substrates **12** for the I_2/P(OEt)$_3$ cyclodehydration and mechanistic discussion (Table 3) S5

1.3 Determination of the relative configuration of piperidinols **11** S6

2 General experimental conditions 10

3 Experimental procedures and analytical data 11

3.1 Synthesis of *N*-Benzyl-*N*-carbamate-protected amino acids **2a–d** (Scheme 1) 11

3.2 Synthesis of Weinreb amides **5a–d** (Scheme 2 and Table 1) 15

3.3 Synthesis of hydroxyketones **7a–d** (Scheme 2) 18

3.4 Synthesis of glutamic and aspartic acid derivatives **7e** and **5f** (Scheme 3) 21

3.5 Synthesis of *syn*-amino alcohols **9a–d** and oxazolidinone **10a** (Scheme 4) 37

3.6 Synthesis of piperidinols *cis*-**11a–d** (Table 2) 44

3.7 Synthesis of heterocycles **13a–h** and cyclisation precursors **12a–h** (Table 2) 56

3.8 Initial synthesis of *trans*-piperidinol **11a** (Scheme 6) 65

3.9 Improved synthesis of *trans*-piperidinol **11a** (Scheme 7) 71

3.10 Synthesis of *L-733,060* (Scheme 8) 71

1 Additional information

1.1 Synthesis of *cis*-piperidinols 11 (Table 2)

Initially, we wanted to realize the cyclodehydration of amino alcohol **9a** to piperidinol **11a** through selective sulfonation of the primary (sterically less hindered) hydroxy group of **9a**, because water soluble and hence through an aqueous work up separable sulfonate salts are formed as stoichiometric byproducts (Scheme S1). However, tosylation in the presence of DMAP at room temperature delivered the tosylamide **17** as the major product, the desired piperidine **11a** was only formed in traces (ca. 10% estimated from the crude ^{1}H NMR, see Scheme S1 (a)).

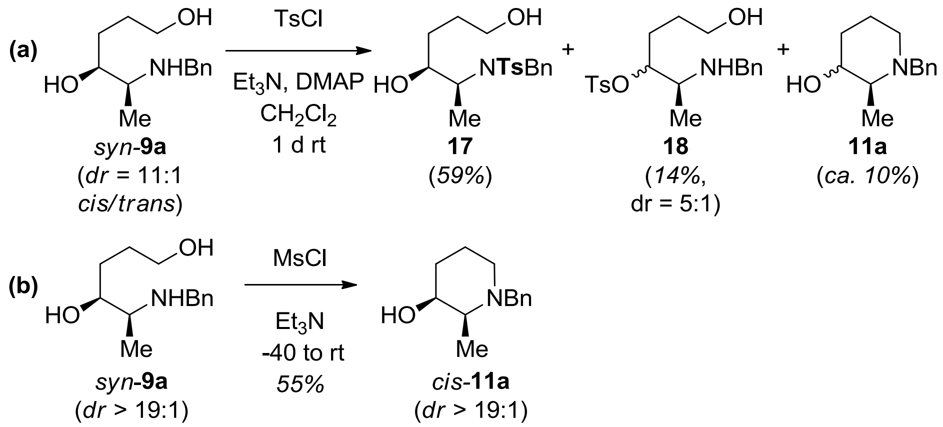

Scheme S1: Cyclodehydration of amino alcohol **9a** to piperidine **11a** through sulfonation.

Mesylation at lower temperatures in the absence of a nucleophilic catalyst provided the *N*-heterocycle **11a** in a moderate yield of 55%, which could not be optimized further (Scheme S1 (b)). Next, we developed an alternative cyclodehydration protocol based on the pre-activation of the primary OH substituent as illustrated in Scheme S2: After mesylation of alcohol **7a** the resulting ketone **19a** (an intermediate in Scheme 7) was reduced with $NaBH_4$ in MeOH at –40 °C. Subsequently, quenching with conc. HCl-solution (aq.), hydrogenolysis (Pd/C, H_2) of the Cbz-group of carbamate **VI** and Et_3N induced cyclisations delivered the *cis*-piperidinol **11a**. Thus in combining three transformations (reduction/Cbz-cleavage/cyclisation) in one pot, we obtained the piperidinol *cis*-**11a** in a yield of 65% and a dr of 10:1 (*cis/trans*) with water soluble methyl sulfonate as stoichiometric by product.

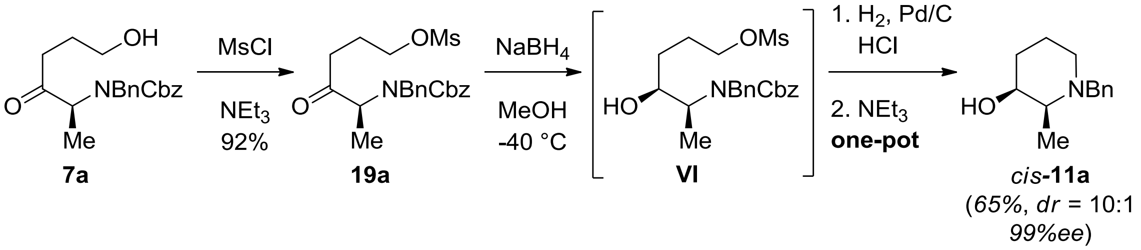

Scheme S2: Synthesis of the *cis*-piperidinol **11a** from the hydroxy precursor **7a** through mesylation, reduction, Cbz-cleavage and cyclisation.

Unfortunately, we were neither able to improve the yield nor the diastereoselectivity in the reduction. In contrast to the $NaBH_4$ reduction, L-Selectride reduction of ketone **19a** and quenching with HOAc/aqueous HCl-solutions resulted approximately in 1:1 mixtures of the desired carbamate **VI** and the oxazolidinone **10a** presumably through the more basic conditions (see Scheme 4). In the following we succeeded in establishing the phosphite-driven cyclodehydration as shown in Table 2 being superior in both yield and diastereoselectivity (through the foregoing L-Selectride reduction in Scheme 4). Furthermore, the chemoselective activation of the primary OH-function of diols **9** proceeded in high chemoselectivity with I_2/PPh_3 and $I_2/P(OEt)_3$: Only at high temperatures we observed formation of the aziridine **20a** in small amounts (Scheme S3).

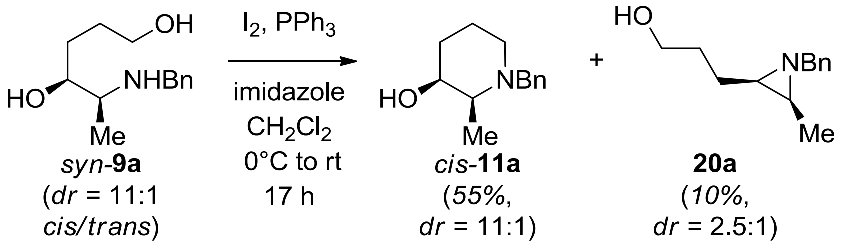

Scheme S3: Formation of the aziridine **20a** as a side product in the cyclodehydration.

1.2 Synthesis of further substrates 12 for the $I_2/P(OEt)_3$ cyclodehydration and mechanistic discussion (Table 3)

The substrates for the cyclisation study (see Table 3) were synthesized either by reductive amination of the commercial amino alcohols **21a** and **21b**, Boc-protection of amino alcohol **21b** (Scheme S4 (a)) or by addition of the Grignard reagent **6** to the carbonyl compounds **21d–h** (Scheme S4 (b)):

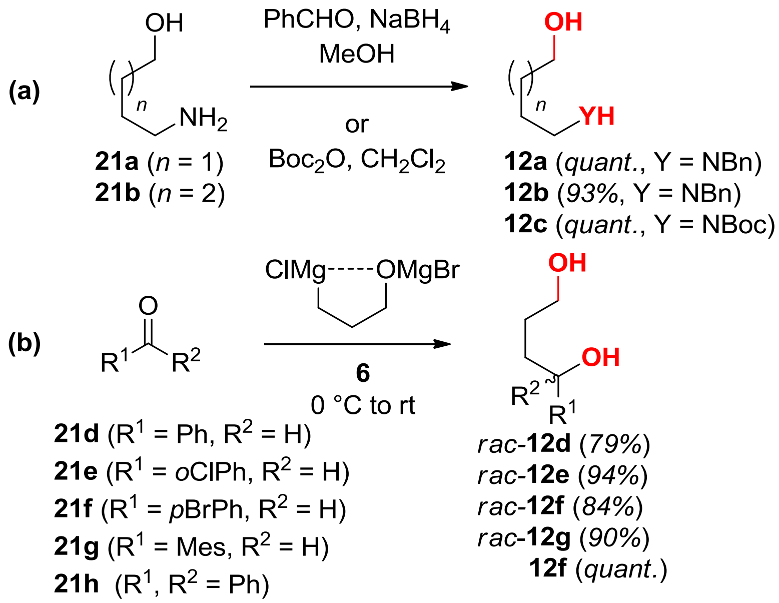

Scheme S4: Synthesis of the amino alcohols and diols **12a–f**.

Considering the known mechanism of the Appel-reaction [1], most likely the cyclisation of amino alcohols and diols **E** to the heterocycles **G** is commenced by the formation of the phosphonium intermediates **IIIa** and **IIIb** as depicted in Scheme S5. In the case of PPh_3 most likely the phosphonium salt **IIIa** directly gives the product **G** in an *intramolecular* S_N2 substitution, as the plausible iodide **VII** would be formed in a slower *intermolecular* substitution. Additionally, $O{=}PPh_3$ and I^- are both very good leaving groups.

In the cyclodehydration involving $P(OEt)_3$ the formation of the iodide **VII** presumably plays no role, too: Statistically the nucleophilic attack of iodide ion at one of the three Et groups of intermediate **IIIb** (giving phosphate **F**) is much more likely than at the single primary carbon of the diol/amino alcohol skeleton. Indeed, the phosphate **F** could also be a possible intermediate leading to the desired heterocyclic products **G** through a nucleophilic displacement of phosphate moiety. However, owing to the fact that the cyclisation mediated by $P(OEt)_3$ takes place at a much lower temperature (quantitative conversion at –78 °C for substrates *syn*-**9a** and **9c**) as with PPh_3 clearly excludes this pathway. As the phosphate group of **F** is a much worse leaving group than $O{=}PPh_3$ and iodide, the cyclisation should only proceed at higher temperatures. If the phosphate **F** does not react further to the product **G**, we can

[1] R. Appel, R. Kleinstück, *Chem. Ber.* **1974**, *107*, 5-12.

also exclude its formation in reasonable amounts: After saponification it would result in the starting material **E**, but after the cyclodehydration (**9→11** in Table 2 and **12→13** in Table 3 except **13c**), we only reisolated trace amounts of the starting materials **9**/**12** (if at all).

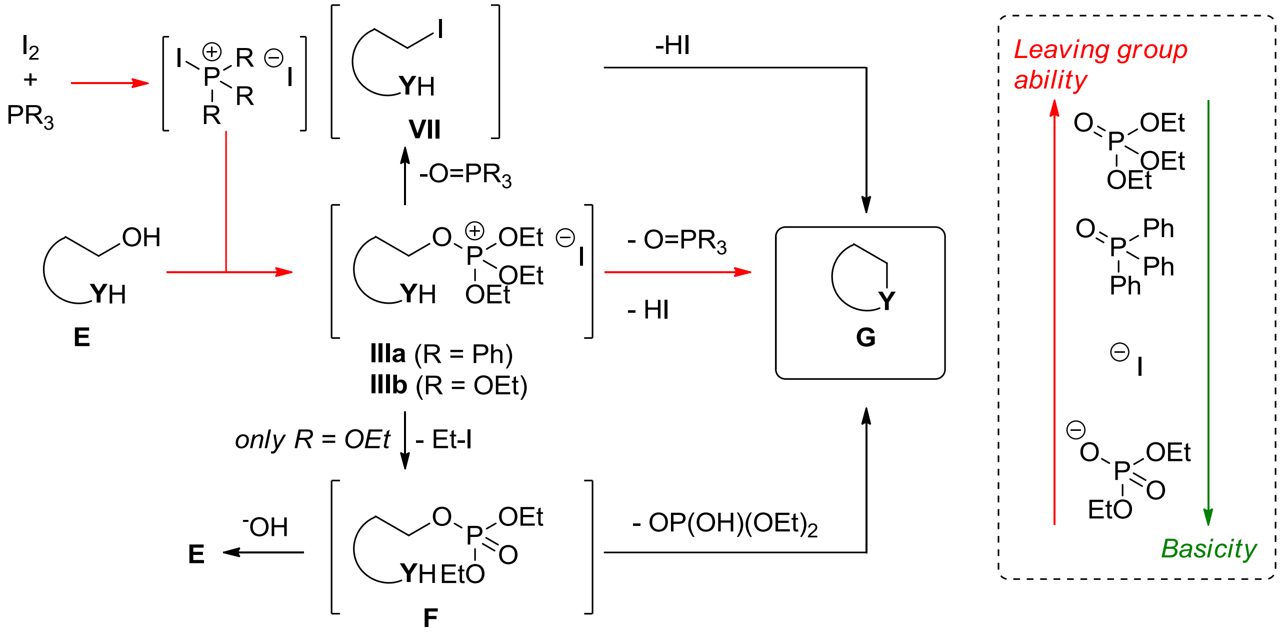

Scheme S5: Possible mechanistic pathways for the I_2/PPh_3 and I_2/$P(OEt)_3$ mediate cyclodehydrations.

1.3 Determination of the relative configuration of piperidinols 11

The relative *cis*-configuration of the piperidines *cis*-**11a–d** was validated through NOE-NMR-spectroscopy (Figure S1): While a clear NOE was observed between H-2 and H-3 (1.2–3.5%), no (*cis*-**11b**) or only a week NOE occurred between H-3 and the thus *trans*-oriented Me- (**11a**) and CH/CH_2-protons of the neighboring Ph and -EtSMe substituent (**11c/d**), respectively (0.5–0.9%). Furthermore, in derivative **11b** the *cis*-configuration is also shown by a clear NOE (1.8–2.0%) between H-2 and H-3, respectively, and the same proton of the CH_2-group on the opposite site of the piperidine ring system (H-4_b). On the other hand in the *trans*-piperidinol **11a** a strong NOE (1.2%) between H-3 and the Me-group was visible while NOE occurred with H-2.

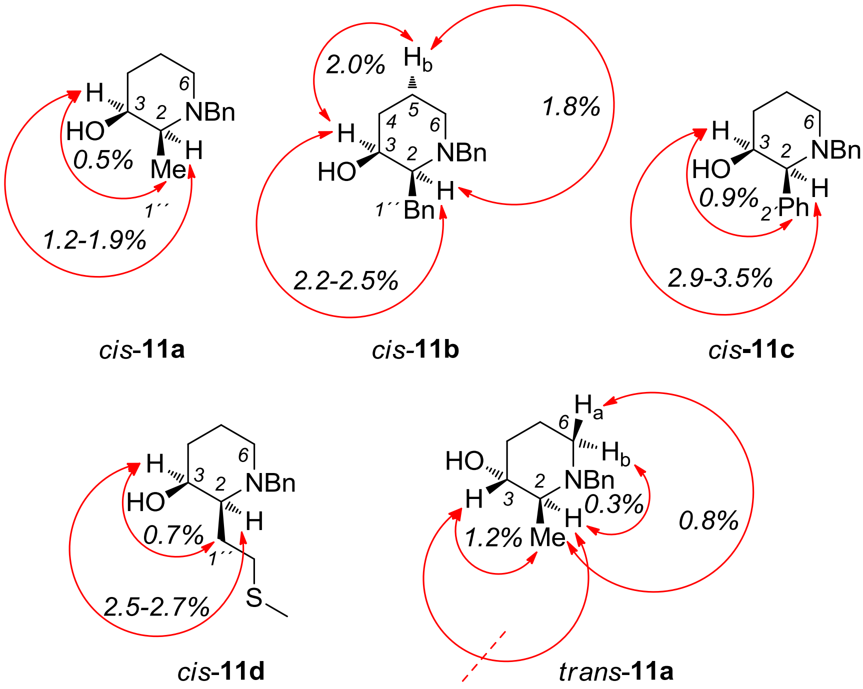

Figure S1: Proof of the relative configuration of **11a–d** through NOE-NMR spectroscopy.

The relative *trans*-configuration of the oxazolidinone **10a** was also established by NOE-spectroscopy (Figure S2): The NOE between H-3 and the methyl group (2.7%) was significantly stronger than for H-2 and H-3 (1.0%). As the reduction conditions in the synthesis of **10a** are identical with those of amino alcohol *syn*-**9a** (the precursor of *cis*-**11a**, see Table 2), the hydroxypiperidines **11a** must be *cis*-configured. Additionally, the oxazolidinone **10c** was isolated as a side product in the reduction of carbamate **10c** (see Scheme 4). Again a relative *trans*-configuration was clearly proven by strong NOE effects between the protons of the phenyl moiety and H-3 (1.6%) and H-2 and the first CH_2 group of the hydroxypropyl side chain (H-4) of 3.4%.

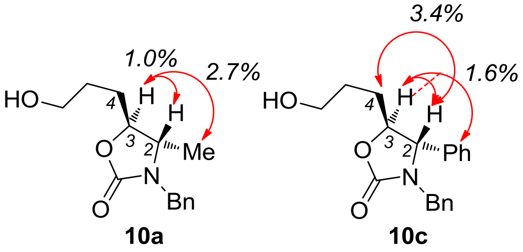

Figure S2: Observed NOE effects demonstrating the *trans*-configuration of oxazolidinones **10a** and **10c**.

The relative configuration of the major and the minor Boc-protected piperidinol epimers **16a** (prepared as given in Scheme 8) derived from the piperidinol **11c** were elucidated by NOE-spectroscopy to be *cis* and *trans*, respectively (Figure S3). In the major epimer of **16a** a stronger NOE (5.3–5.9% compared to 3.3–4.4%) between H-2 and H-3 proofs the *cis*-orientation. Additionally, H-3 shows in the minor epimer of **12a** a clear NOE (7.2%) with the protons of the phenyl moiety, while in the other diastereomer only a very week NOE occurred between the same protons (0.6%) demonstrating a *trans*-configuration.

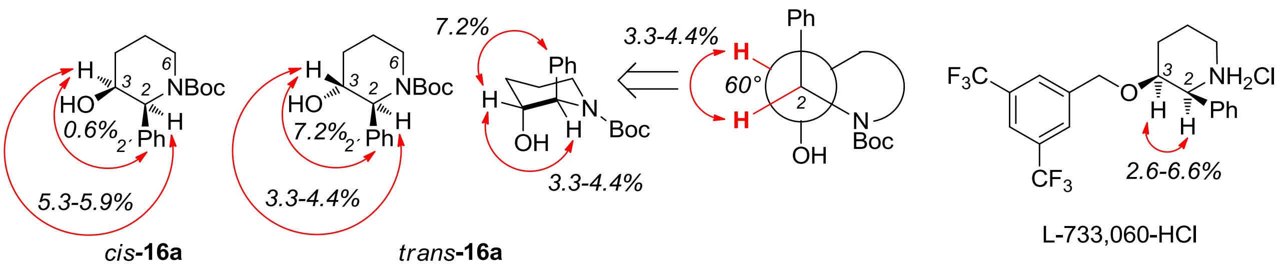

Figure S3: Relative configuration of piperidinols **16a** and L-733,060-HCl.

The surprisingly strong NOE between H-2 and H-3 of *trans*-**16a** (but still weaker than in the *cis*-isomer) can be rationalized by the chair conformer depicted in Figure S3: The phenyl substituent might be forced in an axial position through the sterically demanding Boc-group on the neighboring nitrogen atom. Thus both hydrogen atoms, H-2 and H-3, would end up in equatorial orientations providing a very small dihedral angle (H-2–C-2–C-3–H-3) of approximately 60°). Also in *L-733,060-HCl* a clear evidence for a *cis*-orientation is delivered by a strong NOE between H-2 and H-3 (2.6–6.6%) as depicted in Figure S3. Figure S4 the ^{1}H NMR-spectra and the NOE-spectra with irradiation on the multiplets of H-3 and H-2 (in the order top to bottom) of piperidines *cis*-**11d** are shown. The NOE- and NOESY-spectra of compounds *cis*-**11a-c**, *trans*-**10a** and **10c**, *trans*-**11a**, *cis*- and *trans*-**16c** and L-733,060-HCl can be found in the Supporting Information of our initial communication [2], which are available free of charge under “http://pubs.acs.org/doi/suppl/10.1021/ol4026588”.

2. Huy, P. H.; Koskinen, A. M. P. *Org. Lett.* **2013**, *15*, 5178-5181. DOI 10.1021/ol4026588

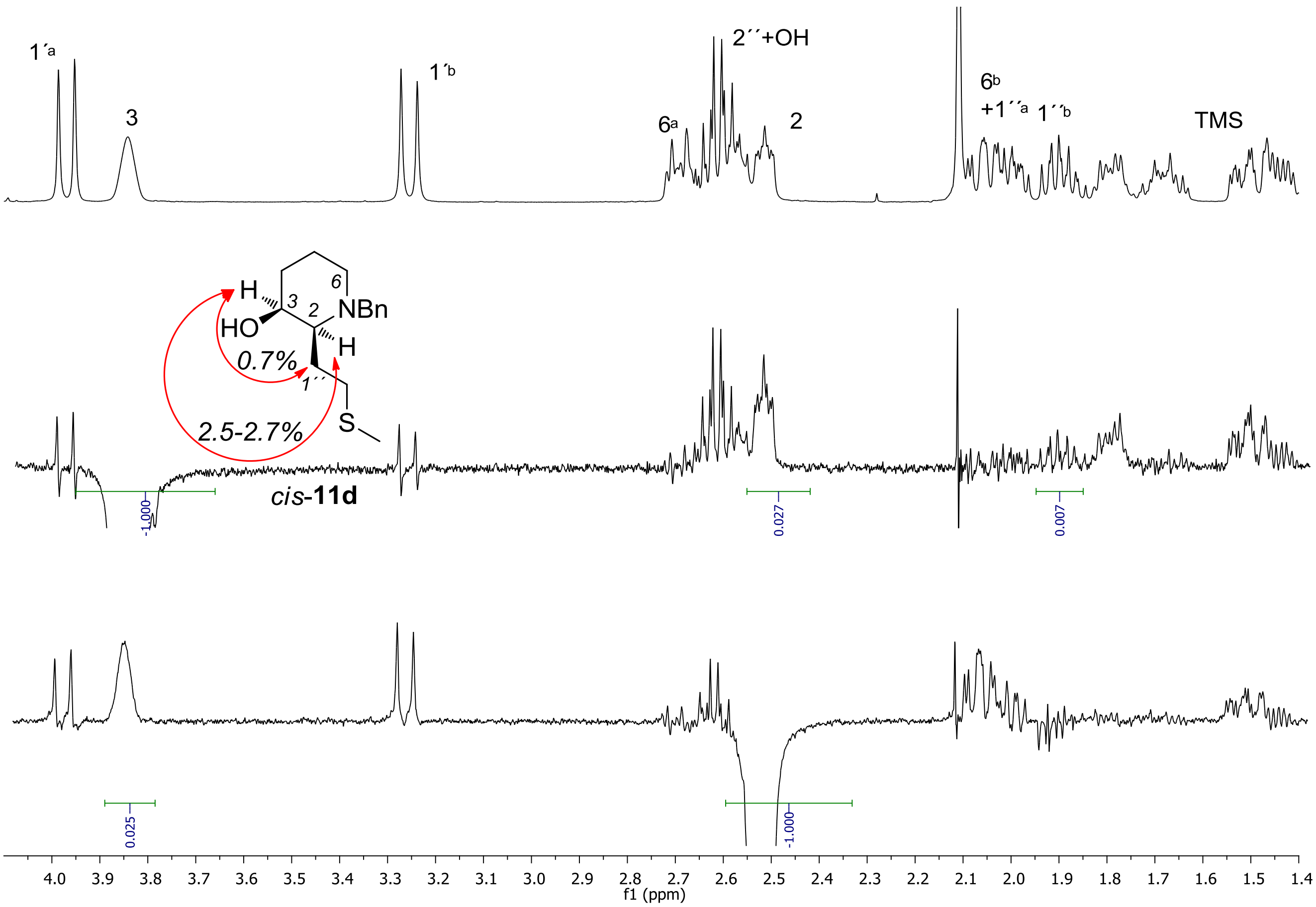

Figure S4: ^{1}H NMR-spectra and the NOE-spectra with irradiation on the multiplets of H-3 and H-2 (in the order top to bottom) of piperidine *cis*-**11d**.

2 General experimental conditions

Unless otherwise stated all ^1H and ^{13}C NMR spectra were recorded at room temp. on a Bruker Avance 400 spectrometer. Chemical shifts (δ) are reported in parts per million (ppm) from tetramethylsilane (TMS) using the residual solvent resonance or TMS as the internal standard ($CDCl_3$: 7.26 ppm for ^1H NMR, 77.0 ppm for ^{13}C NMR). Multiplicities are abbreviated as follows: s = singlet, d = doublet, t = triplet, q = quartet, m = multiplet). IR spectra were recorded on Perkin–Elmer Spectrum One instrument at room temperature. Relative intensities of the signals are given as very strong (vs), strong (s), medium (m), weak (w) and broad (br). Mass spectra were recorded on Micro Mass LCT Premier Spectrometer from Waters. Optical rotations were determined on a Perkin Elmer polarimeter 343. Melting points (uncorrected) were determined on a Gallenkamp melting point apparatus MFB 595. CHN analyses were recorded on a Perkin–Elmer Elemental Analyzer 2400 CHN. Analytical TLC was carried out using precoated silica gel plates (Merck TLC plates silica gel 60 F_{254}).TLC plates were visualized under UV irradiation (254 nm) or with $KMnO_4$-solution. Flash column chromatography was performed using silica Merck silica gel 60 (0.040–0.063 mm). All water sensitive reactions were carried out in flame dried glassware under an argon atmosphere. Chemicals were purchased from Sigma-Aldrich or Acros and used without further purification. Grignard reagents were titrated with I_2/LiCl [3] before application. Solvents were dried as follows: THF was distilled over Na/benzophenone, CH_2Cl_2 was distilled over CaH_2 and MeCN was distilled over P_4O_{10}.

3 Krasovskiy, A.; Knochel, P. *Synthesis* **2006**, *5*, 890–891. DOI: 10.1055/s-2006-926345

3 Experimental procedures and analytical data

3.1 Synthesis of *N*-benzyl-*N*-carbamate-protected amino acids 2a–d (Scheme 1)

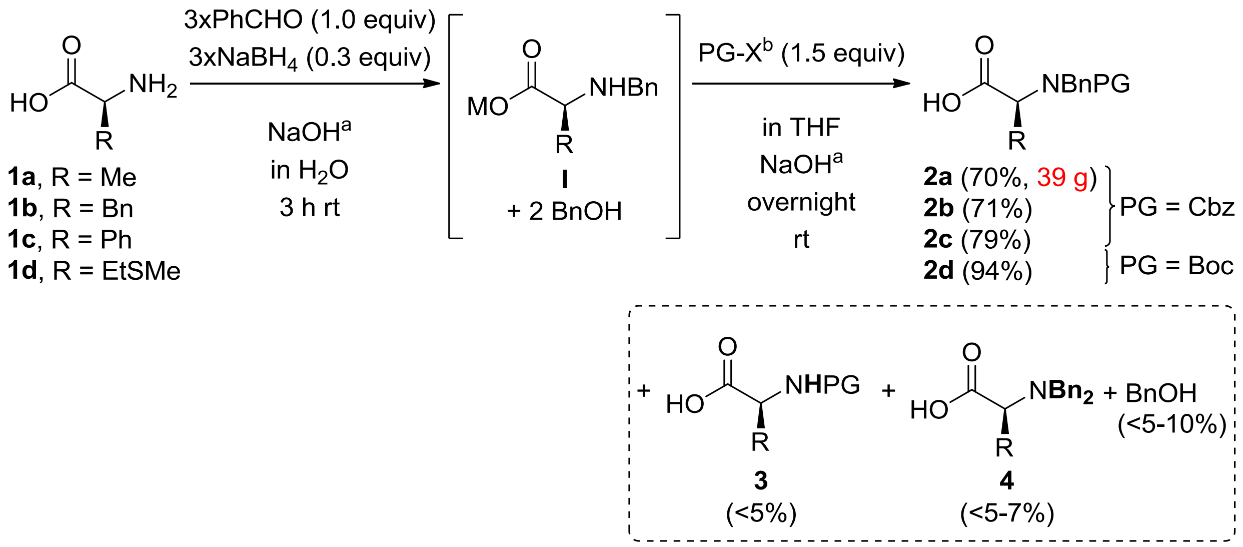

a) KOH for **1b**. b) PG-X = Cbz-Cl (**1a–c**), Boc_2O (**1d**)

General procedure: The amino acid **1** (1.0 equiv) and NaOH (1.0 equiv) [4] were dissolved in water ([**1**] = 2 mol/L for **1a,c,d**; 0.6 mol/L for **1b**). Then the following procedure was repeated for two times: Benzaldehyde (1.0 equiv) [5] was added and the reaction mixture was stirred for 15 min at room temperature. Under cooling with an ice bath $NaBH_4$ (0.3 equiv) was added in 2–3 portions within 5–10 min, the cooling bath was removed and the mixture was allowed to stir for 30 min at ambient temperature. Next, the pH value of the reaction mixture was adjusted to 10–11 via dropwise addition of 3 N HCl-solution (aq., 0.2–0.3 equiv; 6 N HCl was utilized in the large scale preparation of **2a**) under vigorous stirring. As with substrates **1b–d** a solid precipitate (the corresponding *N*-benzyl amino acid derivatives) formed during acidification, only as much HCl-solution was added, that magnetically stirring was still possible.

To ensure a quantitative benzylation, a third portion of benzaldehyde and $NaBH_4$ was added as described above. Subsequently, the reaction mixture was diluted with water to a concentration of [**I**] = 0.6 mol/L (in the large scale preparation of **2a** the reaction mixture was only diluted to 0.8 mol/L),

4. As with NaOH in the case of substrate **1b** a solid precipitate occurred during the following PhCHO/$NaBH_4$-treatment, which made magnetically stirring impossible, KOH was utilized here.

5. To remove BzOH 20 mL of benzaldhyde were diluted with 2 mL of Et_2O (in order to improve the phase separation), washed successively with three 10 mL portions of saturated $NaHCO_3$ and 10 mL of brine and dried over $MgSO_4$. After concentration under reduced pressure at the rotatory evaporator only small amounts of Et_2O remained (2–3 mol % according to ^{1}H NMR). The washing (or distillation) of benzaldehyde was crucial to avoid contamination of the product **2** with benzoic acid.

further NaOH (2.0 equiv) was added [4] and the mixture was stirred at room temperature until the hydroxide salt had dissolved completely (5–10 min). Under cooling to 0 °C a solution of the acylating agent (1.5 equiv, CbzCl for **2a–c** and Boc_2O for **2d**, respectively) in THF (volume ratio aqueous phase (= H_2O)/THF 3:1) was added dropwise through a dropping funnel. After stirring for 15 min at 0 °C the cooling bath was removed and the reaction suspension stirred overnight at ambient temperature (17–24 h).

Thereafter the reaction mixture was acidified to pH ≤ 0 under cooling to 0 °C through dropwise addition of 3 N HCl solution in water (6 N HCl was utilized in the large scale preparation of **2a**) and extracted with three portions of Et_2O (H_2O/Et_2O 2:1) [6]. The combined organic phases were dried over $MgSO_4$ and concentrated under reduced pressure. To remove BnOH the residue was dissolved in Et_2O ([**2**] = 1 mol/L), a small spatula tip of phenolphthalein was added, the solution was cooled in an ice bath and a 1.0 N LiOH solution (aq., ca. 1.0 equiv) was added dropwise, until a color change (colorless → violet) was observed (pH = 10) [7]. Then the aqueous phase was washed with one further portion of Et_2O (H_2O/Et_2O 2:1), three portions of EtOAc (first portion H_2O/EtOAc 1:1, second and third 2:1) and finally an additional portion of Et_2O (H_2O/EtOAc 2:1) [8], cooled in an ice bath and acidified through the addition of 3 N HCl solution (aq.; 6 N HCl was utilized in the large scale preparation of **2a**) after dilution with Et_2O (H_2O after HCl addition/Et_2O ca. 2:1). Next, the aqueous phase was extracted with two further portions of Et_2O (H_2O after HCl addition/Et_2O 2:1), the combined extracts were dried over $MgSO_4$ and concentrated under reduced pressure. Finally, the isolated acids **2** were dried in high vacuum under stirring and heating to 60 °C for several hours. Analytical data of derivatives **2a–c** was published in the Supporting Informations of [2].

(*S*)-2-(Benzyl(benzyloxycarbonyl)amino)propanoic acid (2a)

yield: 39.41 g (→ 117.5 mmol, 70%, considering 3% of residual BnOH, 1% of BzOH and 7% of *N,N*-dibenzylalanine **4a** according to ^{1}H NMR, colorless highly viscous oil).

(*S*)-2-(Benzyl(benzyloxycarbonyl)amino)-3-phenylpropanoic acid (2b)

yield: 8.56 g (→ 21.4 mmol, 71% considering 10% of residual BnOH, colorless highly viscous oil).

(*S*)-2-(Benzyl(benzyloxycarbonyl)amino)-2-phenylethanoic acid (2c)

---

6. In the case of the acid sensitive Boc-protected methionine derivative **2d** the first portion of Et_2O was added before acidification with aqueous HCl solution.

7. Without adjusting the pH carefully to 9-10, the isolated product **2** contained residual HOAc (resulting from saponification during EtOAc washings).

8. In the preparation of the protected phenylalanine derivative **2b** the crude mixture of the acid **2b** and BnOH alcohol was dissolved in Et_2O ([**2b**] = 2 mol/L) and basified as described. Phase separation of the resulting homogeneous solution (one phase!) was achieved by the addition of *c*-Hex (H_2O/*c*-Hex 2:1). Next, the aqueous phase was washed with one portion of Et_2O (H_2O/Et_2O 2:1) and three portions of EtOAc/Et_2O 1:1 (H_2O/organic phase 2:1). The work up was then continued as described in the general procedure (dilution with Et_2O and acidification).

yield: 20.84 g (→ 52.1 mmol, 79%, considering 10% of residual BnOH and 8% of EtOAc, colorless highly viscous oil).

(*S*)-2-(Benzyl(benzyloxycarbonyl)amino)-4-(methylthio)butanoic acid (2d)

yield: 32.58 g (→ 94.2 mmol, 94%, considering 6% of residual BnOH, colorless solid).

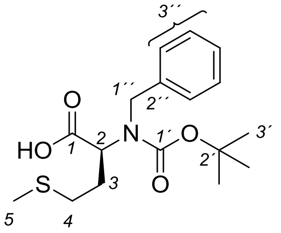

M ($C_{17}H_{25}NO_4S$) = 339.45 g/mol; **mp.** = 63.9°C; **^{1}H NMR** ($CDCl_3$, 400 MHz, TMS, mixture of rotamers): δ [ppm] = 11.20 (s (broad), 1H, COOH), 7.40-7.24 (m, 5H, H-3´´), 4.71-4.64 (m, 1H, H-1´´$_a$), 4.54-4.10 (m, 2H, H-2, H-1´´$_b$), 2.40-2.25/2.10-1.97 (2xm, 4H, H-3, H-4), 1.93 (s, 3H, H-5) 1.46 (s, 9H, H-3´´); **^{13}C NMR** (100 MHz, $CDCl_3$, mixture of rotamers) δ [ppm] = 177.5/176.4 (C-1), 156.1/155.4 (C-1´), 130.2 (C-2´´), 128.6/128.5/127.5 (C-3´´), 81.4 (C-2´), 59.2/58.1 (C-2), 52.1 (C-1´´), 31.00/29.7/28.8/26.7 (C-3, C-4), 28.3 (C-3´), 15.0 (C-5); **IR** [cm^{-1}] = 3181 (br), 3085 (w), 3061 (w), 3025 (w), 3002 (w), 2973 (m), 2914 (w), 2604 (w), 2356 (w), 1739 (m), 1696 (s), 1605 (w), 1585 (w), 1494 (m), 1452 (s), 1421 (s), 1392 (m), 1365 (s), 1316 (w), 1250 (s), 1156 (s), 1076 (m), 1028 (m), 957 (m), 909 (m), 860 (m), 819 (w), 770 (m), 732 (s), 698 (s), 668 (w); **$[\alpha]_D$** (c = 1.31 g/100 mL, $CHCl_3$, T = 20.0 °C)= -49.0.

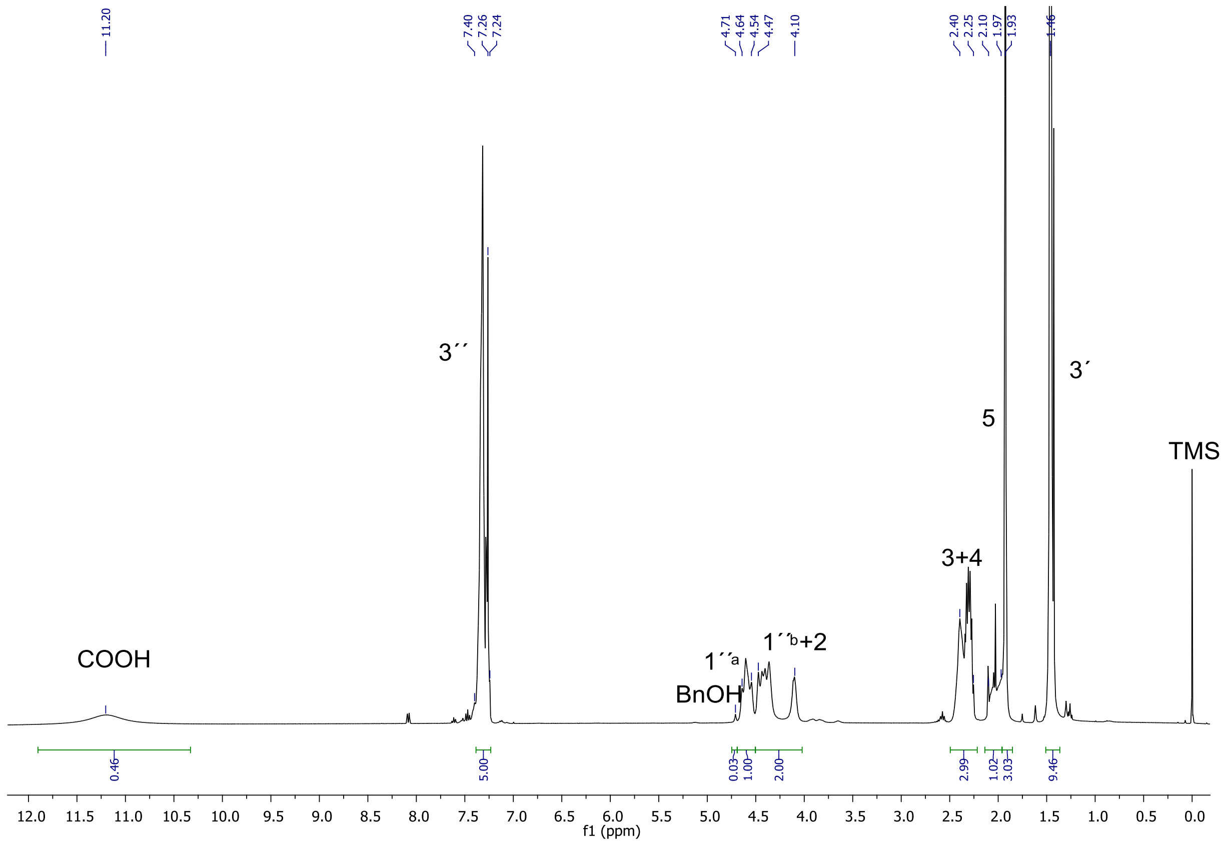

^{1}H NMR-spectra of the methionine derivative **2d** (400 MHz, $CDCl_3$).

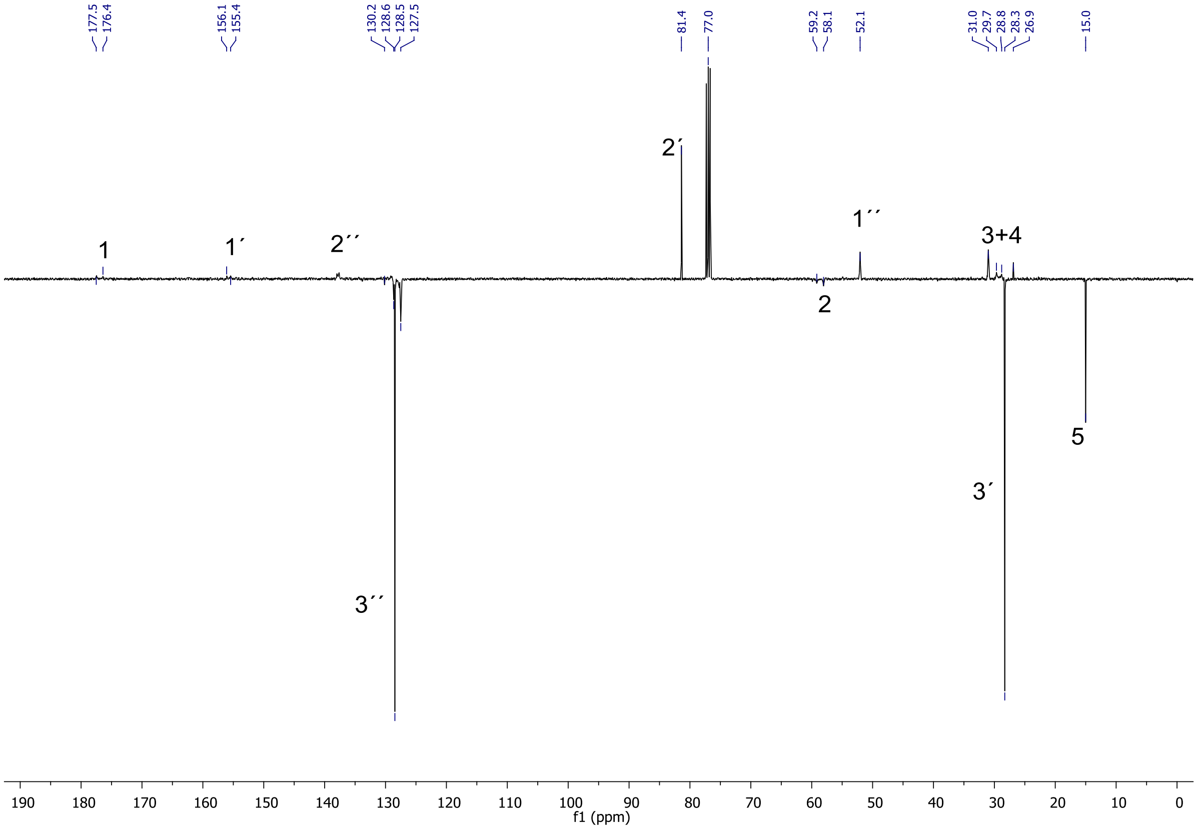

APT-NMR-spectra of the methionine derivative **2d** (100 MHz, $CDCl_3$).

3.1.1 Synthesis of (*S*)-2-(benzyl(benzyloxycarbonyl)amino)propanoic acid in two steps (2a)

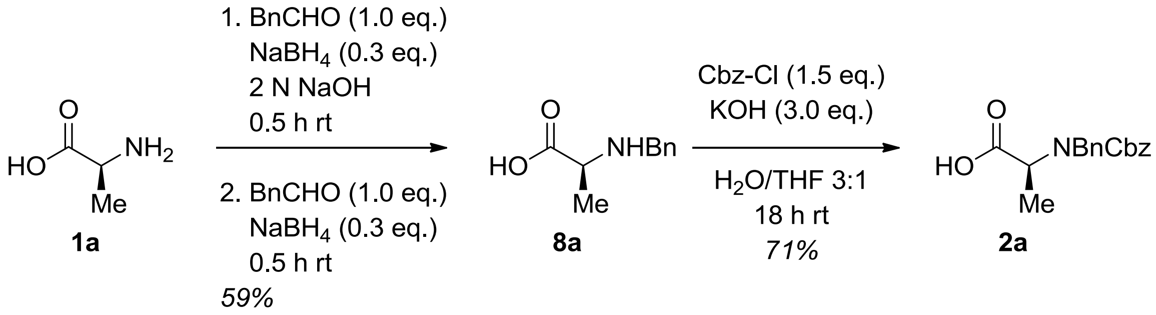

Experimental procedures and analytical data are given in the Supporting Informations of [2].

3.2 Synthesis of Weinreb amides 5a–d (Scheme 2 and Table 1)

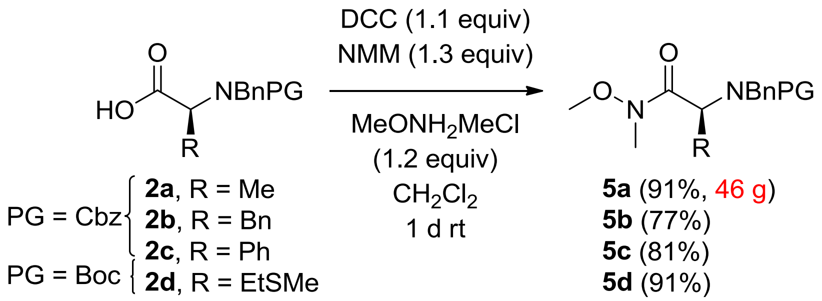

General procedure: The acid **2** (1.0 equiv) was dissolved in reagent grade CH_2Cl_2 ([**2**] = 0.3–0.4 mol/L), cooled in an ice bath and NMM (1.3 equiv) and $MeONH_2MeCl$ (1.2 equiv) were added successively. Then DCC (1.1 equiv) was added to the reaction mixture in one portion and the mixture was allowed to stir for 30 min at 0 °C and for 1 d at ambient temperature.

From the reaction suspension was evaporated CH_2Cl_2 under reduced pressure (in order to precipitate urea completely), the residue was partitioned between EtOAc (**5a** and **5d**)/Et_2O (**5b** and **5c**) ([**5**] = 1 mol/L) and 1 N HCl solution in water (volume ratio aqueous phase (= H_2O)/organic phase 1:1→1 mL/1 mmol **5**) and, stirred for 5 min at room temperature to convert the excess of DCC to the corresponding urea, and passed through a sintered funnel. The residue (urea) was rinsed with two portions of EtOAc (**5a** and **5d**) and Et_2O (**5b** and **5c**), respectively, (1 mL/2 mmol **5**), the organic phase (separated from the collected filtrates) was washed successively with 1 N HCl solution (aq.), saturated $NaHCO_3$-solution (aq.) and brine (each one portion, 1 mL/1 mmol **5**), dried over $MgSO_4$ and concentrated under reduced pressure. To remove remaining *N,N´*-dicyclohexylurea the crude amides **5a** and **5d** were up taken in *c*-Hex ([**5**] = 1 mol/L) and passed through a sintered funnel, the precipitated urea was washed with two small portions of *c*-Hex and the combined filtrates were concentrated in vacuo. In contrast, the crude amides **5b** and **5c** were purified through column chromatography on silica gel (SiO_2/crude weight 10–15:1) with EtOAc/*n*-Hex, whereby crude **5** was dissolved in a minimum amount of the eluent. Finally, the amides **5a–d** were dissolved in *c*-Hex (ca. 1 mL/3 mmol **5**) and concentrated under reduced pressure to remove EtOAc (2x) and dried in high vacuum for several hours under stirring and heating to 40 °C. Analytical data and HPLC chromatograms of derivatives **5a–c** are given in the Supporting Informations of [2].

(*S*)-*N*-Methyl-*N*-methoxy-2-(benzyl(benzyloxycarbonyl)amino)propanoic amide (5a)

yield: 45.60 g (128.1 mmol, 91%, pale yellow oil, ee ≥ 99%) without purification.

(*S*)-*N*-Methyl-*N*-methoxy-2-(benzyl(benzyloxycarbonyl)amino)-3-phenylpropanoic amide (5b)

yield: 11.80 g (27.3 mmol, 77%, colorless oil, ee ≥ 99%) after chromatographic purification with EtOAc/*n*-Hex 2:7.

(*S*)-*N*-Methyl-*N*-methoxy-2-(benzyl(benzyloxycarbonyl)amino)-2-phenylethanoic amide (5c)

yield: 19.60 g ($\rightarrow$39.33 mmol, 72% considering 4 mol % of residual *c*-Hex, colorless oil, ee = 95%) after chromatographic purification with EtOAc/*n*-Hex 4:9.

1.750 g ($\rightarrow$4.11 mmol, 81% taking 8 mol % of residual 3-Cl*n*-PrOH into account, colorless oil, ee = 95%) after chromatographic purification.

(*S*)-*N*-Methyl-*N*-methoxy-2-(benzyl(benzyloxycarbonyl)amino)-4-(methylthio)butanoic amide (5d)

yield: 7.71 g (20.16 mmol, 91%, colorless oil) without purification.

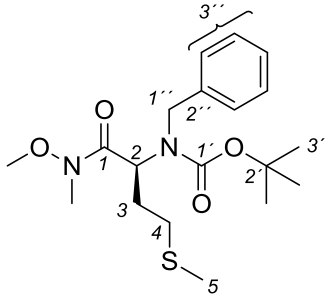

M ($C_{19}H_{30}N_2O_4S$) = 382.52 g/mol; **r_f** (SiO_2) = 0.40 (EtOAc/*c*-Hex 1:2); **^{1}H NMR** ($CDCl_3$, 400 MHz, mixture of rotamers) δ [ppm] = 7.36-7.19 (m, 5H, H-3´´), 5.39/5.17 (2xapp. s, 1H, H-2), 4.62-4.33 (m, 2H, H-1´´), 3.71/3.63 (2xs, 3H, OMe), 2.98/2.86 (2xs, 3H, NMe), 2.48-2.34 (m, 2H, H-4), 2.16-2.09 (m, 1H, H-3_a), 2.00 (s, 3H, H-5), 1.94-1.82 (m, 1H, H-3_b), 1.54/1.39 (2xs, 9H, H-3´); **^{13}C NMR** ($CDCl_3$, 100 MHz, mixture of rotamers) δ [ppm] = 171.4 (C-1, extracted from HMBC), 155.7 (C-1´), 139.4/138.8 (C-2´´), 128.6/128.1/128.0/127.9/127.0/126.7/126.6 (C-3´´), 80.3 (C-2´), 61.3 (OMe), 54.0/53.3 (C-2), 47.4/47.1 (C-1´´), 30.5 (NMe), 28.9 (C-4), 28.3/26.8 (C-3), 28.1 (C-3´), 15.0 (C-5); **IR** (ATR) ν [cm^{-1}] = 3086 (w), 3060 (w), 3027 (w), 2998 (w), 2971 (s), 2930 (s), 2863 (w), 2823 (w), 1689 (s), 1662 (s), 1603 (w), 1584 (w), 1533 (w), 1494 (m), 1452 (s), 1433 (s), 1401 (s), 1389 (w), 1364 (s), 1348 (w), 1319 (s), 1292 (m), 1272 (m), 1251 (s), 1162 (s), 1113 (s), 1073 (m), 1029 (m), 994 (s), 957 (m), 892 (m), 860 (m), 834 (w), 816 (w), 768 (m), 733 (s), 697 (s), 667 (w); **GC-MS** (EI, 70 eV) m/z [u] = 308 (5, [M-$CH_2CH_2SCH_3$]$^+$) 281 (5, [M-Boc]$^+$), 252 (10), 209 (10), 194 (50), 146 (10, [M-O*t*Bu-Bn- $CH_2CH_2SCH_3$]$^+$), 91 (100, [Bn]$^+$), 57 (25, [*t*Bu]$^+$); **HR-MS** (ESI, [$C_{19}H_{30}N_2O_4SNa$]$^+$) calc. 405.1818 u found 405.1817 u; **[α]$_D$** (c = 1.61 g/100 mL, $CHCl_3$, T = 20.0 °C) = -47.6.

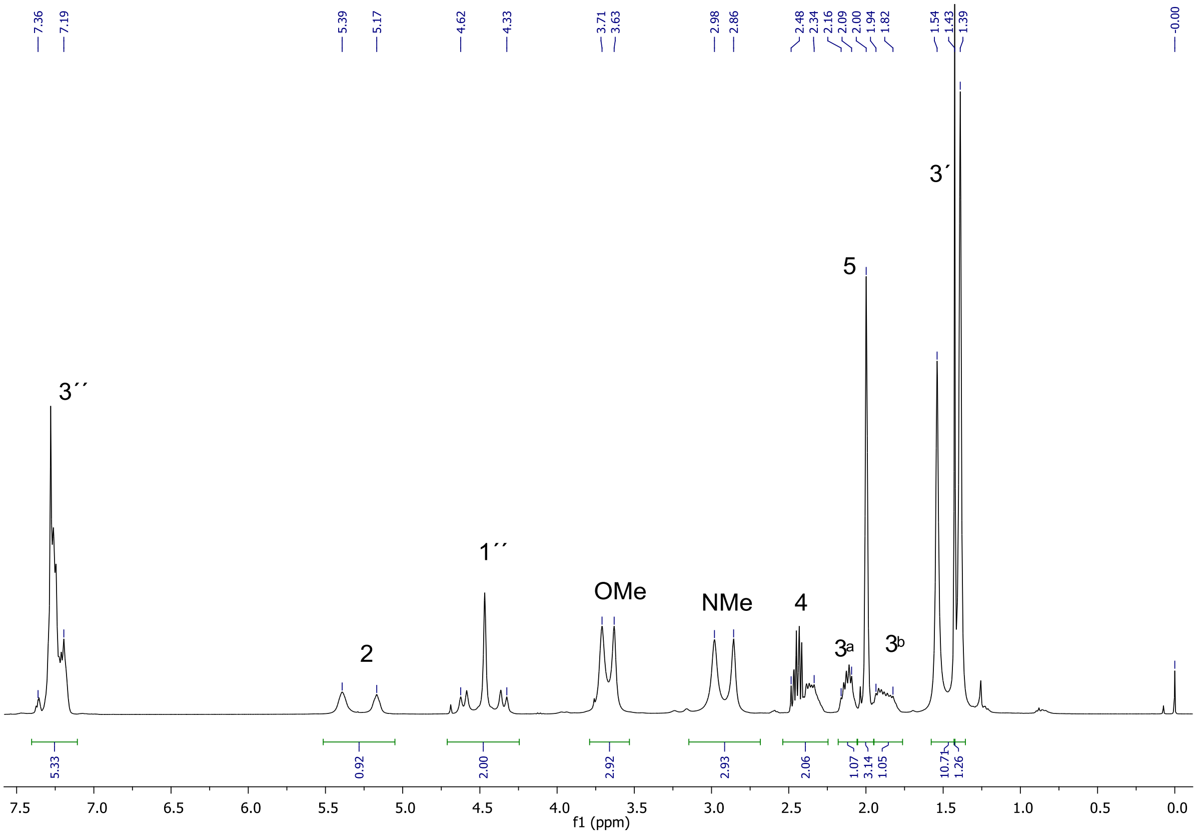

^{1}H NMR-spectra of the methionine derivative **5d** (400 MHz, $CDCl_3$).

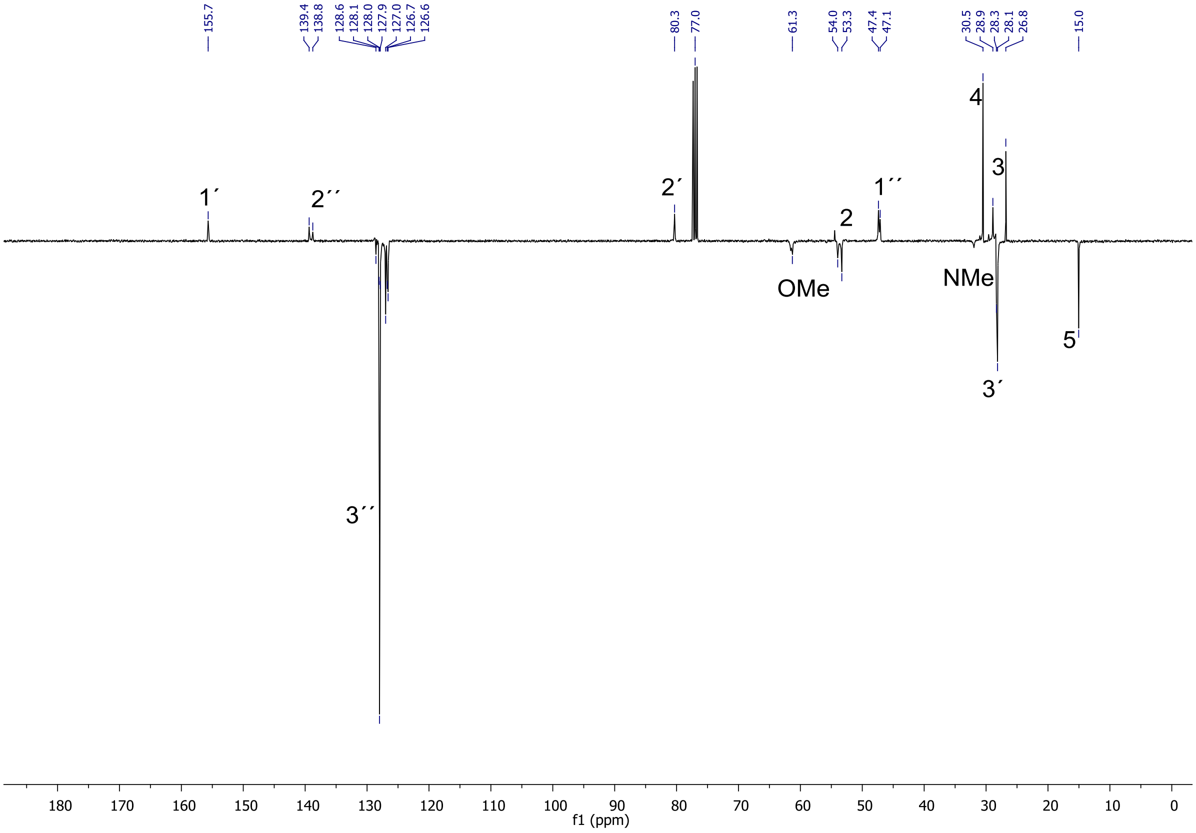

APT-NMR-spectra of the Weinreb amide **5d** (100 MHz, $CDCl_3$).

3.3 Synthesis of hydroxyketones 7a–d (Scheme 2)

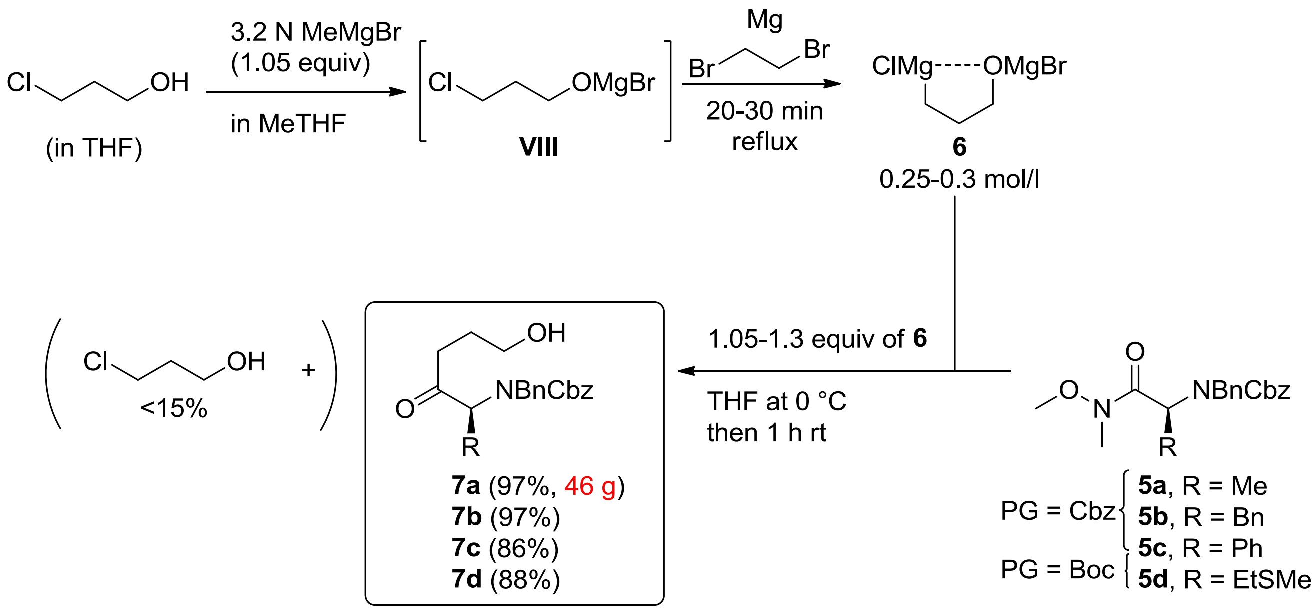

General procedure: Under an atmosphere of argon (balloon) a flame-dried three-necked flask equipped with reflux condenser, dropping funnel and a rubber septum was charged with dry THF (total volume of THF/MeTHF after addition of the MeMgBr solution 2.5 mL/1 mmol Cl*n*PrOH → [Cl*n*PrOH] = 0.4 mol/L) and 3-chloropropanol (1.0 equiv) and cooled to 0 °C. Then a commercial, dark brown MeMgBr solution in 2-MeTHF (1.05 equiv, titer determined by the I_2/LiCl method [3]) [9,10] was added dropwise through the dropping funnel accompanied by a methane evolution (in large scale preparations the methane pressure was released in between). An excess of MeMgBr was indicated by a color change from colorless to pale yellow at the end of the MeMgBr addition. Then the cooling bath was removed and the reaction solution containing alcoholate **VII** was allowed to warm to ambient temperature (over 5 min). Next magnesium (2.0 equiv) and 1,2-dibromoethane (0.1 equiv) were added both in one portion, whereby the latter one effected a slight change to a greenish color and initiated the exothermic Grignard formation, and the mixture was allowed to stir for 15 min at ambient temperature. During this time an oil bath was preheated to 90 °C. Subsequently, the mixture was heated to reflux for 20–30 min in order to complete the Grignard formation. If the reaction suspension refluxed too violently (due to the exothermic Grignard formation), heating was intercepted. The resulting dark grey Grignard

9. In contrast to the authors the saturated LiCl solution in THF was not prepared in prior (stirring overnight). Instead an excess of LiCl (>100 mg) and an exactly weighed amount of Iodine (30–350 mg, depending on the concentration of the Grignard reagent) were stirred in 5 mL of dry THF for a few minutes before titration.

10. Commercial MeMgBr solution in MeTHF was preferred over MeMgBr solutions in THF due to their higher concentrations (3.2 N compared to 1.0 N). Nevertheless, with commercial Grignard solution in THF comparable concentrations of the reagent **6** were achieved.

solution usually showed a concentration of 0.25–0.30 mol/L determined with I_2/LiCl [3,9].The freshly prepared reagent **6** was used further within 1 d after preparation.

In a separate flask equipped with a dropping funnel with volumetric scale the amide **5** was dissolved in dry THF ([**5**] = 2 mol/L) under an atmosphere of argon. Then the freshly prepared solution of reagent **6** (1.05–1.3 equiv) was transferred to the dropping funnel under a positive argon pressure, the solution of the substrate **5** was cooled in an ice bath and the reagent **6** was added dropwise. Thereafter the cooling bath was removed and the reaction solution was stirred for 1 h at ambient temperature, whereupon micro work up (ca. 50 μL of the reaction mixture) with aqueous saturated NH_4Cl-solution and EtOAc or Et_2O and TLC-control (EtOAc/*n*-Hex 1:1) usually indicated full conversion.

Then the reaction solution was cooled to 0 °C, saturated aq. NH_4Cl solution (2 mL NH_4Cl (aq.)/1 mmol **6**) was added through the dropping funnel and THF/MeTHF were evaporated under reduced pressure (→200 mbar). The residue was diluted with a minimum amount of water to dissolve precipitated NH_4Cl (ca. 1/2–1/3 of the volume of the NH_4Cl-solution) and Et_2O (H_2O/Et_2O 2:1, pH of the aqueous phase 10–12). If a ($Mg(OH)_2$) precipitation still remained, the mixture was acidified either with HOAc (1 mL/4 mmol **6**; precipitated NH_4OAc had to be dissolved with water) or 3 N HCl solution in water until dissolution of the solid (pH = 6–7). Afterwards, the aqueous phase was extracted with two further portions of Et_2O (H_2O/Et_2O 2:1), the collected Et_2O-phases were washed with brine (brine/Et_2O 1:3), dried over $MgSO_4$ and concentrated under reduced pressure. Eventually, crude **7** was purified by column chromatography on silica gel (crude **7**/SiO_2 1:10–20) with EtOAc/Hex mixtures, whereby the crude product was dissolved in eluent (see below). Finally, the ketone **7** was diluted with *c*-Hex ([**7**] = ca. 2 mol/L) and concentrated under reduced pressure to remove EtOAc (2x) and dried in high vacuum under stirring and heating to 40 °C for several hours. Analytical data of derivatives **7a–c** and HPLC chromatograms of **7c** were published in the Supporting Informations of [2].

(*S*)-2-(Benzyl(benzyloxycarbonyl)amino)-6-hydroxy-3-hexanone (7a)

yield: 46.68 g (85% yield over two steps after further conversion to the amino alcohol *syn*-9a, pale yellow oil) without chromatographic purification.

yield: 16.29 g (→43.5 mmol, 97% considering 9 mol % of residual *c*-Hex and 6 mol % of unreacted 3-chloropropanol according to ^{1}H NMR, colorless oil) after chromatographic purification with EtOAc/*n*-Hex 1:1.

(*S*)-2-(Benzyl(benzyloxycarbonyl)amino)-6-hydroxy-1-phenyl-3-hexanone (7b)

yield: 3.71 g (→8.28 mmol, 97% considering 1 mol % of residual *c*-Hex and 15 mol % of 3-chloropropanol, colorless oil) without chromatographic purification.

(*S*)-1-(Benzyl(benzyloxycarbonyl)amino)-5-hydroxy-1-phenyl-2-penatanone (7c)

yield: 10.26 g (→23.6 mmol, 86% taking 5 mol % of residual *c*-Hex and 14 mol % of Cl*n*PrOH into account, pale yellow oil, ee = 92%) after chromatographic purification with EtOAc/*c*-Hex 4:5 as a pale yellow viscous oil.

(*S*)-3-(Benzyl(benzyloxycarbonyl)amino)-7-hydroxy-1-(methylthio)-4-heptanone (7d)

yield: 3.91 g (→ 10.13 mmol, 88% considering 5 mol % Cl*n*PrOH, colorless oil) after chromatographic purification with EtOAc/*c*-Hex 3:2.

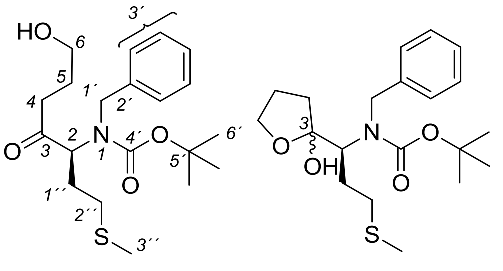

M ($C_{20}H_{31}NO_4S$) = 381.52 g/mol; $\mathbf{r_f}$ (SiO_2) = 0.55 (EtOAc); **^{1}H NMR** (400 MHz, $CDCl_3$, TMS, mixture of isomers and rotamers): δ [ppm] = 7.43-7.27 (m, 5H, H-3´), 4.88/4.69 (2xd, 1H, H-1´$_a$, J = 14.7/14.7 Hz), 4.21 (d, 1H, H-1, J = 14.8 Hz), 4.13/4.01/3.88 (app. t/d/app. T, 1H, H-2, J = 6.3/14.3, 7.6/5.6 Hz), 3.68-3.41 (m, 2H, H-6), 2.45-2.32/2.12-1.74 (2xm, 10H, H-4, H-1´´, H-2´´, H-3´´, OH; amongst s (H-3´´) at 1.74 ppm), 1.70-1.54 (m, 2H, H-5), 1.49/1.44 (2xs, 9H, H-6´); **^{13}C NMR** (100 MHz, $CDCl_3$, mixture of isomers and rotamers): δ [ppm] = 208.2/208.1 (C-3$_{ketone}$), 155.4/155.0 (C-4´), 137.8/137.7 (C-2´), 129.2/128.6/128.5/2x128.3/127.85/127.7/127.5 /126.9 (C-3´), 81.5/81.3 (C-5´), 63.7/63.6 (C-2), 62.1/61.78 (C-6), 51.6/51.2 (C-1´), 35.4/35.2 (C-4), 31.3/31.2 (C-2´´), 28.4/28.3/28.2 (C-6´), 27.5 (C-1´´), 26.6, 26.5 (C-5), 15.2/15.1 (C-3´´); signals of the furane tautomer: 107.4 (C-3$_{acetale}$), 67.2, 65.23, 60.3, 59.3, 41.7, 33.9, 30.1; **HR-MS** (ESI, $[C_{20}H_{31}NO_4SNa]^+$) calc. 404.1866 u found 404.1867 u; **[α]$_D$** (c = 1.21 g/100 mL, $CHCl_3$, T = 20.0 °C) = -85.5.

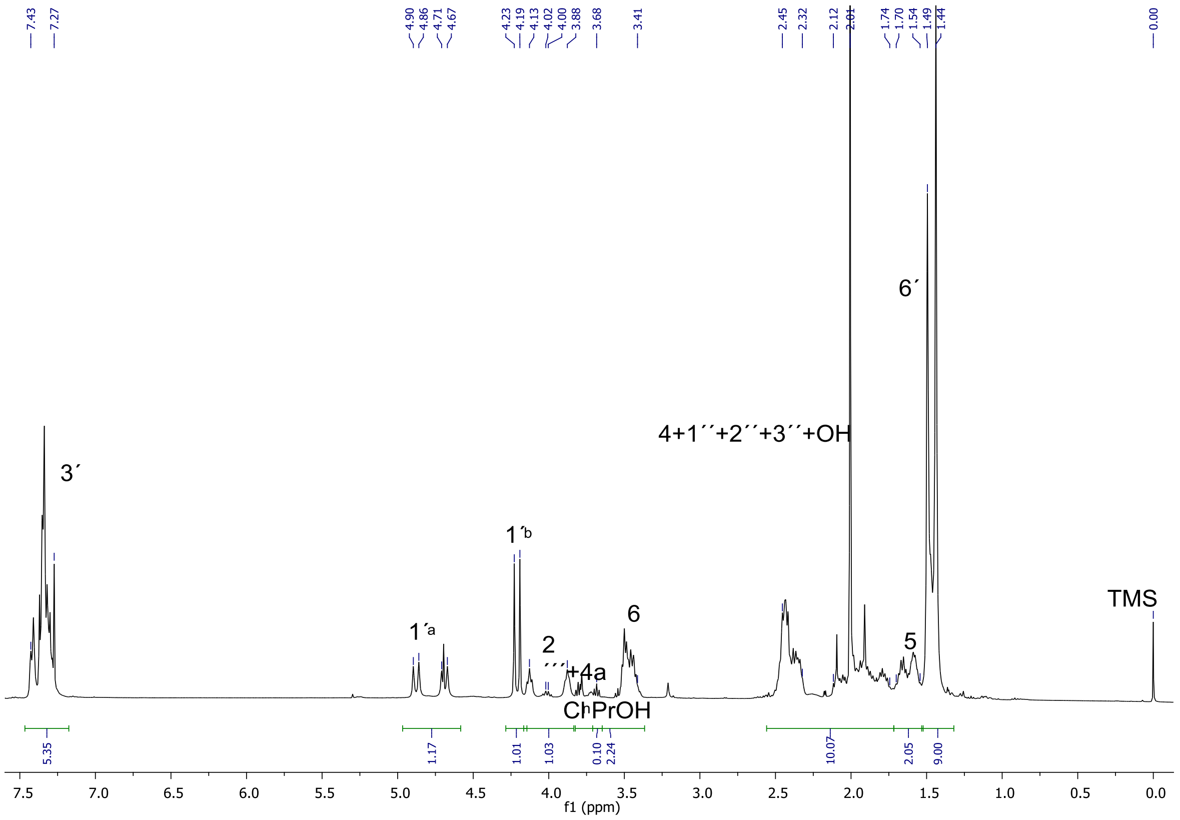

^{1}H NMR-spectra of the hydroxy ketone **7d** (400 MHz, $CDCl_3$).

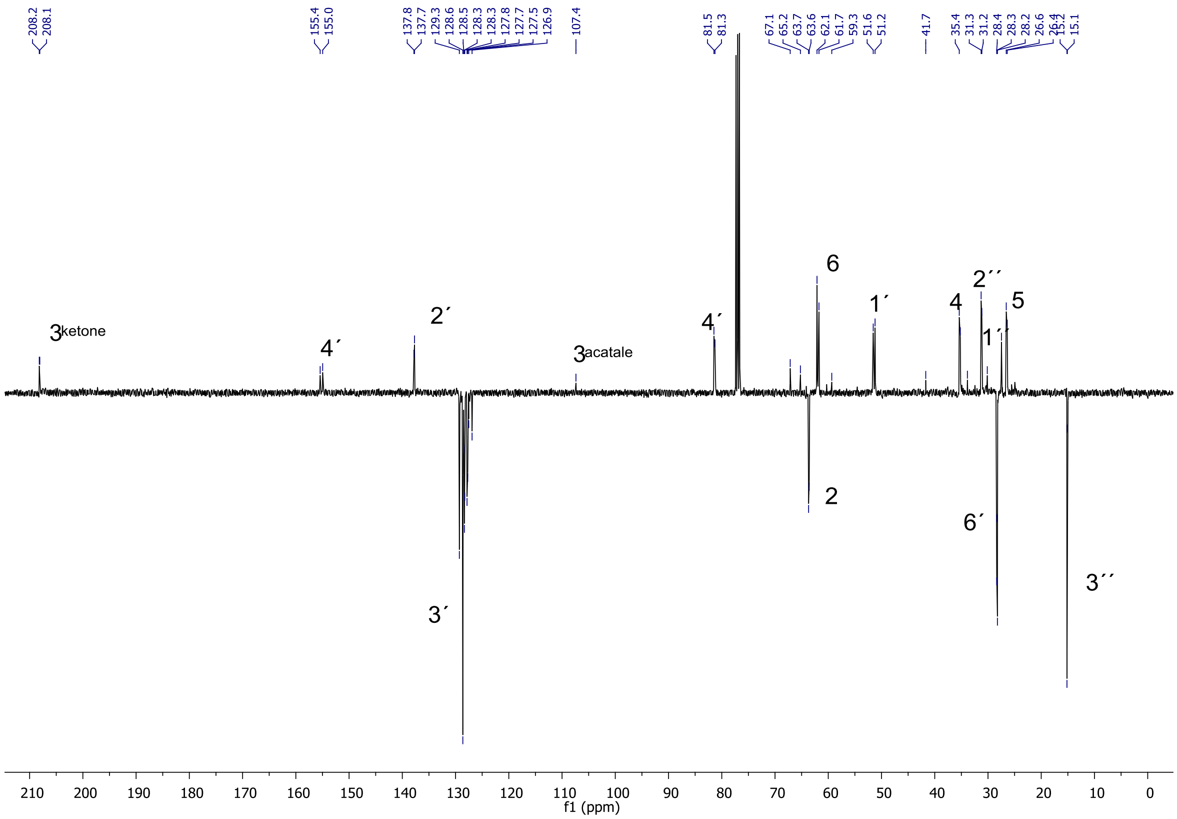

APT-NMR-spectra of the hydroxy ketone **7d** (100 MHz, $CDCl_3$).

3.4 Synthesis of glutamic and aspartic acid Derivatives 7e and 5f (Scheme 3)

3.4.1 Synthesis of (*S*)-2-(benzylamino)pentanedioic acid 5-methylester (8e)

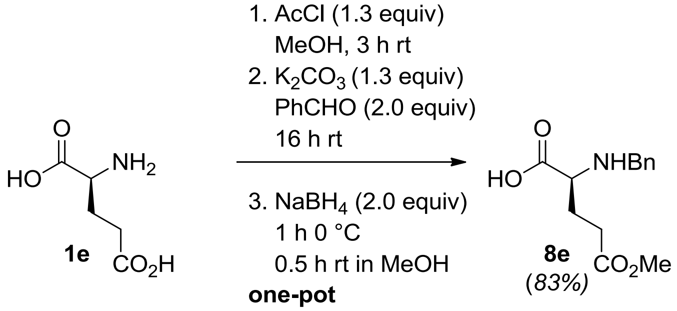

Glutamic acid **1** (5.00 g, 34.21 mmol, 1.0 equiv) was suspended in MeOH (70 mL, [**1e**] = 0.5 mol/L), cooled in an ice bath and AcCl (3.2 mL, 44.5 mmol, 1.3 equiv) was added dropwise. After 0.25 h of stirring the cooling bath was removed and the reaction solution allowed to stir for 3 h at ambient temperature [11]. Then the mixture was cooled again to 0 °C and K_2CO_3 (6.14 g, 44.5 mmol, 1.3 equiv)

11. Thereby, the reaction progress was monitored through concentration of a small aliquot of the reaction in vacuo and ^{1}H-NMR in MeOH-d_4.

and successively (7.0 mL, 68.4 mmol, 2.0 equiv) of PhCHO were added portionwise (weak CO_2 evolution). The cooling bath was removed and the reaction suspension was allowed to stir for 16 h at room temperature. Finally, the reaction mixture was cooled in an ice bath and (2.59 g, 68.4 mmol, 2.0 equiv) of $NaBH_4$ were added in six portions over 30 min. Subsequently, the reaction suspension was stirred for 0.5 h at 0 °C and 0.5 h at room temperature.

Under cooling to 0 °C 32% HCl solution in water (8.7 mL, 89.0 mmol, 2.6 equiv) was added carefully (CO_2-evolution), the mixture was stirred for 5 min at ambient temperature and MeOH was removed under reduced pressure. The colorless solid residue was suspended under vigorously shaking in water (50 mL, pH of the mixture was 5–6), cooled to 0 °C to complete the precipitation of the product **8e**. The precipitate was collected by filtration (through a sintered funnel), the reaction flask was rinsed with the filtrate, cooled in an ice bath and remaining product **8e** was again passed through the same sintered funnel containing already product from the first filtration. The combined precipitates were successively washed with ice cold water (40 mL) and ice cold acetone (50 mL). The collected precipitates were transferred to a 100 mL NS 29 flask, dried in high vacuum (28 h) and pestled (in the flask) several times with a magnetic stir bar retriever, until the product was obtained as a fine powdered solid. Finally, the benzyl amino acid **8e** (7.100 g, 28.26 mmol, 83%) was isolated as a colorless solid.

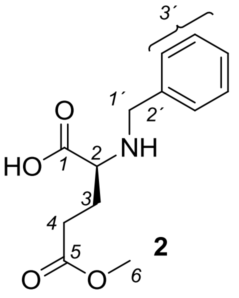

2

M ($C_{13}H_{17}NO_4$) = 251.278 g/mol; **mp.** = 154 °C (decomposition), lit. 163 °C [12]; **^{1}H NMR** (400 MHz, D_2O) δ [ppm] = 7.53-7.48 (m, 5H, H-3´), 4.30 (d, 1H, H-1´$_a$, J = 12.8 Hz), 4.21 (d, 1H, H-1´$_b$, J = 12.8 Hz), 3.69 (s, 3H, H-6), 3.66 (dd, 1H, H-2, J = 7.7, 5.4 Hz), 2.61-2.48 (m, 2H, H-4), 2.24-2.06 (m, 2H, H-3); ^{13}C NMR (100 MHz, D_2O, $CHCl_3$ capillar as standard, chemical shifts extracted from HMQC and HMBC) δ [ppm] = 175.6 (C-5), 173.0 (C-1), 130.1/129.7/129.3 (C-3´), 60.8 (C-2), 52.2 (C-6), 50.2 (C-1´), 29.6 (C-4), 24.6 (C-3); **$[\alpha]_D$** (c = 1.105 g/100 mL, 1 N HCl in H_2O, T = 22.0 °C) = +20.4; lit.: **$[\alpha]_D^{25}$** (c = 1 g/100 mL, 1 N HCl in H_2O) +18.8 [12].

12. Hannachi, J.-C.; Vidal, J.; Mulatier, J.-C.; Collet, A. *J. Org. Chem.* **2004**, *69*, 2367-2373.

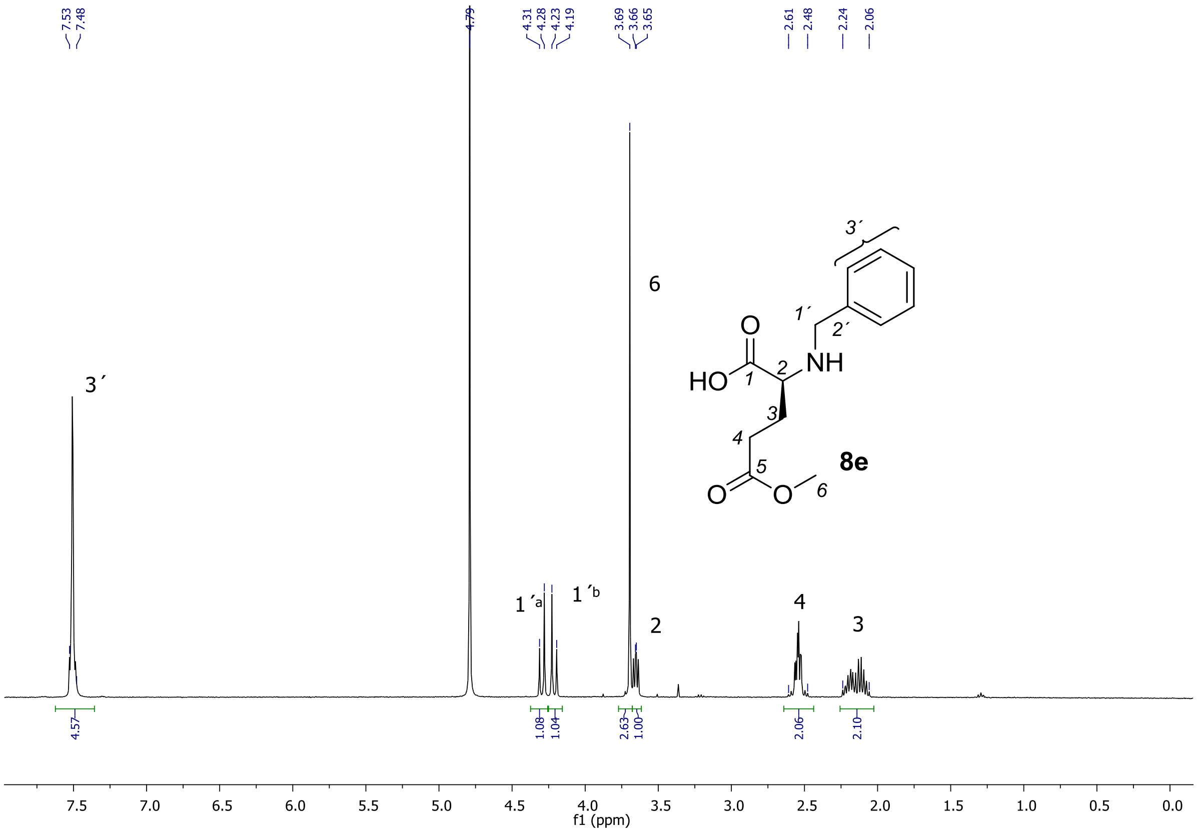

^{1}H NMR-spectra of the benzyl amine **8e** in D_2O (400 MHz).

3.4.2 Synthesis of (*S*)-2-(benzylamino)butanedioic acid 4-methylester (8f)

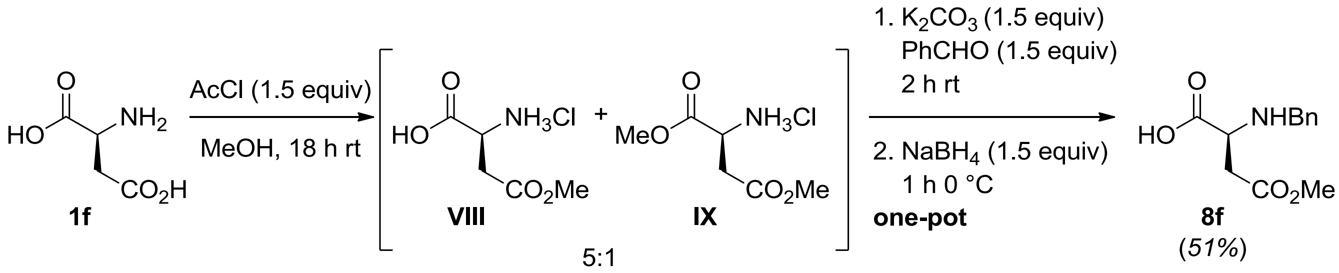

A suspension of aspartic acid **1f** (4.00 g, 30.05 mmol, 1.0 equiv) in MeOH (60 mL, [**1f**] = 0.5 mol/L, in a 250 mL flask with a strong stirring bar) was cooled in an ice bath and AcCl (3.2 mL, 45.08 mmol, 1.5 equiv) was added drop wise. After 0.5 h of stirring, the cooling bath was removed and the reaction solution was allowed to stir for 18 h at ambient temperature [13]. Then the mixture was cooled again to 0 °C and K_2CO_3 (6.22 g, 45.08 mmol, 1.5 equiv) was added portionwise (weak CO_2 evolution). After addition of (4.6 mL, 45.1 mmol, 1.5 equiv) of PhCHO, the cooling bath was removed and the reaction mixture was stirred for 2 h at room temperature (pH = 6). Finally, the reaction mixture was cooled in an ice bath and $NaBH_4$ (1.70 g, 45.1 mmol, 1.5 equiv) was added in six portions over 20 min.

13. After 18 h of stirring at ambient temperature, 0.2 mL of the reaction solution were separated and concentrated under reduced pressure. ^{1}H-NMR in MeOH-d^4 indicated full conversion of **1f** to Asp(OMe)-HCl/Asp(OMe)-OMe-HCl in a ratio of 5:1.

After stirring for 0.5 h, 32% HCl solution in water (8.8 mL, 90.15 mmol, 3.0 equiv) was added carefully (strong CO_2 evolution), the cooling bath was removed and MeOH was evaporated under reduced pressure at the rotatory evaporator. The residue was suspended under vigorously shaking in water (15 mL, pH of the mixture 2), the mixture was stirred for 5 min at 0 °C and the solid was collected by filtration (sintered funnel). The filtrate was treated with aqueous 2 N NaOH solution (6 mL) under cooling to 0 °C (pH = 6), whereby a colorless solid precipitated (**8f**) again. The reaction flask was rinsed with the filtrate and the newly precipitated product **8f** was collected through filtration through the same sintered funnel containing already product from the first filtration. The combined precipitates were successively washed with ice cold water (20 mL) and ice cold acetone (2 x 20 mL). The collected precipitates were transferred to a 100 mL NS 29 flask, dried in high vacuum 3 h and pestled (in the flask) several times with a magnetic stir bar retriever, until the product was obtained as a fine powdered solid. Finally, the benzyl amino acid **8f** (3.606 g, 15.20 mmol, 51%) was isolated as a colorless solid.
M $C_{12}H_{15}NO_4$) = 237.252 g/mol; **mp.** = 190 °C (decomposition), lit.: 219 °C [14]; **^{1}H NMR** (400 MHz, D_2O) δ [ppm] = 7.52 (s, 5H, H-3´), 4.39 (d, 1H, H-1´$_a$, *J* = 13.1 Hz), 4.31 (d, 1H, H-1´$_b$, *J* = 13.1 Hz), 3.97 (t, 1H, H-2, *J* = 5.8 Hz), 3.73 (s, 3H, H-5), 3.03 (d, 2H, H-3, *J* = 5.8 Hz); **^{13}C NMR** (100 MHz, D_2O, $CDCl_3$ capillar as standard) δ [ppm] = 172.3/171.9 (C-1, C-4), 130.5 (C-2´), 130.1/129.9/129.4 (C-3´), 57.1 (C-2), 52.8 (C-5), 50.7 (C-1´), 34.2 (C-3); **$[\alpha]_D$** (c = 0.960 g/100 mL, 1 N HCl in H_2O, T = 22.0 °C) = +11.5.

14. For racemic **8f** see: Zilkha, A.; Bachi, B. *J. Org. Chem.* **1959**, *24*, 1096-1098.

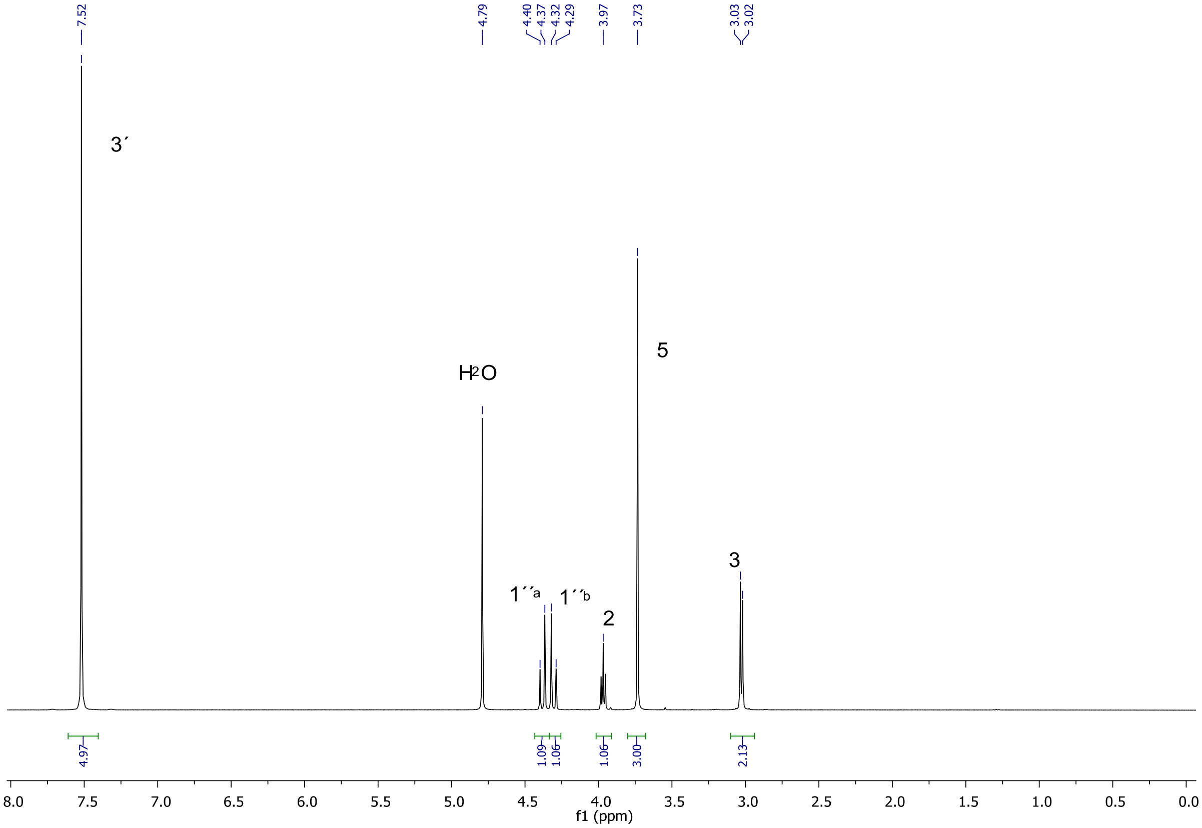

^{1}H NMR-spectra of the benzyl amine **8f** in D_2O (400 MHz).

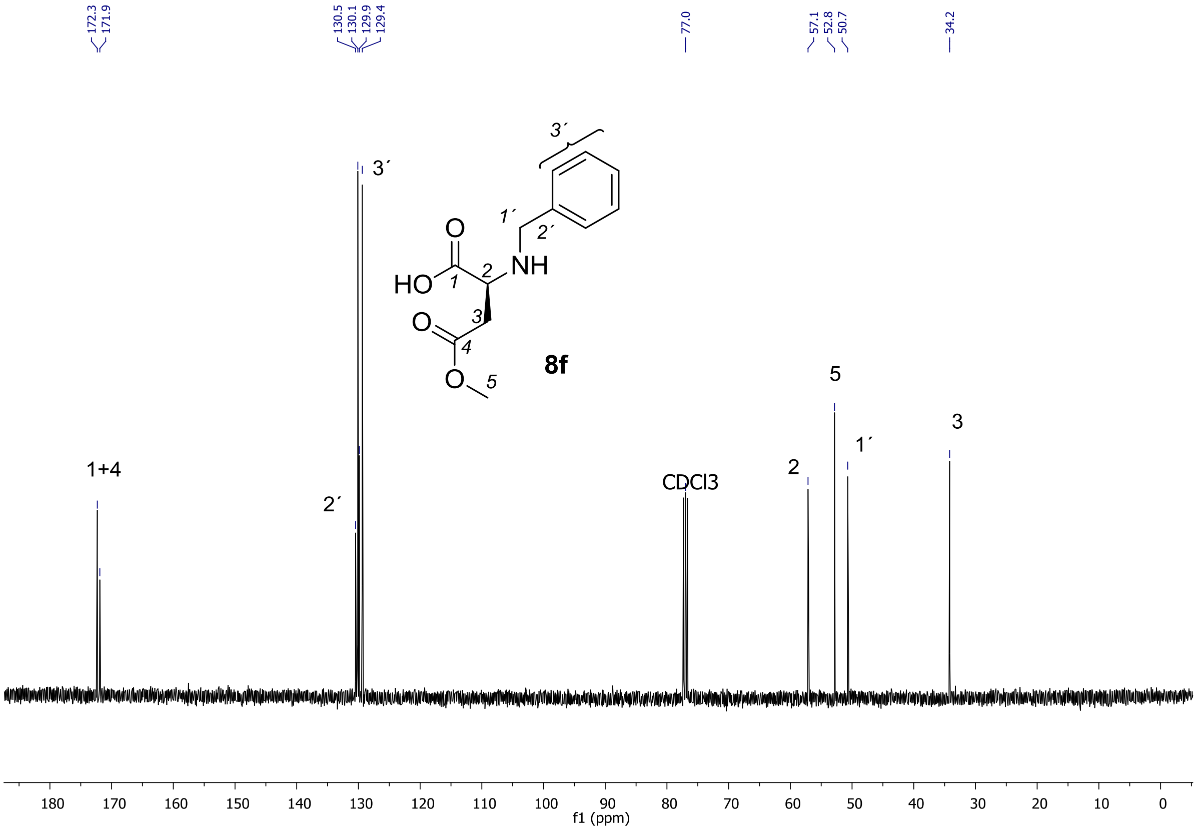

^{13}C NMR-spectra of the benzyl amine **8f** in D_2O with a capillar of $CDCl_3$ as standard (100 MHz).

3.4.3 Synthesis of (*S*)-2-(benzyl(benzyloxycarbonyl)amino)pentanedioic acid 5-methylester (2e)

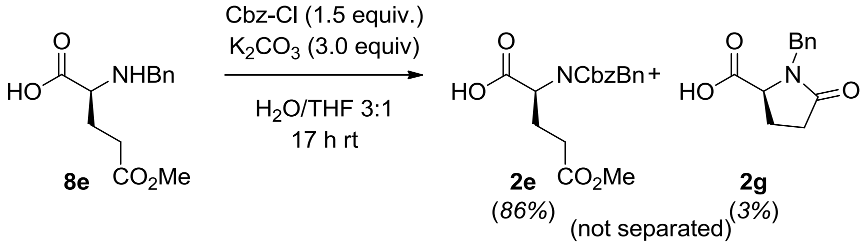

The benzyl amine **8e** (1.879 g, 7.48 mmol, 1.0 equiv) was suspended in H_2O (15 mL, [**8e**] = 0.5 mol/L) and K_2CO_3 (3.10 g, 22.4 mmol, 3.0 equiv) was added. Immediately, the mixture was cooled to 0 °C and a solution of Cbz-Cl (1.60 mL, 11.22 mmol, 1.5 equiv) in THF (5 mL, H_2O/THF 3:1) was added dropwise over 5 min. After 1 h of stirring the cooling bath was removed and the heterogeneous reaction mixture was allowed to stir for 17 h at room temperature.

Subsequently, THF was removed under reduced pressure and the residue (pH = 10, two phases) was washed with Et_2O (3 x 10 mL, addition of Et_2O to the residue results in three phases). Then the two lower, aqueous phases were cooled in an ice bath and 3 N HCl solution (aq., 9 mL) was added dropwise, until a pH of ≤ 0 was reached. During the acidification the product **2e** precipitated partly. The mixture was extracted with EtOAc (3 x 20 mL), the collected EtOAc phases were dried over $MgSO_4$ and concentrated under reduced pressure. After concentration with *c*-Hex (2 x 5 mL) to remove residual EtOAc and drying in high vacuum for 4 h under stirring and heating to 60 °C of the acid **2e** and the lactam **2g** were isolated (2.543 g) in a ratio of 25:1 according to ^{1}H NMR. Under consideration of the ratio **2e**/**2g** of 25:1 6.45 mmol (86%) of the acid **2e** and 0.26 mmol (3%) of the lactam **2g** were obtained.

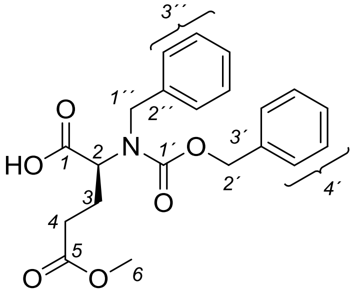

M ($C_{21}H_{23}NO_6$) = 385.410 g/mol; **^{1}H NMR** (400 MHz, $CDCl_3$, mixture of rotamers) δ [ppm] = 10.8 (s, 1H, CO_2H), 7.36-7.20 (m, 10H, H-4´, H-3´´), 5.19 (s, 2H, H-2´), 4.69-4.61 (m, 1H, H-1´´$_a$), 4.44 (d, 1H, H-1´´$_b$, *J* = 16.3 Hz), 4.28-4.18 (m, 1H, H-2), 3.99-3.90 (dd+d, 0.08H, H-2, H-1´$_b$), 3.58 (s, 3H, H-6); **^{13}C NMR** (100 MHz, $CDCl_3$, mixture of rotamers) δ [ppm] = 175.8 (C-1), 173.1 (C-5), 156.5 (C-1´), 137.1 (C-2´´), 136.0 (C-3´), 128.8/128.62/2x128.5/ 128.1/127.9/127.6 (C-4´, C-3´´), 67.9 (C-2´), 59.5/58.4 (C-2), 52.0/51.6 (C-6, C-1´´), 30.5/30.3 (C-4), 25.1/24.4 (C-3); **IR** (film) ν [cm^{-1}] = 3166 (w), 3110 (w), 3089 (w), 3064 (m), 3032 (m), 2952 (m), 2612 (w), 1958 (w), 1882 (w), 1737 (vs), 1705 (vs), 1607 (w), 1586 (w), 1497 (w), 1453 (m), 1436 (m), 1421 (m), 1366 (w), 1314 (w), 1237 (s), 1211 (s), 1176 (m), 1126 (w), 1079 (w), 1028 (w), 964 (w), 913 (w), 770 (w), 737 (w), 699 (s), 633 (s), 592 (s);

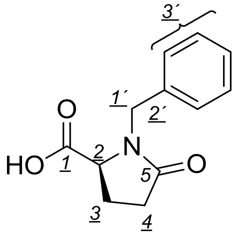

HR-MS (ESI, $[C_{21}H_{24}NO_6]^+$) calc. 386.1604 u found 386.1620 u, (ESI, $[C_{21}H_{23}NO_6Na]^+$) calc. 408.1423 u found 408.1424 u; **[α]$_D$** (c = 1.280 g/100 mL, $CHCl_3$, T = 22 °C) = -43.5.

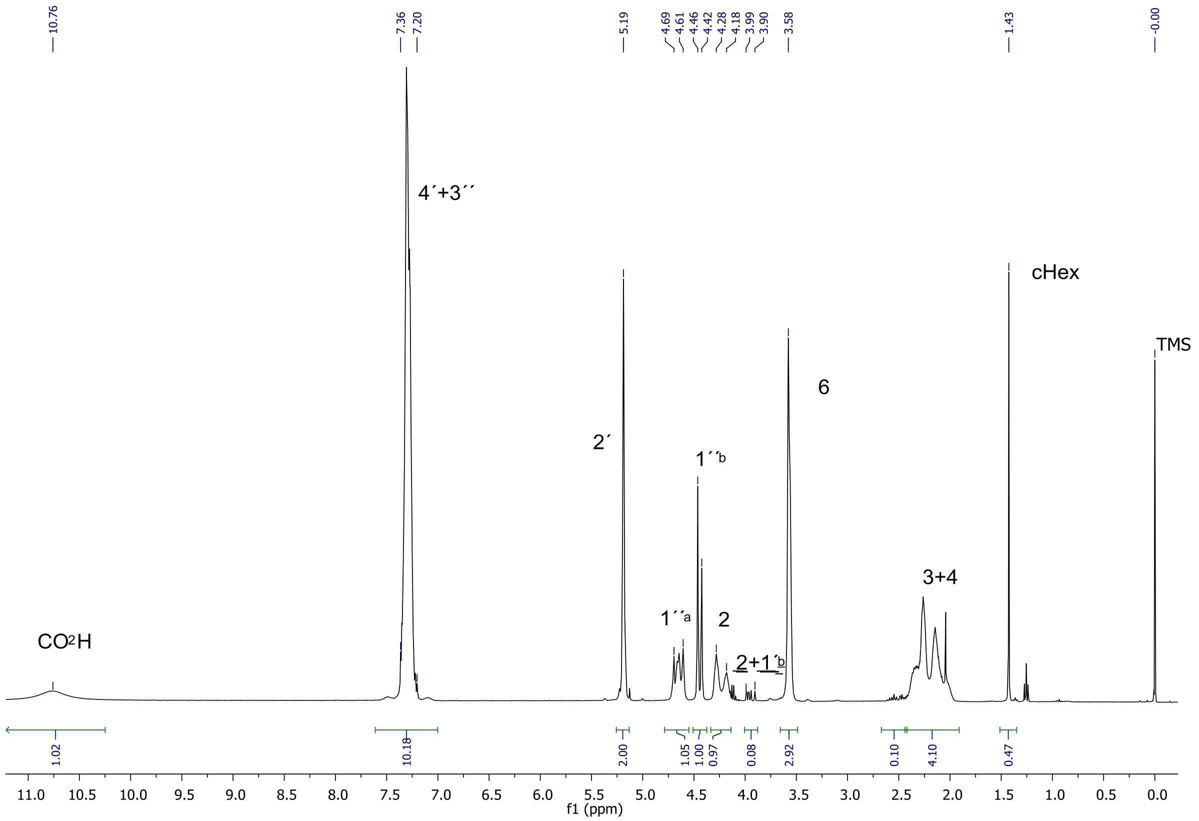

^{1}H NMR-spectra of the acid **2e**/lactam **2g** mixture in $CDCl_3$ (400 MHz).

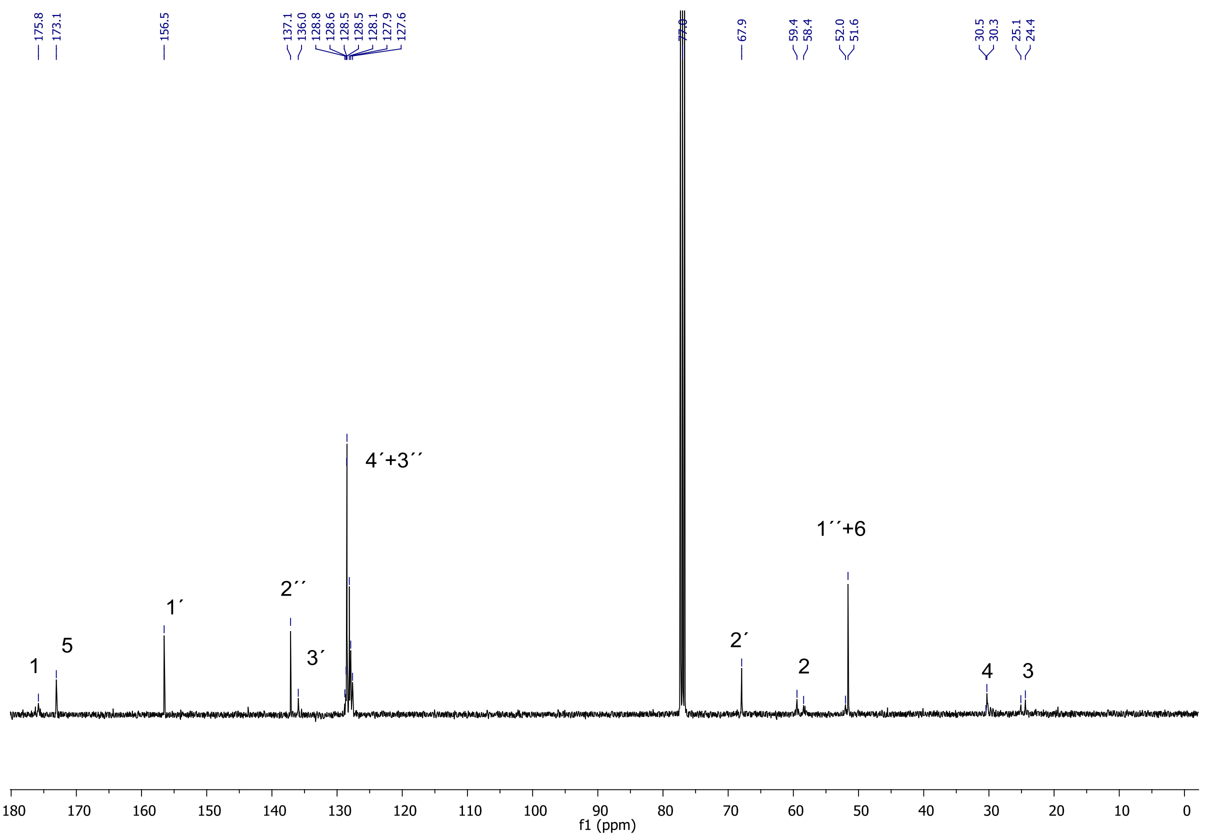

^{13}C NMR-spectra of the acid **2e**/lactam **2g** mixture in $CDCl_3$ (100 MHz)

3.4.4 Synthesis of (*S*)-2-(benzyl(benzyloxycarbonyl)amino)butanedioic acid 4-methylester (2f)

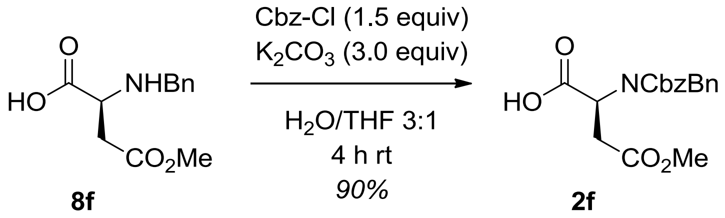

The amine **8f** (3.258 g, 13.73 mmol, 1 equiv) was suspended in water (30 mL, [**2f**] = 0.5 mol/L). Upon addition of K_2CO_3 (9.78 g, 41.2 mmol, 3.0 equiv) and stirring for 5 min at ambient temperature a clear solution was obtained. Subsequently, a solution of Cbz-Cl (4.2 mL, 20.6 mmol, 1.5 equiv) in THF (10 mL, H_2O/THF 3:1) was added dropwise under cooling to 0 °C over 15 min. After removing of the ice bath and 4 h of stirring at room temperature, THF was evaporated under reduced pressure. The residue (pH = 9–10, 2 phases) was washed with Et_2O (3 x 10 mL), cooled in an ice bath and acidified to pH ≤ 0 through the addition of 3 N HCl solution in water (12 mL). Afterwards the mixture was extracted with EtOAc (3 x 20 mL), the combined EtOAc-phases were dried over $MgSO_4$ and concentrated under reduced pressure. After drying in high vacuum for 2 h at 60 °C the carbamate **2f** (4.589 g, 12.36 mmol, 90%) was obtained as a colorless, viscous oil.

M ($C_{20}H_{21}NO_6$) = 371.384 g/mol; **^{1}H NMR** (400 MHz, $CDCl_3$, mixture of rotamers) δ [ppm] = 10.19 (s, 1H, CO_2H), 7.35-7.24 (m, 10H, H-4´, H-3´´), 5.17´(s, 2H, H-2´), 4.73-4.48 (m, 3H, H-2, H-1´´), 3.58/3.56 (2xs, 3H, H-5), 3.19/3.07 (2xdd, 1H, H-3_a, *J* = 16.6, 6.6/ 16.6, 7.0 Hz), 2.68/2.52 (2xdd, 1H, H-3_a, *J* = 16.8, 6.6/ 17.0, 6.2 Hz); **^{13}C NMR** (100 MHz, $CDCl_3$, mixture of rotamers) δ [ppm] = 175.6/175.2 (C-1), 171.3/171.0 (C-4), 156.0 (C-1´), 136.9/136.8 (C-2´´), 135.9/135.6 (C-3´), 2x128.5/128.4/128.1/128.0/127.9/ 127.7/127.6/127.0 (C-4´, C-3´´), 68.1/67.8 (C-2´), 57.2/56.2 (C-2´), 52.5/52.3 (C-2´´), 51.9 (C-5), 35.6/34.8 (C-3); **IR** (film) ν [cm^{-1}] = 3452 (br), 3167 (br), 3088 (w), 3064 (w), 3031 (m), 3006 (w), 2970 (w), 2951 (m), 2585 (w), 1958 (w), 1881 (w), 1737 (vs), 1606 (w), 1586 (w), 1497 (w), 1453 (m), 1437 (s), 1365 (s), 1230 (s), 1216 (s), 1173 (m), 1127 (w), 1079 (w), 993 (m), 913 (w), 847 (w), 770 (m), 736 (m), 698 (s), 633 (w), 593 (w), 540 (w), 527 (w); **HR-MS** (ESI, $[C_{20}H_{22}NO_6]^+$) found 372.1464 u calc. 372.1447 u, (ESI, $[C_{20}H_{21}NO_6Na]^+$) found 394.1269 u calc. 394.1267 u; **[α]$_D$** (c = 1.226 g/100 mL, $CHCl_3$, T = 22.0 °C) = -70.5.

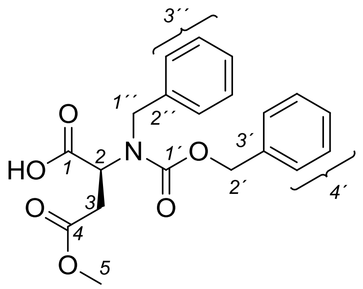

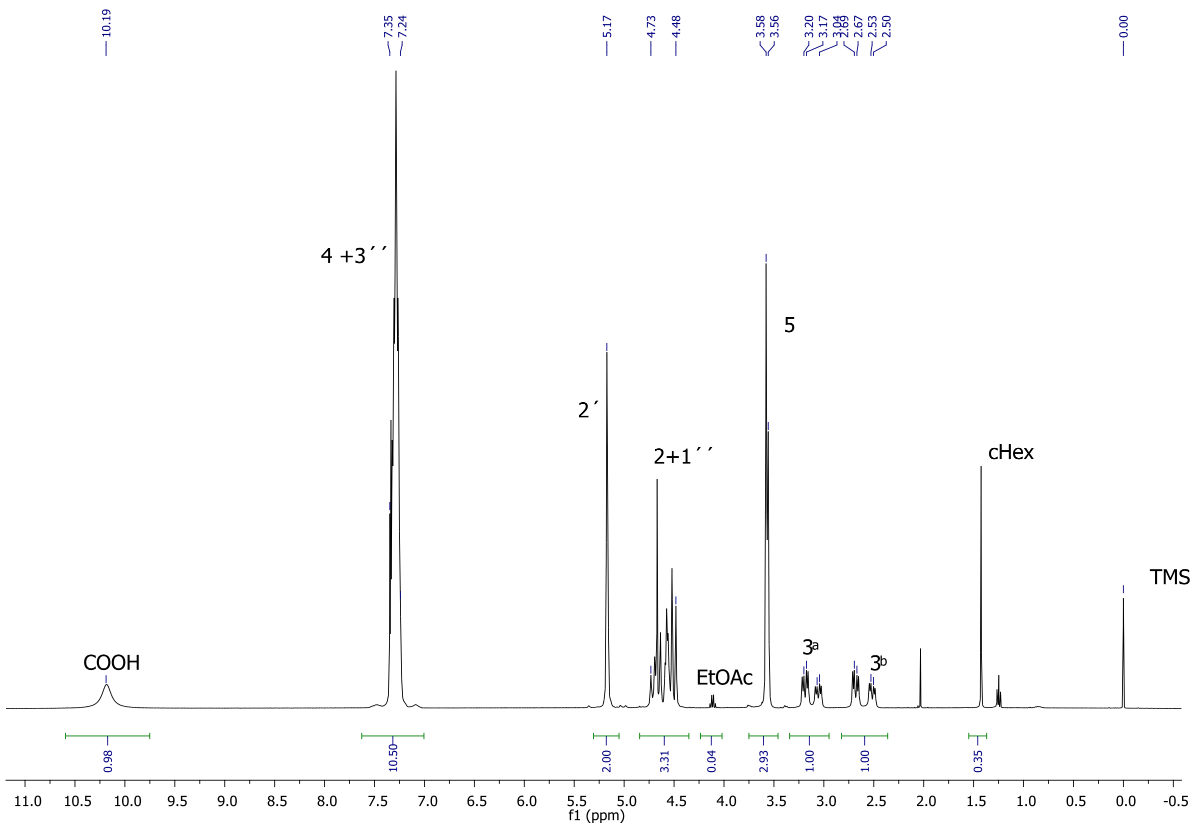

^{1}H NMR-spectra of the acid **2f** in $CDCl_3$ (400 MHz).

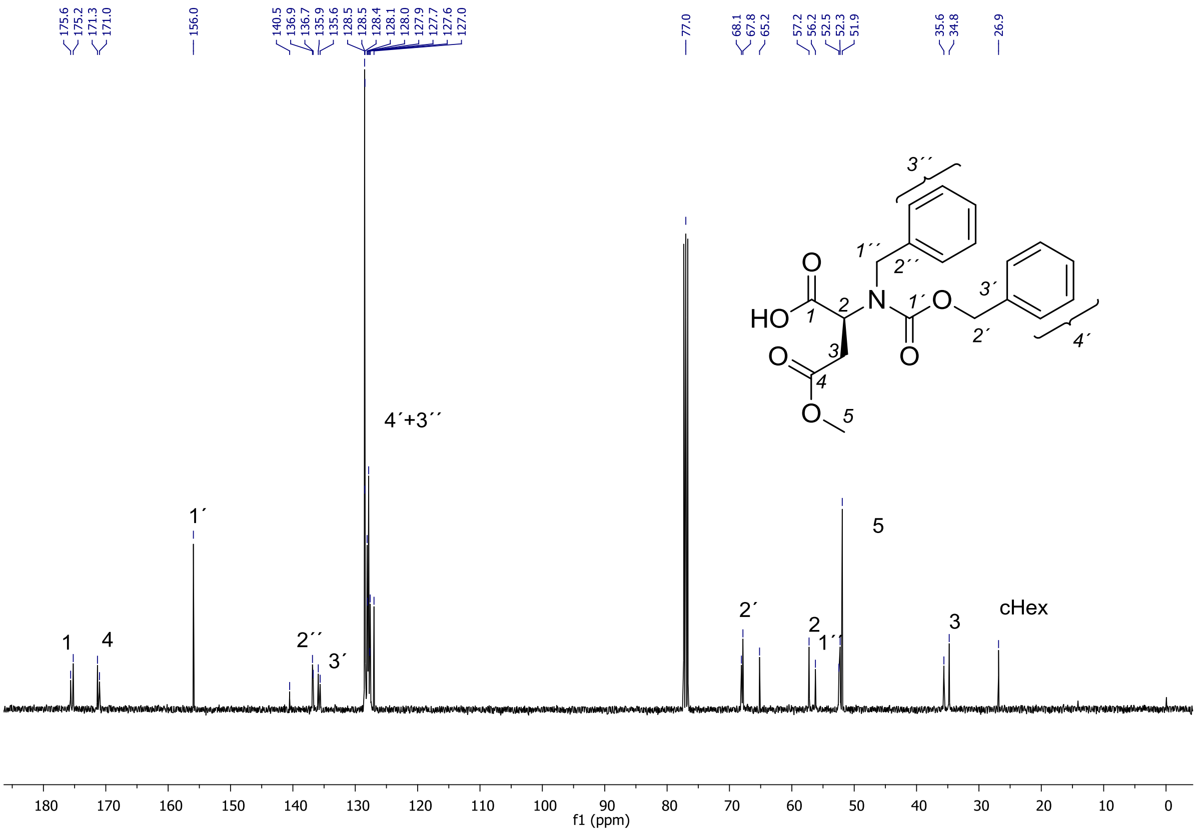

^{13}C NMR-spectra of the acid **2f** in $CDCl_3$ (100 MHz).

3.4.5 Synthesis of (*S*)-*N*-methyl-*N*-methoxy-2-(benzyl(benzyloxycarbonyl)amino) pentanedioicacid 1-amide 5-methylester (5e)

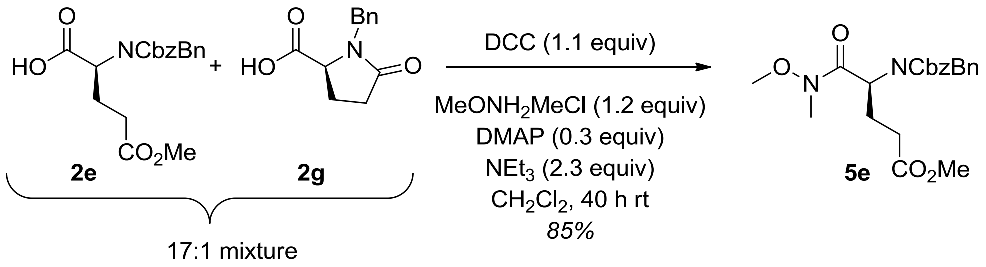

A 17:1 mixture of the acids **2e** and **2g** (4.663 g, 11.68 mmol **2e** and 0.73 mmol **2g**, 1.0 equiv) was dissolved in CH_2Cl_2 (reagent grade, 40 mL, [**2**] = 0.5 mol/L) and Et_3N (2.23 mL, 16.1 mmol, 1.3 equiv), $MeONH_2MeCl$ (1.45 g, 14.9 mmol, 1.2 equiv) and DMAP (450 mg, 3.72 mmol, 0.3 equiv) were added. Then the reaction suspension was cooled in an ice bath and DCC (2.81 g, 13.65 mmol, 1.1 equiv) was added in one portion. After 0.25 h of stirring the ice bath was removed and the mixture allowed to stir at room temperature for 40 h.

In the following the reaction suspension was concentrated under reduced pressure; the resulting solid residue was portioned between EtOAc and 1 N HCl-solution in water (both 20 mL) and stirred for 5 min at ambient temperature in order to convert residual DCC to the corresponding urea. Next the mixture was passed through a sintered funnel and the residue (urea) was washed with EtOAc (3 x

15 mL). From the combined filtrates the organic phase was washed with aqueous 1 N HCl solution, saturated $NaHCO_3$-solution in water and brine (each 20 mL). After drying over $MgSO_4$ and concentration under reduced pressure crude **5e** (6.353 g) was isolated as colorless oil. Finally, column chromatographic purification on silica gel with EtOAc/*n*-Hex 4:7, concentration under reduced pressure at the rotatory evaporator with CH_2Cl_2 (2 x 5 mL) and drying in high vacuum for 4 h under stirring delivered the amide **5e** (4.270 g, 9.97 mmol, 85%) as a colorless oil.

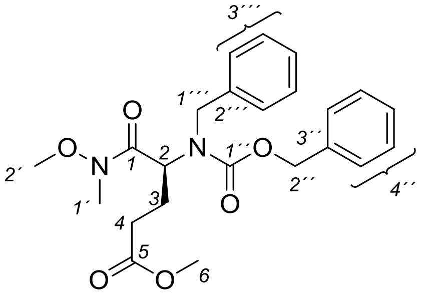

M ($C_{23}H_{28}N_2O_6$) = 428.478 g/mol; $\mathbf{r_f}$ (SiO_2, EtOAc/*n*-Hex) = 0.34 (2:3), 0.56 (1:1); **^{1}H NMR** (400 MHz, $CDCl_3$, mixture of rotamers) δ [ppm] = 7.39-7.19 (m, 10H, H-4´´, H-3´´´), 5.32-5.10 (m, 3H, H-2, H-2´), 4.61/4.60 (2xd, 1H, H-1´´´$_a$, J = 15.6/16.8 Hz), 4.49/4.43 (2xd, 1H, H-1´´´$_b$, J = 16.5/15.7 Hz), 3.65/3.63/3.54/3.29 (4xs, 6H, H-6, H-2´), 2.92/2.80 (2xs, 3H, H-1´), 2.35-1.92 (m, 4H, H-3, H-4); **^{13}C NMR** (100 MHz, $CDCl_3$, mixture of rotamers) δ [ppm] = 173.1 (C-5), 170.8/170.0 (C-1), 156.8/156.6 (C-1´´), 138.5/138.2 (C-2´´´), 136.3 (C-3´´), 128.5/128.4/128.3/128.2/128.0/127.9/127.3/127.1/126.9 (C-4´´, C-3´´´), 67.8/67.7 (C-2´´), 61.6/61.1 (C-2´), 54.1 (C-2), 51.6 (C-6), 47.4/47.1 (C-1´´´), 31.9 (C-1´), 30.3/30.0 (C-4), 24.6 (C-3); **IR** (film) ν [cm^{-1}] = 3088 (w), 3063 (w), 3031 (w), 3002 (w), 2949 (w), 1737 (s), 1699 (vs), 1666 (s), 1605 (w), 1586 (w), 1496 (w), 1450 (s), 1438 (s), 1410 (s), 1389 (w), 1366 (w), 1311 (m), 1286 (m), 1251 (s), 1211 (m), 1174 (m), 1118 (m), 1074 (w), 1029 (w), 993 (m), 950 (w), 913 (w), 823 (w), 766 (w), 740 (m), 698 (s), 632 (w), 594 (w), 558 (w); **GC-MS** (EI, 70 eV) m/z [u] = 340 (2%), 296 (10%), 91 (100%, [Bn^+]), 77 (3%, $[Ph]^+$), 65 (5%, $[Cp]^+$); **HR-MS** (ESI, $[C_{23}H_{29}N_2O_6]^+$) found 429.2043 u calc. 429.2026 u, (ESI, $[C_{23}H_{28}N_2O_6Na]^+$) found 451.1861 u calc. 451.1845 u; **$[\alpha]_D$** (c = 1.386 g/100 mL, $CHCl_3$, T = 22.0 °C) = -45.7.

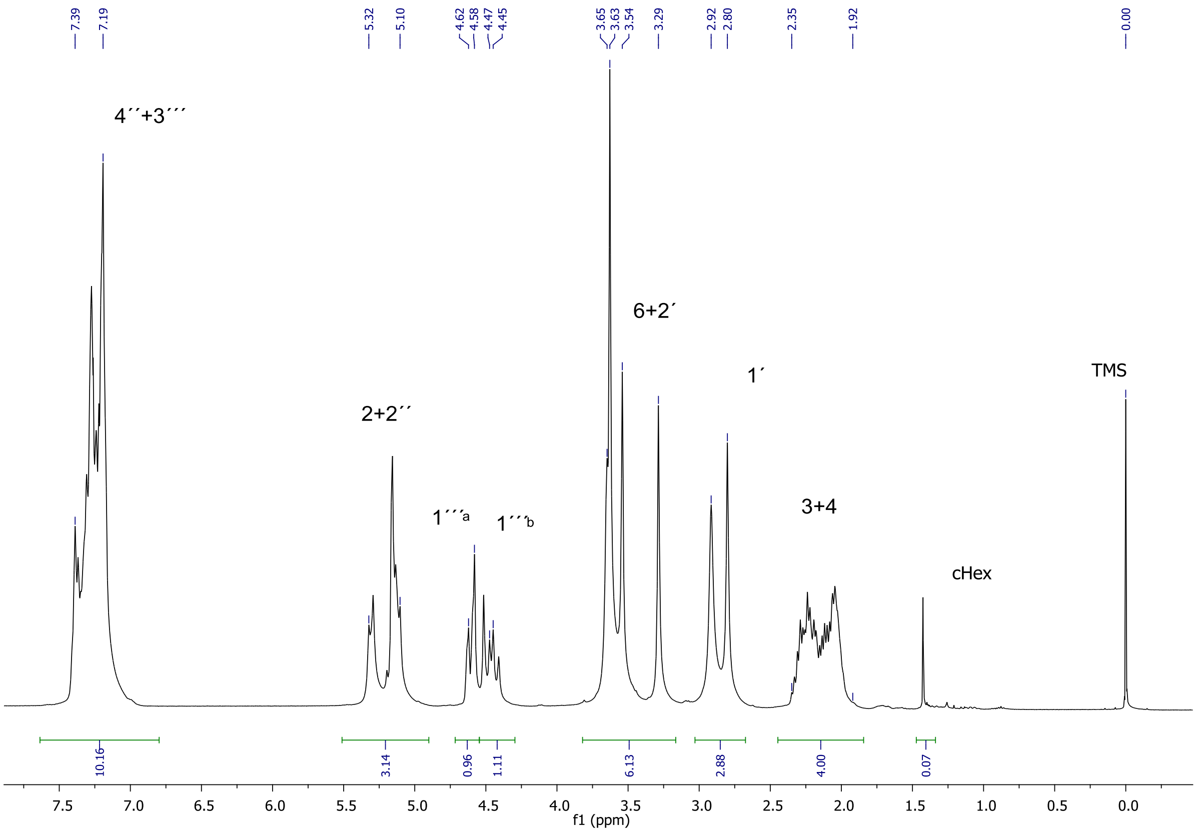

^{1}H NMR-spectra of the Weinreb amide **5e** in $CDCl_3$ (400 MHz).

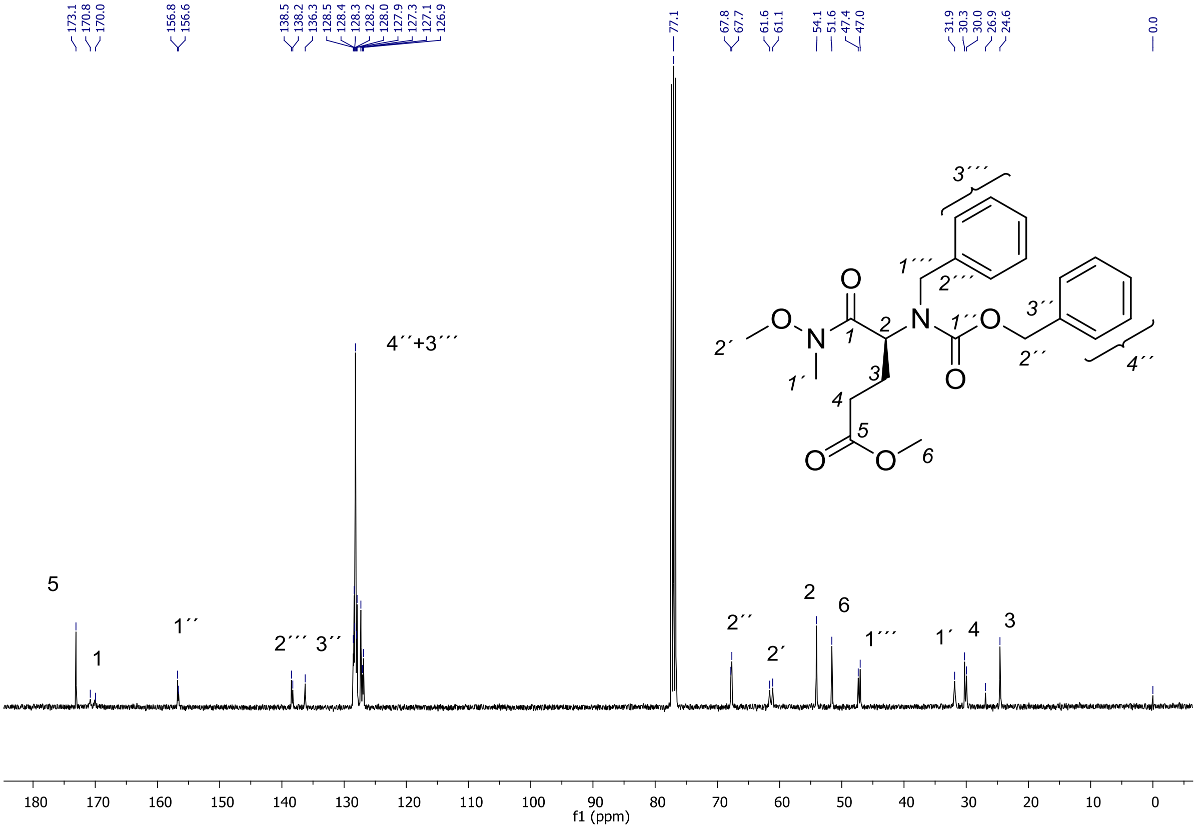

^{13}C NMR-spectra of the Weinreb amide **5e** in $CDCl_3$ (100 MHz).

3.4.6 Synthesis of (*S*)-*N*-methyl-*N*-methoxy-2-(benzyl(benzyloxycarbonyl)amino) butanedioic acid 1-amide 4-methylester (5f)

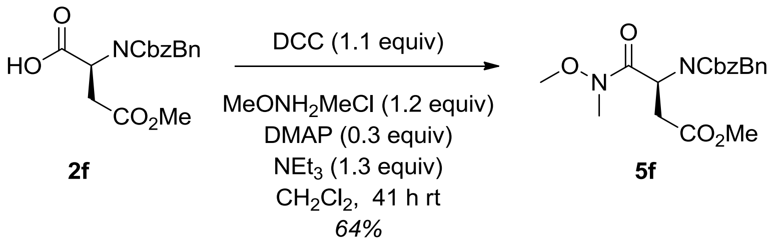

The acid **2f** (4.236 g, 11.41 mmol, 1.0 equiv) was dissolved in CH_2Cl_2 (30 mL, [**2f**] = 0.4 mol/L) and NEt_3 (2.1 mL, 14.8 mmol, 1.3 equiv), $MeONH_2MeCl$ (1.34 g, 13.69 mmol, 1.2 equiv) and DMAP (420 mg, 3.42 mmol, 0.3 equiv) were added. Then the reaction suspension was cooled in an ice bath and DCC (2.59 g, 12.6 mmol, 1.1 equiv) was added in one portion. After 0.25 h of stirring the ice bath was removed and the mixture allowed to stir at room temperature for 41 h.

In the following the reaction suspension was concentrated under reduced pressure, the resulting solid residue was diluted with EtOAc and 1 N HCl-solution in water (both 20 mL) and stirred for 5 min at ambient temperature in order to quench the excess of DCC. Then the mixture was passed through a sintered funnel and the residue (urea) was washed with EtOAc (3 x 10 mL). From the combined filtrates the organic phase was washed with aqueous 1 N HCl solution, saturated $NaHCO_3$-solution in water and brine (each 20 mL). After drying over $MgSO_4$ and concentration under reduced pressure the crude amide **5f** (5.546 g) was isolated as a colorless oil. Finally, column chromatographic purification on silica gel with EtOAc/*n*-Hex 1:2 → 4:7, concentration under reduced pressure at the rotatory evaporator with *c*-Hex (2 x 5 mL) and drying in high vacuum for 18 h under stirring delivered the amide **5f** (3.026 g, 7.30 mmol, 64%) as a colorless oil.

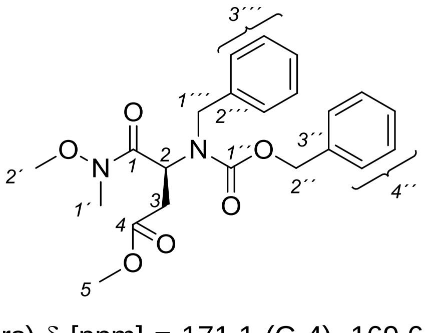

M ($C_{22}H_{26}N_2O_6$) = 414.452 g/mol; **r$_f$** (SiO_2, EtOAc/*n*-Hex) = 0.28 (4:7), 0.21 (1:2); **^{1}H NMR** (400 MHz, $CDCl_3$, mixture of rotamers) δ [ppm] = 7.40-7.15 (m, 10H, H-4´´, H-3´´´), 5.64/5.37 (2xs, 1Hm H-2), 5.31-5.18 (m, 2H, H-2´´), 4.67/4.56 (2xd, 1H, H-1´´$_a$, J = 15.4/16.4 Hz), 4.43/4.37 (2xd, 1H, H-1´´$_b$, J = 16.6/15.6 Hz), 3.70/3.58/3.36 (3xs, 3H, H-5, H-2´), 3.05 (dd, 1H, H-3$_a$, J = 16.4, 9.0 Hz), 2.91/2.83 (2xs, 3H, H-1´), 2.49/2.36 (2xdd, 1H, H-3$_b$, J = 16.6, 4.9/17.0, 3.8 Hz); **^{13}C NMR** (100 MHz, $CDCl_3$, mixture of rotamers) δ [ppm] = 171.1 (C-4), 169.6 (C-1), 156.1 (C-1´´), 137.9 (C-2´´´), 136.2 (C-3´´), 128.4/128./128.0/127.9/127.1/127.0 (C-4´´, C-3´´´), 67.7 (C-2´´), 61.5/61.0 (C-2´), 52.6/52.0 (C-2) 51.7 (C-5), 48.5/47.5 (C-1´´´), 34.3/33.9 (C-3), 31.90 (C-1´); **IR** (film) ν [cm^{-1}] = 3108 (w), 3089 (w), 3064 (w), 3032 (w), 3005 (w), 2950 (m), 2901 (w), 2852 (w), 2821 (w), 1958 (w), 1886 (w), 1736 (vs), 1702 (vs), 1667 (vs), 1606 (w), 1586 (w), 1496 (m), 1451 (s), 1437 (s), 1410 (s), 1360 (m), 1302 (m), 1252 (s), 1200 (m), 1167 (m), 1115 (m), 1074 (w), 1028

(w), 1001 (m), 947 (w), 913 (m), 873 (w), 848 (w), 827 (w), 785 (w), 767 (w), 742 (s), 698 (s), 631 (w), 595 (w), 555 (w); **HR-MS** (ESI, $[C_{22}H_{26}N_2O_6Na]^+$) found 437.1689 u calc. 437.1689 u; **[α]$_D$** (c = 1.052 g/100 mL, $CHCl_3$, T = 22.0 °C) = -73.3.

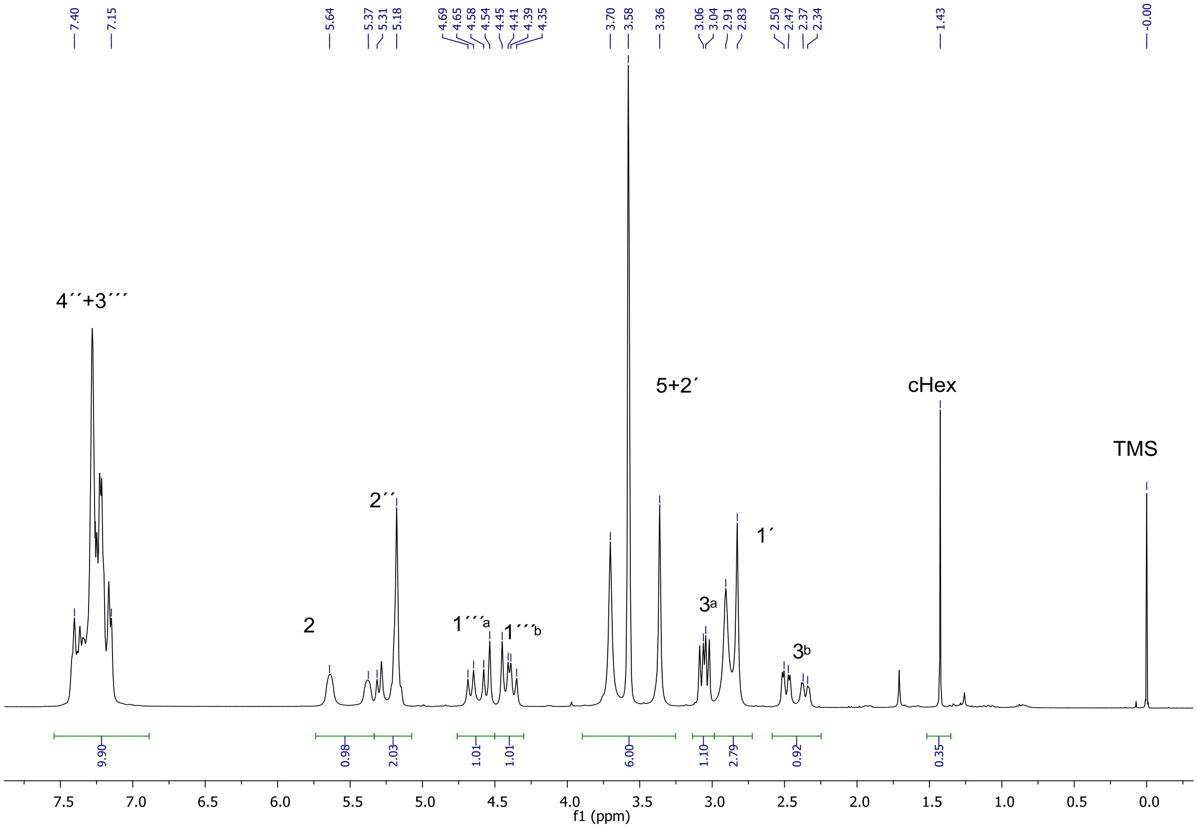

^{1}H NMR-spectra of the Weinreb amide **5f** in $CDCl_3$ (400 MHz).

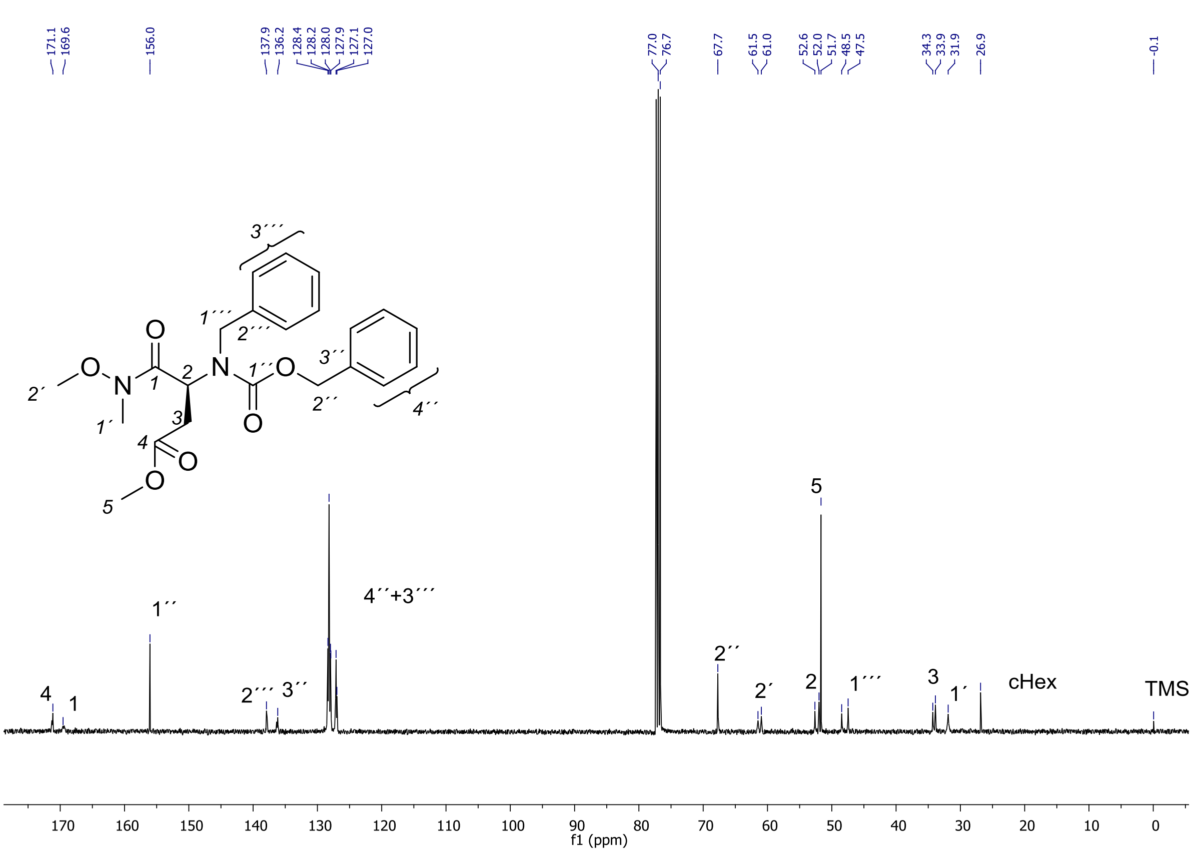

^{13}C NMR-spectra of the Weinreb amide **5e** in $CDCl_3$ (100 MHz).

3.4.7 Synthesis of (*S*)-4-(benzyl(benzyloxycarbonyl)amino)-8-hydroxy-5-oxooctanoic acid methylester (7e)

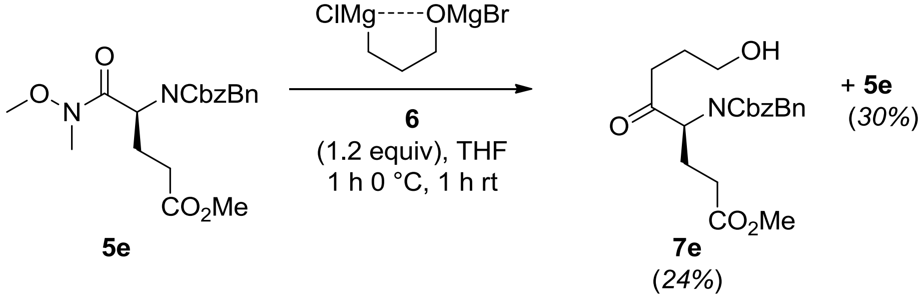

The ketone **7e** was prepared following the general procedure (chapter 3.3) for the synthesis of γ-hydroxyketones **7a–d**. After addition of the reagent **6** to the substrate **5e**, the reaction mixture was first stirred for 1 h at 0 °C and for 1 h at ambient temperature. TLC control showed still remaining starting material **5e** and additionally the formation of polar side products. Finally, the desired ketone **7e** (273 mg, 0.639 mmol) was isolated in a moderate yield of 24% as a colorless oil beside reisolated starting material **5e** (351 mg, 0.819 mmol, 30%). Grignard additions of **6** to the substrate **5e** at lower temperatures did not improve the yield further.**M** ($C_{24}H_{29}NO_6$) = 427.490 g/mol; $\mathbf{r_f}$ (SiO_2, EtOAc/*n*-Hex) = 0.30 (1:1); ^{1}**H NMR** (400 MHz, $CDCl_3$, mixture of rotamers) δ [ppm] = 7.35-7.26 (m, H-3´, H-4´´), 5.25-5.13 (m, 2H, H-2´´), 4.83/4.70 (2xd, 1H, H-1´$_a$, *J* = 15.0/15.2 Hz), 4.30, 4.24, 4.29/4.25 ((2xd, 1H, H-1´$_b$,

J = 15.2/14.7 Hz)) 4.15-4.01 (m, 1H, H-2), 3.77- 3.29 (m, 5H, H-6, H-4´´´; among at 3.63/3.63 2xs), 2.45-1.87 (m, 6H, H-4, H-1´´´, H-2´´´), 1.83-1.26 (m, 3H, H-5, OH); **^{13}C NMR** (100 MHz, $CDCl_3$, mixture of rotamers) δ [ppm] = 207.4 (C-3), 173.4 (C-3´´´), 156.2 (C-1´´), 137.3/137.11 (C-2´), 136.0/135.8 (C-3´´), 129.4/129.2/128.6/128.5/128.4/ 128.3/128.2/128.1/127.8 (C-3´/C-4´´), 107.4 (C-$\underline{3}_{acetale}$), 67.9/67.3 (C-2´´), 64.2 (C-2), 61.9/61.7 (C-6), 51.6/51.5 (C-4´´´), 51.4/50.5 (C-1´), 35.5 (C-4), 30.3/30.2 (C-2´´´), 26.3 (C-5), 23.8/23.1 (C-1´´´); **IR** (film) ν [cm^{-1}] = 3483 (br), 3088 (w), 3064 (w), 3032 (w), 2951 (m), 2884 (w), 1959 (w), 1732 (vs), 1698 (vs), 1605 (w), 1586 (w), 1540 (w), 1496 (m), 1454 (s), 1436 (s), 1365 (m), 1314 (m), 1238 (s), 1174 (m), 1122 (m), 1056 (m), 1027 (m), 913 (w), 822 (w), 768 (w), 742 (m), 701 (s), 618 (w), 592 (w); **HR-MS** (ESI, $[C_{24}H_{29}NO_6Na]^+$) found 450.1902 u calc. 450.1893 u; **[α]$_D$** (c = 1.200 g/100 mL, $CHCl_3$, T = 22.0 °C) = -71.7.

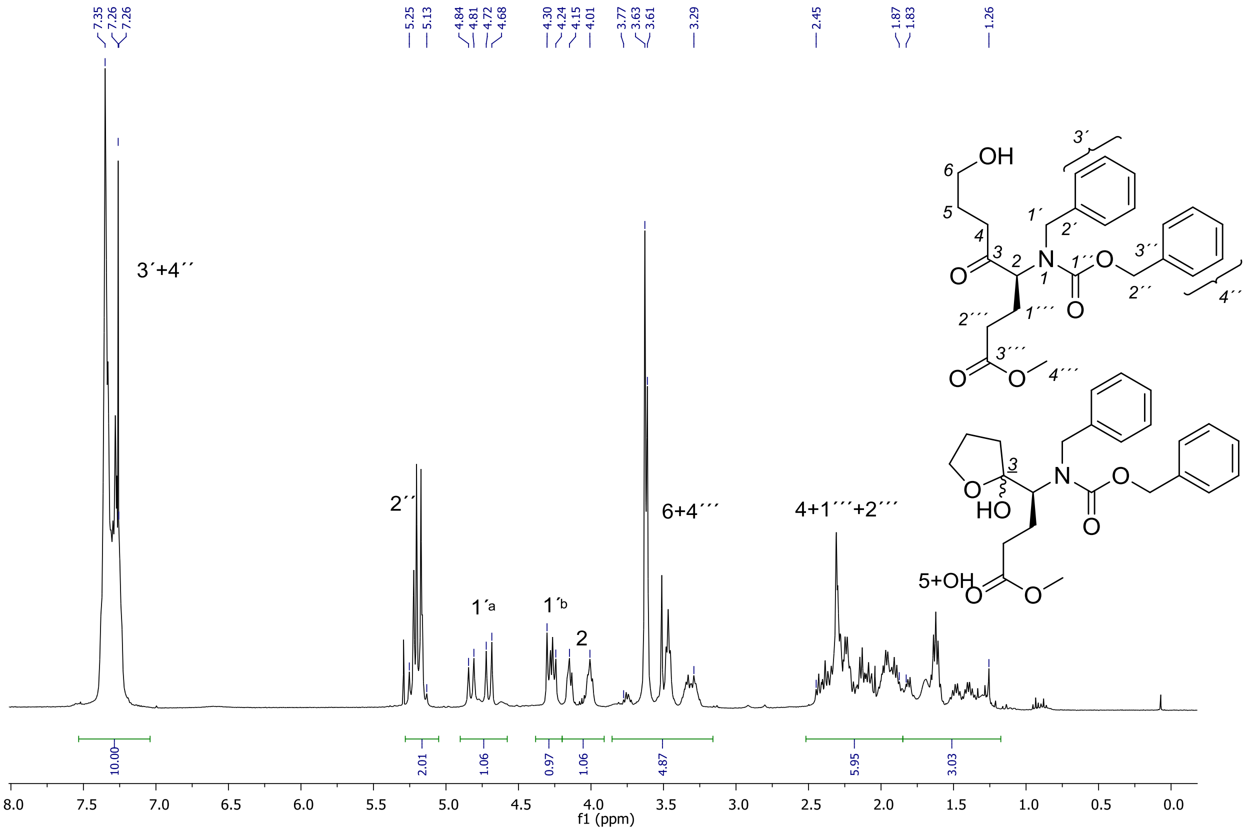

^{1}H NMR-spectra of the ketone **7e** in $CDCl_3$ (400 MHz).

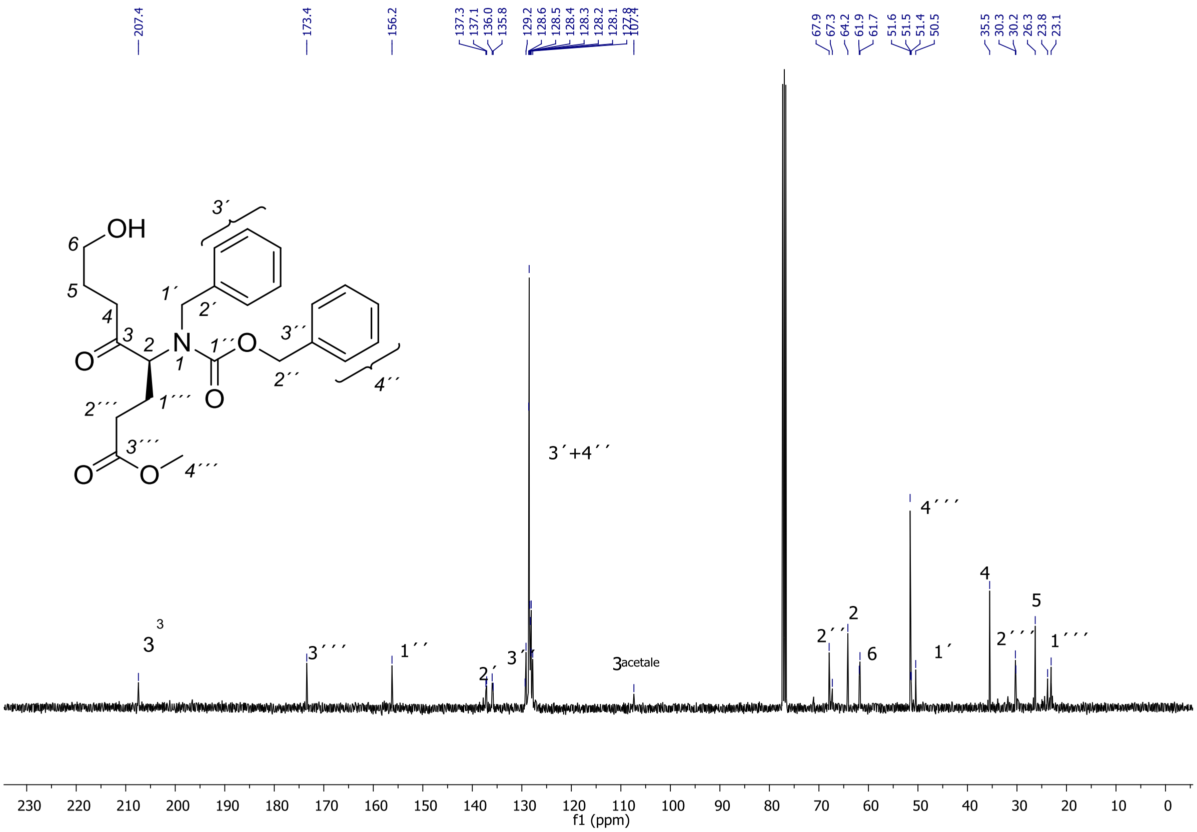

^{13}C NMR-spectra of the ketone **7e** in $CDCl_3$ (100 MHz).

3.5 Synthesis of *syn*-amino alcohols 9a–d and oxazolidinone 10a (Scheme 4)

3.5.1 General procedure for the synthesis of amino alcohols 9a–d through Selectride/Superhydride reduction

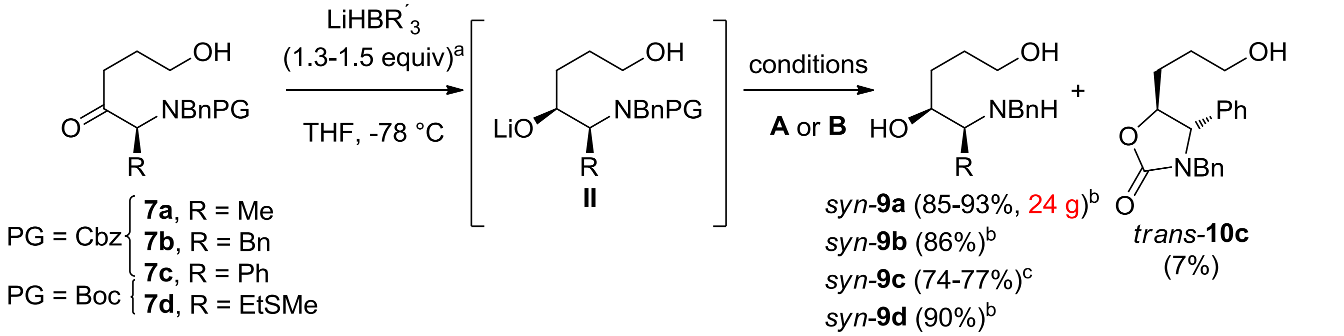

for **7a–c** conditions **A**: H_2 (1 atm), Pd/C, HCl, MeOH (THF/MeOH 1:1), rt, 3 h; for **7d** conditions **B**: HCl (aq.; 4 N HCl in THF/H_2O), rt, 6 h. a) R´= *s*Bu for **7a**, **7b** and **7d** (L-Selectride); R´= Et for **7c** (Superhydride). b) dr ≥ 19:1. c) dr = 3.7-4:1.

General Procedure for the Reduction: The ketone **7** (1 equiv) was dissolved in dry THF ([**7**] = 1 mol/L, 2 mol/L in the large scale preparation of *syn*-**9a**) under an atmosphere of argon and cooled in

a dry ice acetone bath to –78 °C. Then a 1.0 N L- or N-Selectride solution (1.3 equiv for ketones **7a,b,d**) and Superhydride solution (1.5 equiv with ketone **7c**), respectively, both in THF was added slowly, dropwise either via a dropping funnel or with the aid of a syringe pump (only a weak H_2 evolution was observed). After 0.5 h of further stirring at –78 °C micro work-up [15] and TLC control usually indicated full conversion of the starting material **7**.

Cbz-cleavage with amino ketone 7a–c (Method A)

Subsequently, concentrated HCl-solution (aq., 3.0 equiv) was added dropwise accompanied by H_2 evolution and the cooling bath was removed. During the above described addition of the reducing agent to the substrate **7**, $Pd(OAc)_2$ (0.02 equiv) was dissolved in MeOH (total amount of THF used in the reduction step/MeOH 3:2) at ambient temperature (0.5–1 h) and to the resulting brown solution was added activated charcoal (→10 wt % Pd/C). The mixture was degassed in vacuum and flushed with 1 atm of H_2 (3x) and stirred for 0.25–0.5 h at ambient temperature (under 1 atm of H_2).

Immediately after the cooling bath was removed from the (with HCl) quenched reaction mixture, the Pd/C suspension in MeOH was added to the reaction solution containing the carbamate **II**. The flask, in which Pd/C had been prepared, was rinsed with two portions of MeOH (each THF/MeOH 6:1→in total THF/MeOH 1:1), the reaction suspension was degassed in vacuum/purged with 1 atm of H_2 (6 times). Then the mixture was stirred under 1 atm of H_2 (balloon) until TLC-control indicated full conversion of the carbamate intermediate **II** (usually 2.5–3 h, in the large scale preparation of **9a** overnight). To remove Pd/C, the mixture was passed through a layer of celite (ca. 1 cm, in a sintered funnel), the residue was washed with two portions of MeOH and the solvent was removed under reduced pressure in a rotatory evaporator.

Boc-cleavage with amino ketone 7d (Method B)

At –78 °C the Boc deprotection was initiated through the dropwise addition of conc. HCl solution in water (13.6 equiv → 4 N HCl in THF/H_2O) under H_2-evolution, the cooling bath was removed and the reaction mixture was stirred at ambient temperature until TLC showed full conversion of the carbamate intermediate **II** (5–6 h). Then THF was removed under reduced pressure.

Work up of amino alcohols *syn*-9a–d

The residue was dissolved in H_2O ([*syn*-**9a–c**] = 0.5–0.7 mol/L, [*syn*-**9d**] = 0.3 mol/L) and Et_2O (H_2O/Et_2O 2:1, pH of the mixture ≤ 0–1), the aqueous phase was separated and washed with further two portions of Et_2O (each H_2O/Et_2O 2:1) to remove B*s*Bu_3/BEt_3 and residual 3-chloropropanol. Next, the H_2O-phase was cooled in an ice bath, neat NaOH (4.0 equiv (**9a–c**)/15.0 equiv (**9d**)) was added under stirring and the resulting emulsion was stirred at 0 °C, until NaOH had dissolved completely (ca. 10 min, pH ≥ 14). The resulting emulsion was extracted with three portions of CH_2Cl_2 (**9a**)/Et_2O (**9b,c,d**)

15. The reduction was followed by quickly quenching a small aliquot of the reaction mixture (ca. 50 μL) in 1 N HCl solution (aq.) and Et_2O and TLC. The quenched sample containing intermediate **II** as neutral diol was used as reference sample to monitor the reaction progress of the subsequent one-pot protecting group cleavage (**II→9a-d**) via TLC.

(H_2O/extraction solvent 2:1). Finally, the collected extraction phases were dried over $MgSO_4$, concentrated under reduced pressure, dissolved in a small amount of CH_2Cl_2 an concentrated in vacuo to remove residual Et_2O (2–3x) and dried in high vacuum under stirring and heating to 40 °C (only for substrates **9b,c** necessary; purity of ≥90% according to ^1H NMR) for several hours. Analytical data of derivatives **9a–c** was published in the Supporting Information of [2].

(2*S*,3*S*)-2-(Benzylamino)-3,6-hexandiol (*syn*-9a)

yield: 24.18 g (108.4 mmol, 85% over 2 steps from Weinreb amide **5a**, yellow oil;. yield: 1.323 g (5.76 mmol, 93% considering a content of 8 mol % of CH_2Cl_2, colorless oil).

(2*S*,3*S*)-2-(Benzylamino)-1-phenyl-3,6-hexandiol (*syn*-9b)

yield: 1.84 g (6.14 mmol, 86%, dr > 19:1, pale yellow oil).

(1*S*,2*S*)- and (1*S*,2*R*)-1-(Benzylamino)-1-phenyl-2,5-pentandiol (*syn*-9c)

yield: 1.55 g (5.44 mmol, 77%; dr = 3.7:1 *syn*/*anti*, pale yellow oil).

yield: 4.72 g (16.54 mmol, 74%, dr = 4:1 *syn*/*anti*, yellow oil).

(3*S*,4*S*)-3-(Benzylamino)-1-(methylthio)-4,7-heptandiol (*syn*-9d)

yield: 1.91 g (6.75 mmol, 90%, dr > 19:1, pale yellow oil).

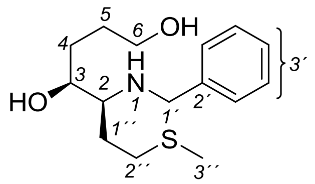

M ($C_{15}H_{25}NO_2S$) = 283.429 g/mol; **^{1}H NMR** (400 MHz, $CDCl_3$, TMS, dr > 19:1) δ [ppm] = 7.36-7.24 (m, 5H, H-3´), 3.87 (d, 1H, H-1´$_a$, J = 12.8 Hz), 3.75 (d, 1H, H-1´$_b$, J = 12.7 Hz), 3.71-3.58 (d, 2H, H-6), 3.42-3.38 (m, 1H, H-3), 2.64-2.50 (m, 3H, H-2, H-2´´), 2.11 (s, 3H; H-3´´), 1.93-1.83 (m, 1H, H-1´´$_a$), 1.77-1.68 (m, 4H, H-4$_a$, H-5, H-1´´$_b$), 1.52-1.41 (m, 1H, H-4$_b$); **^{13}C NMR** (100 MHz, $CDCl_3$) δ [ppm] = 140.3 (C-2´), 128.8/128.5/127.6 (C-3´), 71.9 (C-3), 62.7 (C-6), 60.6 (C-2), 51.5 (C-1´), 31.3 (C-4), 30.5 (C-2´´), 29.9 (C-5), 29.2 (C-1´´), 15.3 (C-2´´); **IR** (film) [cm^{-1}] = 3325 (s, br), 3080 (w), 3046 (w), 3024 (w), 2912 (s), 2853 (s), 1946 (w), 1880 (w), 1810 (w), 1707 (m), 1646 (w), 1601 (m), 1583 (w), 1493 (m), 1451 (s), 1430 (s), 1356 (w), 1317 (w), 1276 (w), 1230 (w), 1203 (w), 1170 (w), 1163 (w), 1055 (s), 1023 (m), 1006 (m), 953 (m), 913 (w), 840 (w), 738 (s), 697 (s); **GC-MS** (EI, 70 eV) m/z [u] = 283 (<1, $[M]^+$), 224 (3), 208 (1, $[M-CH_2CH_2SMe]^+$), 194 (80), 146 (15), 104 (5), 91 (100, $[Bn^+]$), 77 (5, $[Ph]^+$), 65 (15, $[Cp]^+$); **HR-MS** (ESI, $[C_{15}H_{26}NO_2S]^+$) calc. 284.1679 u found 284.1679 u, (ESI $[C_{15}H_{25}NO_2SNa]^+$) calc. 306.1498 u found 306.1499 u; **[α]$_D$** (c = 2.21 g/100 mL, $CHCl_3$, T = 20.0 °C)= -0.4.

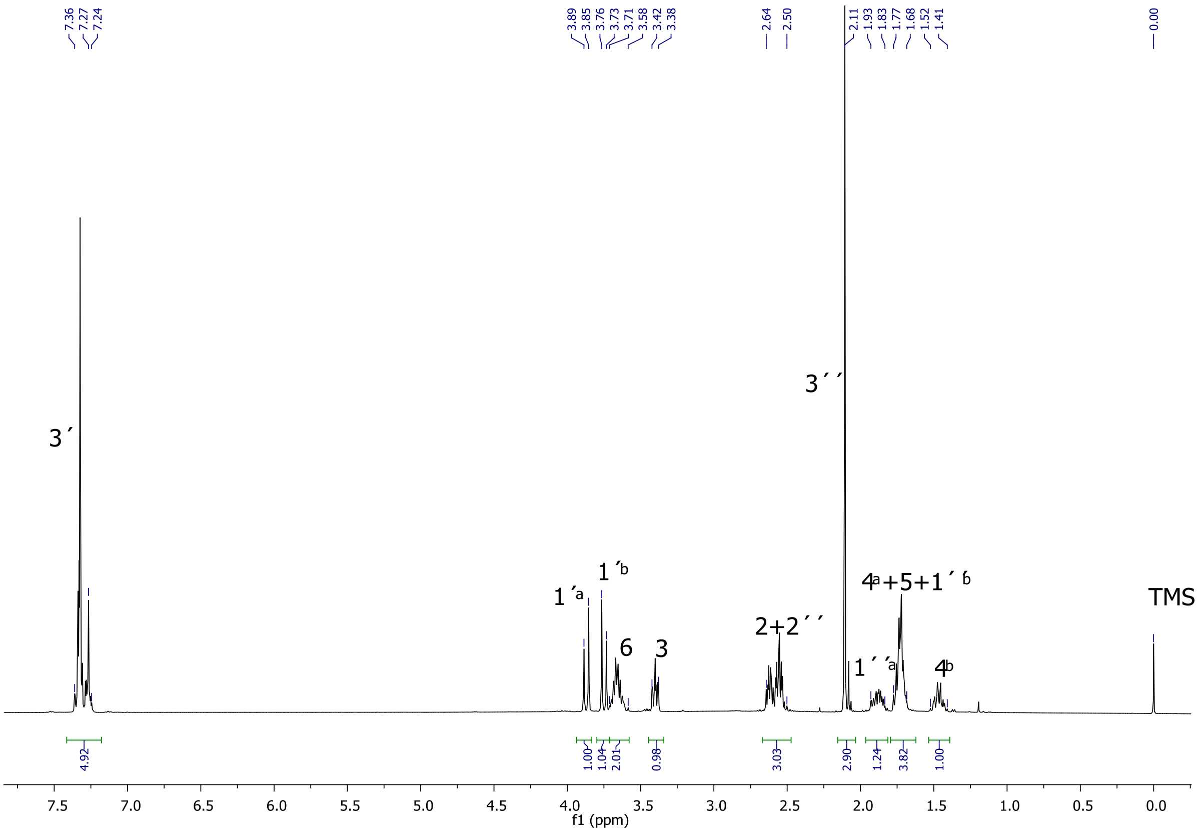

^{1}H NMR-spectra of the amino alcohol *syn*-**9d** in $CDCl_3$ (400 MHz).

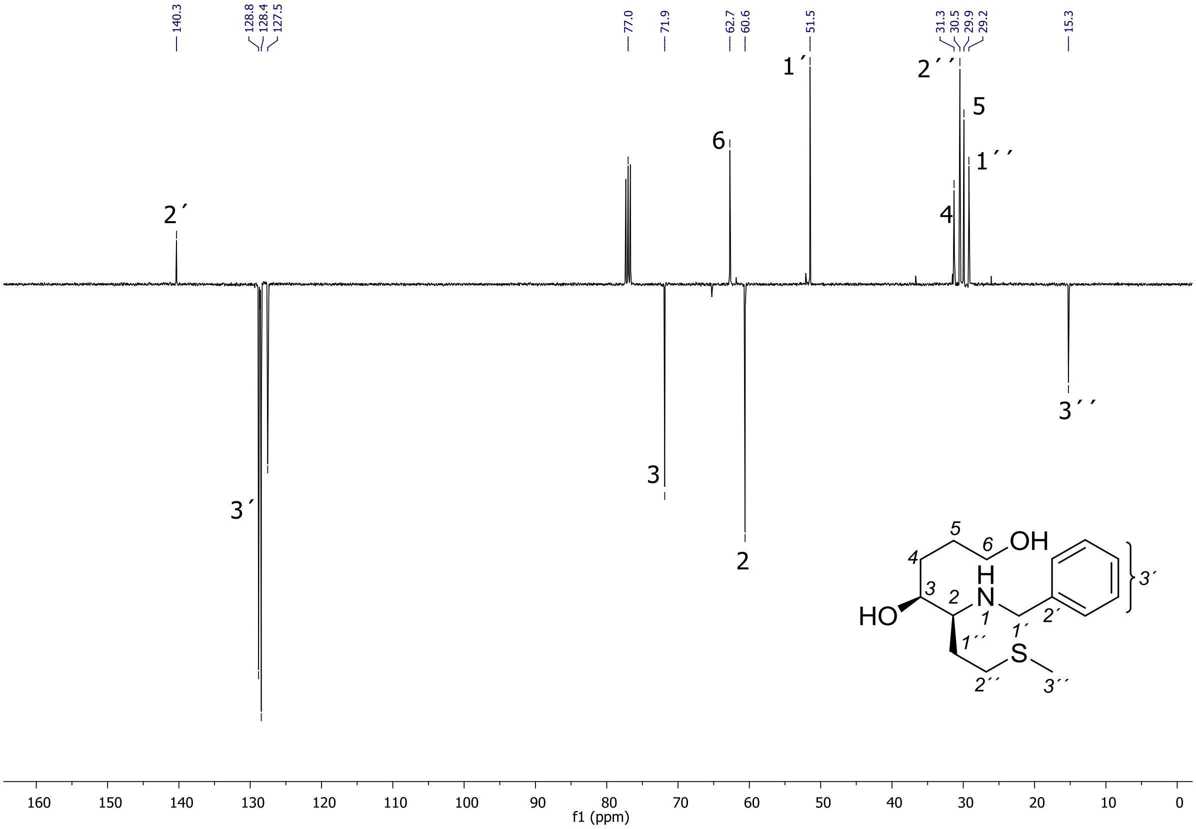

APT-NMR-spectra of the amino alcohol *syn*-**9d** in $CDCl_3$ (100 MHz).

3.5.2 Synthesis of (4*S*,5*S*)-1-benzyl-(4-hydroxypropyl)-5-phenyl-2-oxazolidinone (10a)

The experimental procedure and analytical data of derivative **10a** can be found in the Supporting Informations of [2].

3.5.3 Synthesis of (4*S*,5*S*)-1-benzyl-(4-hydroxypropyl)-5-phenyl-2-oxazolidinone (10c)

The oxazolidinone **10c** was obtained as a side product in the reduction/Cbz-cleavage of ketone **7c** according to the general procedure for the synthesis of amino alcohols *syn*-**10c**. Here drying of the combined Et_2O-phases over $MgSO_4$, concentration under reduced pressure, chromatographic purification on silica gel with EtOAc/*c*-Hex 1:1, concentration with chloroform (2 x 3 mL) and drying in high vacuum for 11 h delivered the cyclic carbamate **10c** (514 mg, 1,61 mmol, dr > 19:1) as a pale yellow oil in 7% yield. Analytical data of **10c** are given in the Supporting Information of [2].

3.5.4 General procedure for the synthesis of amino alcohols 9a–c through $NaBH_4$-reduction

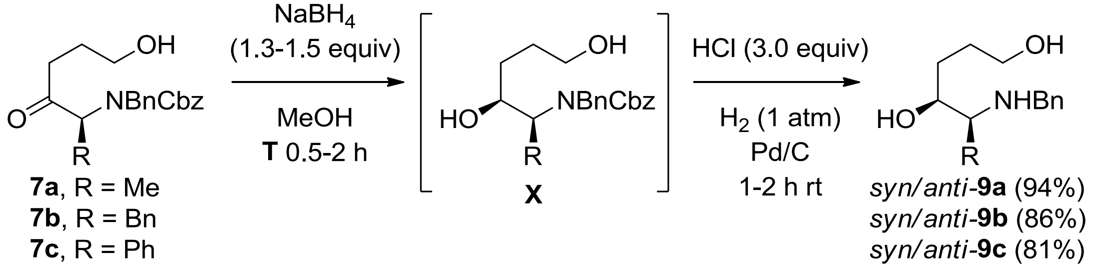

The ketone **7** (1.0 equiv) was dissolved in MeOH ([**7**] = 0.3–0.5 mol/L) and cooled to the indicated temperature **T**. Then $NaBH_4$ (1.5 equiv) was added portionwise and the reaction mixture was stirred until micro work up (1 N HCl solution (aq.)/EtOAc) and TLC control indicated full conversion (0.5–2 h) [16]. Subsequently, concentrated aqueous HCl solution (3.0 equiv) was added dropwise (thereby NaCl precipitated) the cooling bath was removed (H_2-evolution). Before addition of commercial 10 wt % Pd/C (0.02 equiv), the reaction mixture was degassed in vacuum/flushed with air (3x) to avoid explosive reaction of hydrogen with oxygen catalyzed by Pd. Next, the reaction suspension was degassed in vacuum/purged with 1 atm of H_2 (3 times) and stirred under 1 atm of H_2 (balloon) until TLC-control indicated full conversion of the carbamate intermediate **II** (usually 1–2 h).

Next the mixture was passed through a thin layer of celite, the celite was washed with two portions of MeOH and the collected filtrates were concentrated under reduced pressure. The residue was diluted with water (2 mL/1 mmol **7**, pH ≤ 0) and washed with Et_2O (3x, H_2O/Et_2O 2:1) [17]. Thereon the

16. Through $NaBH_4$ reduction –78 °C the dr of **9a** was improved to >15:1 (6-12 h of reaction time). In contrast to the reduction with L-Selectride here the minor diastereomer was still visible in NMR. $NaBH_4$ reductions of ketones **7b** and **7c** were hard to force to completion at these low temperatures showing the increased sterically shielding of the carbonyl function through the Bn- and Ph-substituent, respectively.

17. Instead of a extraction funnel a 20 mL syringe was used for the work up.

aqueous phase was treated with NaOH (4.0 equiv) under cooling to 0 °C and the resulting emulsion (pH = 13– ≥14) was extracted with CH_2Cl_2 (**9a**)/ Et_2O (**9b–c**) (H_2O/organic solvent 2:1). The combined extraction phases were dried over $MgSO_4$, concentrated under reduced pressure, dissolved in a small amount of CH_2Cl_2 an concentrated in vacuo to remove residual Et_2O (2–3x) and dried in high vacuum under stirring for several hours.

(2*S*,3*S*)- and (2*S,*3*R*)-2-(Benzylamino)-3,6-hexandiol (*syn*/*anti*-9a)

yield: 343 mg (1.54 mmol, 94%, *dr* = 11:1 *syn/anti*, pale yellow oil). T = -40 °C.

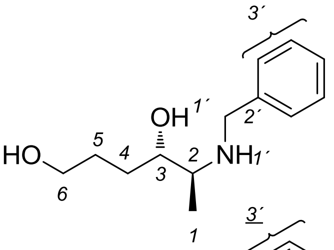

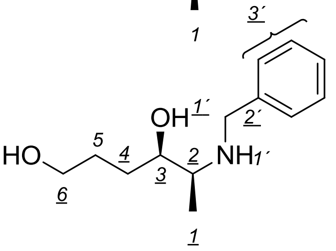

M ($C_{13}H_{21}NO_2$) = 223.311 g/mol; **r$_f$** (SiO_2, EtOAc) = 0.00; **^{1}H NMR** (400 MHz, $CDCl_3$, *dr* = 11:1 *syn/anti*) δ [ppm] = 7.35-7.17 (m, 5H, H-3´), 3.92 (d, 1H, H-1´$_a$, *J* = 13.1 Hz), 3.70-3.59 (m, 3H, H-6, H-1´$_b$; beneath at 3.69 ppm, d, H-1´$_b$, *J* = 13.1 Hz), 3.23 (td, 1H, H-3, *J* = 8.5, 2.7 Hz), 2.99 (s, 2H, NH, OH),[18] 2.74 (q, 1H, H-$\underline{2}$ of *anti* diastereomer), 2.54-2.47 (m, 1H, H-2), 1.79-1.65 (m, 3H, H-4$_a$, H-5), 1.46-1.36 (m, 1H, H-4$_b$), 1.12 (d, 3H, H-1, *J* = 6.4 Hz, *dr* = 11:1), 1.02 (d, 3H, H-$\underline{1}$ of *anti* diastereomer, *J* = 6.6 Hz); **^{13}C NMR** (100 MHz, $CDCl_3$) δ [ppm] = 139.9 (C-2´), 128.5/128.1/127.2 (C-3´), 74.5 (C-3), 71.0 (C-$\underline{3}$), 62.8 (C-5), 57.6 (C-2), 56.5 (C-$\underline{2}$), 51.3 (C-1´), 30.9 (C-4), 30.4/29.8 (C-$\underline{4}$/C-$\underline{5}$), 29.3 (C-5), 16.5 (C-1), 14.2 (C-$\underline{1}$);

18. The third heteroatomic proton is missing.

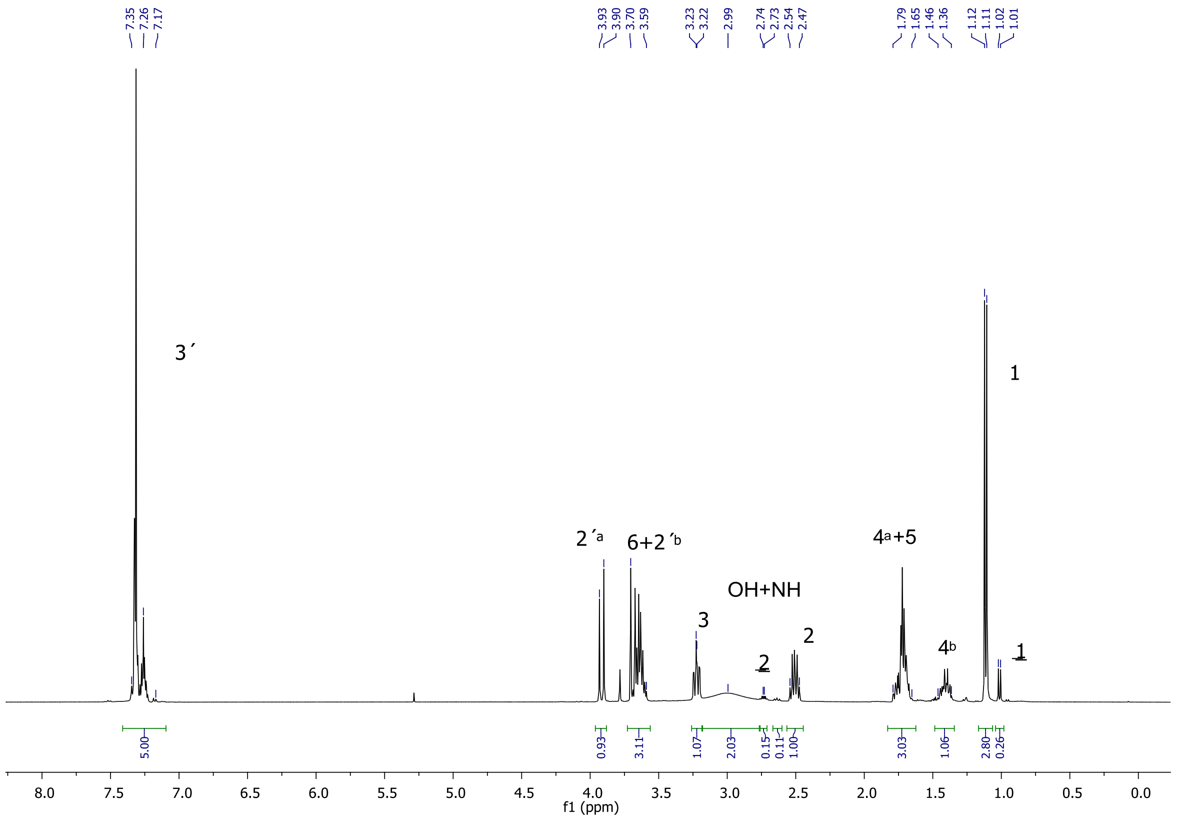

^{1}H NMR-spectra of a 11:1 mixture of the *syn*- and *anti*-epimer of amino alcohol **9a** (400 MHz).

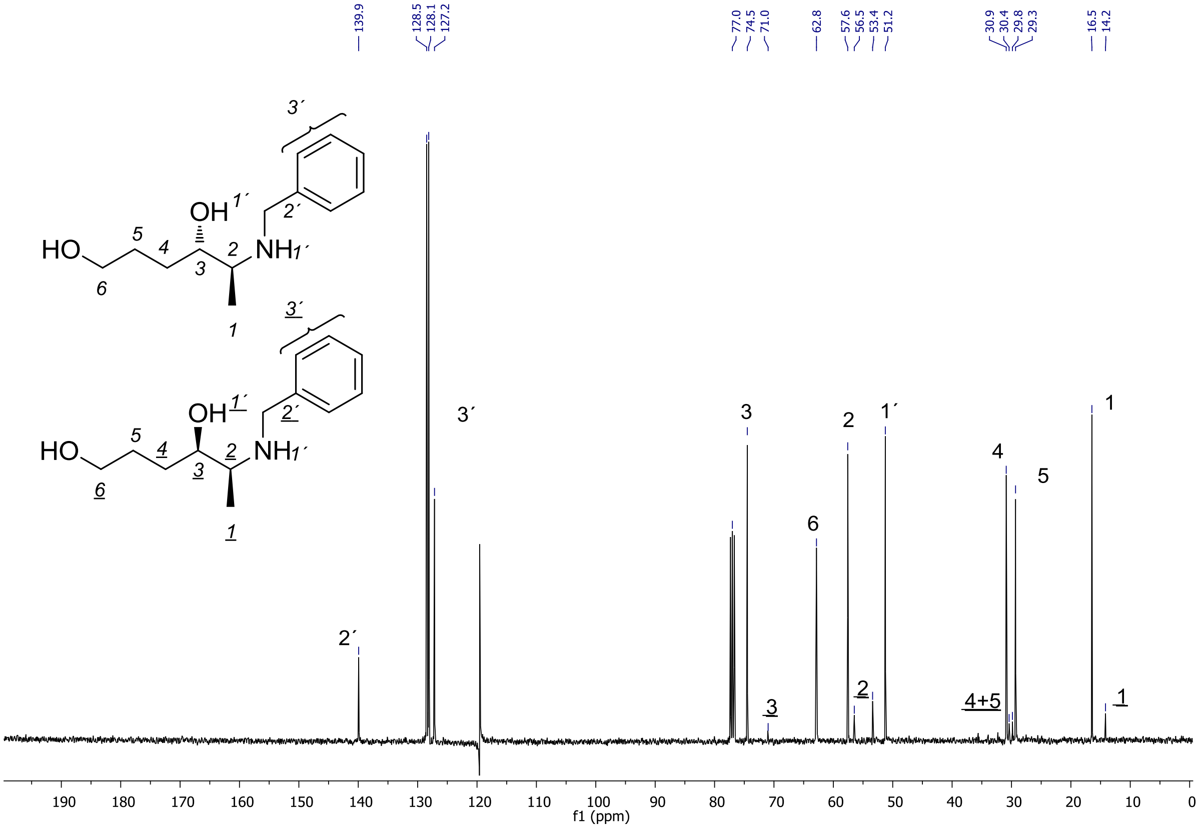

^{13}C NMR-spectra of a 11:1 mixture of the *syn*- and *anti*-epimer of amino alcohol **9a** (100 MHz).

(2*S*,3*S*) and (2*S*,3*R*)-2-(Benzylamino)-1-phenyl-3,6-hexandiol (*syn*/*anti*-9b)

Yield: 549 mg (→ 1.61 mmol, 86% considering 50 mol % residual CH_2Cl_2 according to ^{1}H NMR, colorless oil, dr = 11:1 *syn/anti* determined after subsequent cyclodehydration (I_2/PPh_3) to piperidine **11b**). T = 0 °C.

(1*S*,2*S*)- and (1*S*,2*R*)-1-(Benzylamino)-1-phenyl-2,5-pentandiol (*syn*/*anti*-9c)

Yield: 75 mg (→0.255 mmol, 81% accounting 9 mol % of residual CH_2Cl_2 according to ^{1}H NMR, dr = 1.3:1 *syn*/*anti*). T = 0 °C.

3.6 Synthesis of piperidinols *cis*-11a–d (Table 2)

3.6.1 General procedure for the synthesis of piperidinols *cis*-11a–d via phosphite mediated cyclodehydration and analytical data of *cis*-11d (Table 2)

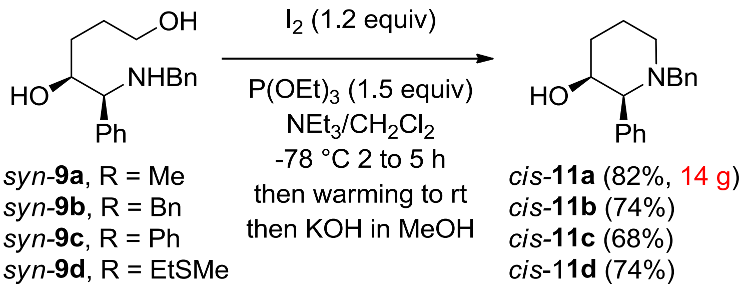

Under an atmosphere of argon the amino alcohol *syn*-**9** (1.0 equiv) was dissolved in dry CH_2Cl_2 and reagent grade Et_3N (2:1 CH_2Cl_2/NEt_3 (6.0 equiv) for **9a**,**b**,**d** or 1.3:1 CH_2Cl_2/NEt_3 (10.0 equiv) for **9c**), cooled to –78 °C and treated with triethyl phosphite (1.5 equiv). Then iodine (1.2 equiv) was added in one portion and the mixture was stirred at –78 °C [19] until iodine dissolved completely (2–5 h). During this process a colorless precipitate formed (Et_3NHCl) increasing the viscosity of the reaction mixture. After entire dissolution of iodine (I_2+$P(OEt)_3$ → $I{-}P(OEt)_3^+$ I^-) the reaction progress was monitored through micro work up with 1 N NaOH solution in water and Et_2O and TLC. With the alanine and phenylglycine derived amino alcohols **9a** and **9c** full conversion was usually achieved directly after complete reaction of iodine at –78 °C; the reaction mixtures of amino alcohols **9b** and **9d** were allowed to slowly warm to room temperature overnight (remaining in the cooling bath) to ensure quantitative conversion.

To quench the reaction, 4 N KOH solution in MeOH was added dropwise (10.0 equiv, either at −78 °C for **11a** and **11c** or under cooling to 0 °C **11b** and **11d**, the cooling bath was removed and the mixture was concentrated under reduced pressure. Then the residue was again diluted with MeOH (**9**/MeOH 1 mmol/2.5 mL), $NaBH_4$ (< 0.05 equiv) was added to remove colored impurities (optional) and the mixture stirred for 1 h at ambient temperature (whereby a color change from orange to yellow was

19. Neither slow addition of iodine as a solution in THF via syringe pump, nor slow addition of $P(OEt)_3$ to a suspension of iodine in the reaction solution lead to an improvement of the yield.

observable). In the following the solvent was removed in vacuo, the residue was dissolved in water ([**11**] = 0.5 mol/L) and Et_2O (H_2O/Et_2O 2:1), the aqueous phase was separated and extracted with two further portions of Et_2O (H_2O/Et_2O 2:1). The combined organic phases were dried over $MgSO_4$ and concentrated under reduced pressure to give crude **11** (90–95% of the theoretical yield. ^1H NMR showed no trace of triethyl phosphate) [20].

Next, the residue was purified via column chromatographic on silica gel (crude **11**/SiO_2 1:20 (**11a,b,d**)–1:50 (**11c**)) with iPrOH/*c*-Hex/Et_3N, whereby the crude piperidinol was dissolved in the eluent. Finally, the piperidinol **11** was dissolved in a small portion of CH_2Cl_2 and concentrated in vacuo to remove remaining Et_3N (x2) and dried in high vacuum under stirring and heating to 40 °C for several hours.

After cyclodehydrations of mixtures of epimers of the amino alcohols **9a–c** (e.g. obtained through $NaBH_4$ reduction and Cbz-cleavage leading to **9a** and **9b** or Superhydride reduction/Cbz-cleavage giving **9c**) during chromatographic purification the early product containing fractions were enriched with the less polar *cis*-epimer. Nevertheless, with approximately 4:1 *cis/trans*-mixtures of piperidinols **11c** separation was much more easily achieved after transformation to the Boc-carbamate **16c**. Analytical data and HPLC chromatograms of derivatives **11a–c** are present in the Supporting Information of [2].

(2*S*,3*S*)-1-Benzyl-2-methyl-3-piperidinol (*cis*-11a)

yield: 13.89 g (67.8 mmol, 82%, dr > 19:1, ee ≥ 99%, pale yellow solid), chromatographic purification with iPrOH/*c*-Hex/NEt_3 2.5:100:0.8 → 2.5:100:1.2.

(2*S*,3*S*)-1,2-Dibenzyl-3-piperidinol (*cis*-11b)

yield: 1.21 g (4.30 mmol, 74%, dr > 19:1, ee ≥ 99%, yellow oil) purified with iPrOH/*c*-Hex/NEt_3 1:100:3.

(2*S*,3*S*) and (*2S,3R*)-1-Benzyl-2-phenyl-3-piperidinol (*cis*-11c)

123 mg (→0.45 mmol, 68% considering 7 mol % of residual CH_2Cl_2, dr = 5.3:1 (dr of starting material = 4:1), ee = 90%, pale yellow oil), chromatographic purification with iPrOH/*c*-Hex/Et_3N 0.25:100:3→1:100:3.

(2*S*,3*S*)-1-Benzyl-2-(2-methylthioethyl)-3-piperidinol (*cis*-11d)

yield: 1.17 g (4.40 mmol, 75%, dr > 19:1, ee ≥ 99%, yellow oil) iPrOH/*c*-Hex/NEt_3 0.5:100:3 → 2:100:3.

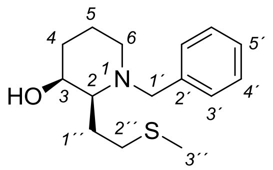

M ($C_{15}H_{23}NOS$) = 265.414 g/mol; r_f (SiO_2) = 0.58 (EtOAc), 0.35 (iPrOH/*c*-Hex 1:6), 0.70 (iPrOH/*c*-Hex 1:3); ^1H NMR (400 MHz, $CDCl_3$, TMS) δ [ppm] = 7.33-7.28 (m, 4H, H-3´, H-4´), 7.26-7.21 (m, 2H, H-5´), 3.97 (d, 1H, H-1´$_b$, J = 14.3 Hz), 3.84 (app. s, 1H, H-3), 3.26 (d, 1H, H-1´$_b$, J = 14.0 Hz), 2.72-2.68 (m, 1H, H-6$_a$), 2.66-2.55 (m, 3H, H-2´´, OH), 2.53-2.50 (m, 1H, H-2), 2.11 (s, 3H, H-3´´), 2.09-1.96 (m, 2H, H-6$_b$, H-1´´$_a$), 1.94-1.84 (m, 1H; H-1´´$_b$), 1.83-1.76 (m, 1H, H-4$_a$), 1.73-1.63 (m, 1H, H-5$_b$), 1.54-1.49 (m,

20. As $OP(OEt)_3$ was difficult to remove chromatographically, in cases where the phosphate remained after the work up procedure, the crude mixture was again treated with KOH solution and concentrated.

1H, H-4b), 1.47-1.40 (m, 1H, H-5b); ^{13}C NMR (100 MHz, $CDCl_3$, TMS) δ [ppm] = 139.5 (C-2´), 128.5/128.2 (C-3´, C-4´), 126.8 (C-5´), 67.1 (C-2), 64.2 (C-3), 57.3 (C-1´), 51.2 (C-6), 31.8 (C-2´´), 30.9 (C-4), 27.7 (C-1´´), 20.6 (C-5), 15.5 (C-3´´); IR (film) ν [cm^{-1}] = 3423 (br, s), 3080 (w), 3053 (w), 3023 (w), 2953 (s), 2853 (m), 2800 (m), 2713 (w), 1950 (w), 1873 (w), 1810 (w), 1763 (w), 1693 (w), 1603 (w), 1583 (w), 1492 (m), 1450 (s), 1430 (s), 1386 (m), 1365 (m), 1316 (w), 1252 (m), 1226 (w), 1203 (w), 1183 (w), 1163 (w), 1126 (m), 1099 (m), 1071 (s), 1027 (s), 973 (m), 916 (w), 883 (w), 840 (w), 807 (w), 733 (s), 698 (s); GC-MS (EI, 70 eV) m/z [u] = 265 (2, $[M]^+$), 218 (5, $[M\text{-}SMe]^+$), 190 (100, $[M\text{-}CH_2CH_2SMe]^+$), 174 (5, $[M\text{-}Bn]^+$), 160 (3), 146 (3), 91 (80, $[Bn]^+$), 65 (5, $[Cp]^+$), 61 (5); $[\alpha]_D$ (c = 1.385 g/100 mL, $CHCl_3$, 20 °C, ee ≥ 99%) = +75.2; CHN-Analysis calc. C: 67.88% H: 8.73% N 5.28% found C: 67.77% H: 8.70% N: 5.26%.

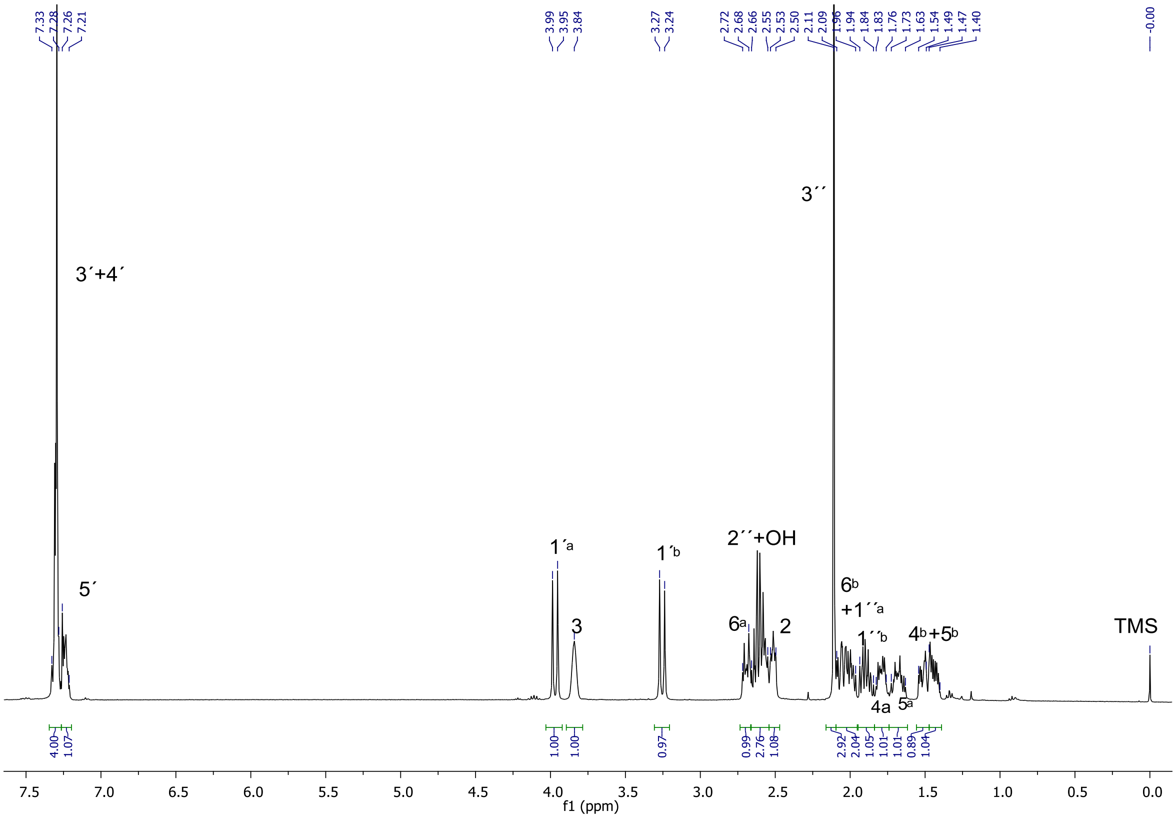

^1H NMR-spectra of piperidinol *cis*-**11d** in $CDCl_3$ (400 MHz).

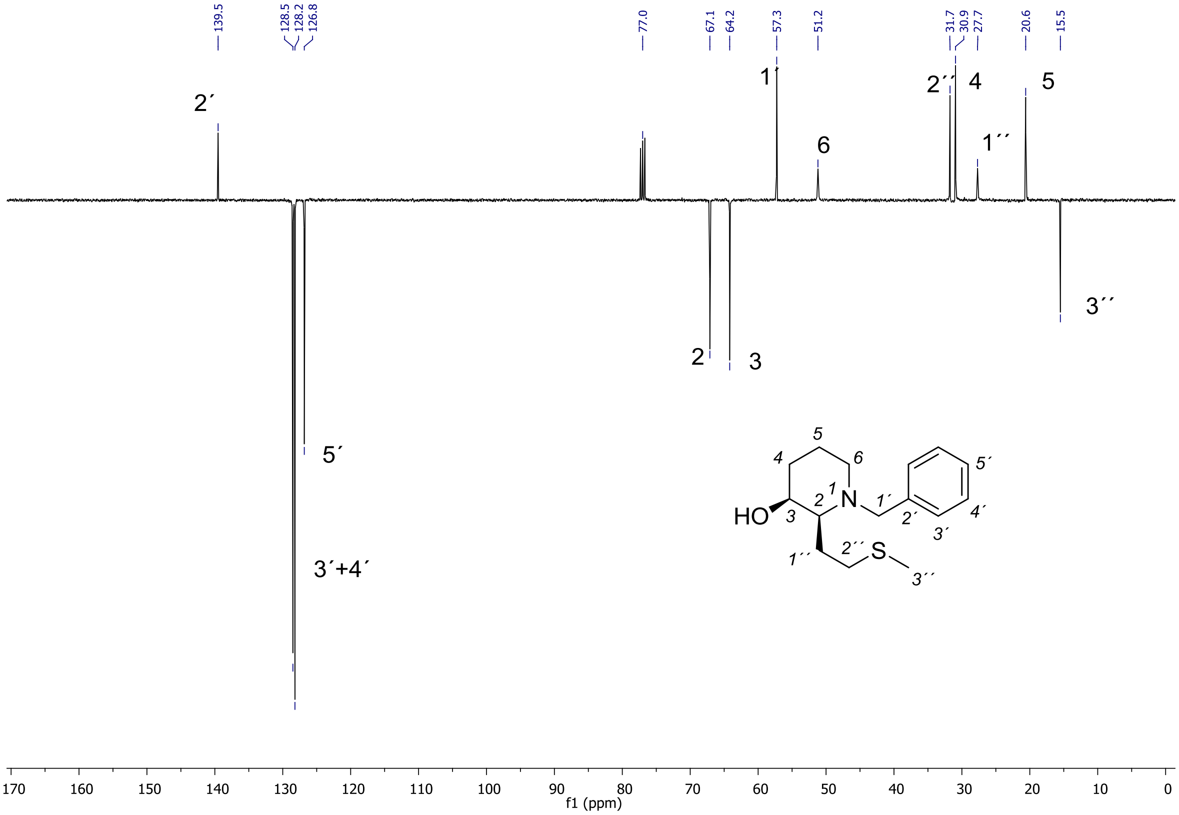

^{13}C NMR-spectra of piperidinol *cis*-**11d** in $CDCl_3$ (100 MHz).

HPLC-chromatograms of the enantioenriched piperidine *cis*-**11d** under the same separation conditions as for piperidinols *cis*-**11a–c** (separation conditions: column Chiralpak AI from Daicel Industries, flow 1 mL/min, eluent EtOH/*n*-Hex/NEt_3 1:100:0.1, detected wave length 254 nm):

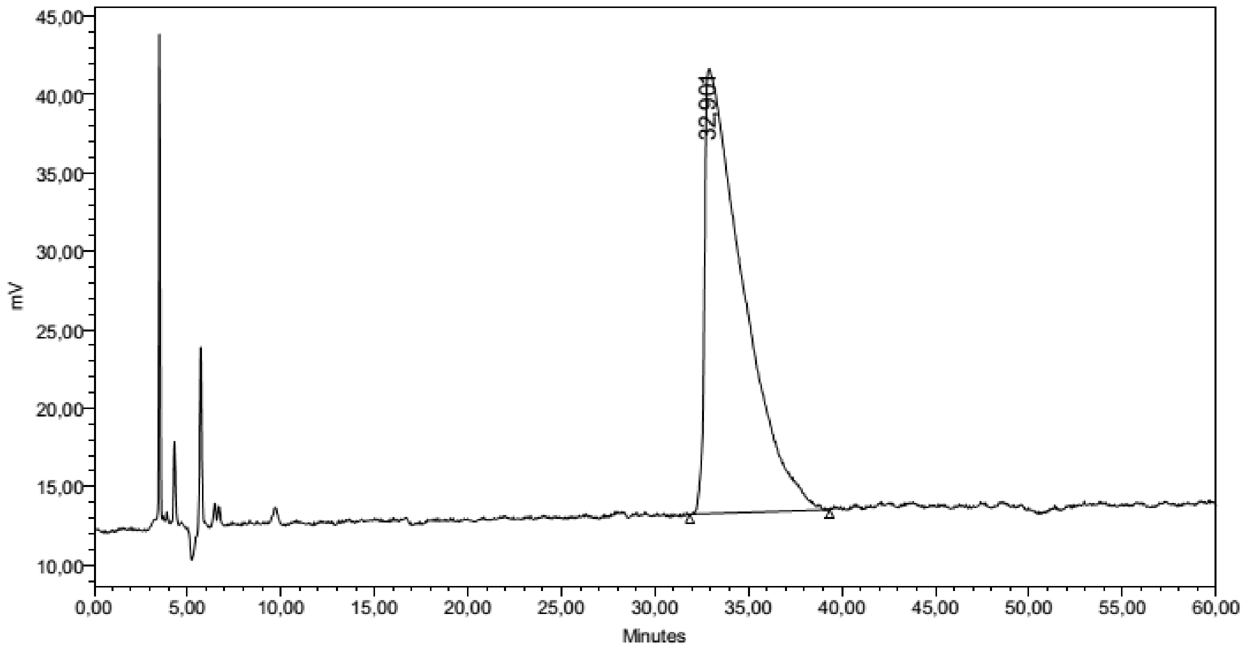

	RT	Area	% Area	Height
1	32,901	4034427	100,00	28341

3.6.2 General procedure for synthesis of piperidinols *cis*-11a–c via phosphane mediated cyclodehydration

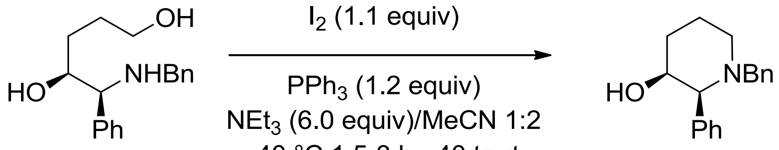

Under an atmosphere of argon the amino alcohol *syn*-**9** (1.0 equiv) and PPh_3 (1.2 equiv) were dissolved in dry MeCN and NEt_3 (6.0 equiv, reagent grade, MeCN/NEt_3 2:1) at ambient temperature. Then the reaction solution was cooled in an acetone/dry ice bath to –40 °C and iodine (1.1 equiv) was added to the clear solution in one portion. The resulting reaction suspension ($Et_3N{\cdot}HCl$ precipitated after iodine addition) was stirred at –40 °C until complete dissolution of iodine (1.5–3 h). Thereafter the reaction progress was monitored by micro work up with 1 N NaOH (aq.)/Et_2O and TLC-control or GC–MS. With amino alcohol **9a** usually showed full conversion directly after dissolution of iodine and was worked up as described below after removing of the cooling bath. The cyclodehydrations of substrates **9b–c** were allowed to warm to 0 °C remaining in the cooling bath (1–2 h) to reach complete conversion. Next the cooling bath was removed and stirring was continued for at ambient temperature until micro full conversion of diol **9** (1–2 h).

Subsequently, the mixture was diluted with MeCN (MeCN/NEt_3 2:1) silica gel (**9c**/SiO_2 1:7–1:9) was slowly added to the reaction solution under stirring and the solvent was evaporated under reduced pressure. Next, the residue was suspended in *c*-Hex, the solvent was removed again in vacuo (to eliminate traces of MeCN) and the resulting fine powder was dried in high vacuum for ca. 0.5 h [21]. After chromatographic purification on silica gel (**9**/SiO_2 1:50) with iPrOH/*c*-Hex/NEt_3 mixtures (see general procedure for the phosphite driven cyclodehydration), concentration with CH_2Cl_2 under reduced pressure (2 mL) and drying in high vacuum under stirring and heating to 40 °C for several hours delivered the desired piperidines **11**. The analytical data of the isolated products **11a–c** was in agreement with those obtained by the phosphite driven cyclodehydration.

(2*S*,3*S*)-1-Benzyl-2-methyl-3-piperidinol (*cis*-11a)

Yield: 675 mg (3.30 mmol, 77%, dr > 19:1, ee ≥ 99%, pale yellow oil).

(2*S*,3*S*)-1,2-Dibenzyl-3-piperidinol (*cis*-11b)

Yield: 298 mg (1.06 mmol, 68%, dr >19:1, ee ≥ 99%, yellow oil).

(2*S*,3*S*)-1-Benzyl-2-phenyl-3-piperidinol (*cis*-11c)

Yield: 2.257 g (→8.31 mmol, 74% considering 5 mol % of remaining CH_2Cl_2; dr = 4.0:1 *cis/trans*, *ee* = 90%, pale yellow oil) and 79 mg (0.295 mmol, 3%, dr ≥ 19:1 *cis/trans*, pale yellow oil).

3.6.3 Synthesis of (2*S*,3*S*)-1-benzyl-2-methyl-3-piperidinol (*cis*-11a) through other cyclodehydration protocols

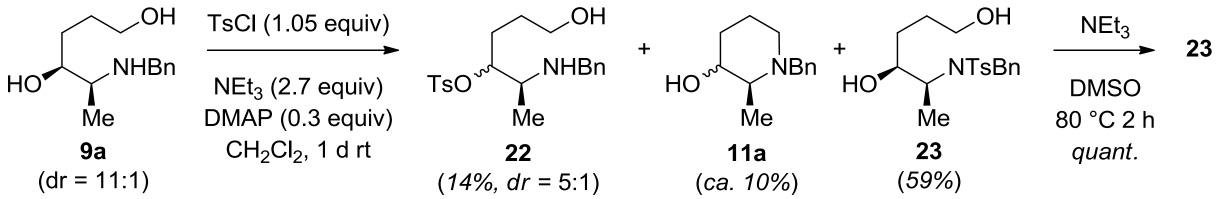

The diol **1** 104 mg, 0.464 mmol, *dr* = 11:1 *syn/anti*) was dissolved in CH_2Cl_2 (2 mL), cooled to 0 °C and treated successively with Et_3N (0.17 mL, 2.29 mmol, 2.7 equiv), TsCl (93 mg, 0.487 mmol, 1.05 equiv) and DMAP (17 mg, 0.129 mmol, 0.3 equiv). After stirring for 30 min the cooling bath was removed and the mixture allowed to stir for 1 d at room temperature.

In the following the reaction mixture was treated with 1 N NaOH solution in water (5 mL, pH ≥ 14), extracted with CH_2Cl_2 (3 x 5 mL), the combined organic phases were dried over $MgSO_4$ and concentrated under reduced pressure in order to give 202 mg of a pale yellow oil [22]. Subsequently,

[21] The mixture was concentrated with the stir bar remaining in the reaction flask, because the stir bar effected milling of the silica gel and prevented “sloshing”, and was removed before chromatographic purification. Although for instance the product **11a** is more polar (R_f = 0.24) then $OPPh_3$ (R_f = 0.49 both in (iPrOH/*n*-Hex 1:5), after column chromatography **11a** can be found in earlier fractions then $OPPh_3$. Only with residual polar solvents (e. g. CH_2Cl_2) in the crude product, triphenylphosphine oxide is eluted prior to **11a** delivering usually some mixed fractions.

22. ^{1}H NMR of the crude product showed ca. 10 mol % of the piperidine **11a**, which was not isolated after column chromatographic purification.

the crude product was purified through column chromatography on silica with EtOAc/*n*-Hex 1:2. After concentration with $CHCl_3$ (2x5 mL) and drying in high vacuum for 1 h the tosylamide **23** (103 mg, 0.273 mmol, 59%, one diastereomer according to ^1H NMR) and the tosylate **22** (23.7 mg, 0.062 mmol, 14%, *dr* = 5:1)) were isolated as colorless oils. In order to prove the constitution of amide **23**, **23** was treated with an excess of Et_3N in DMSO at 80 °C for several hours. As only starting material was reisolated the product **23** must be a tosyl amide (a tosylate with the primary or secondary OH-function should result under the applied reaction conditions either the desired piperidine **11a** or the corresponding aziridine). Additionally, the position of the tosyl moiety of **23** was validated by NOE-NMR and changes in chemical shifts compared to the starting material **9a** (see below).

***S*-5-(Benzylamino)-4-hydroxyhexan-4-methylbenzene sulfonate (22, *dr* = 5:1)**

M ($C_{13}H_{19}NO$) = 205.296 g/mol; r_f (SiO_2, EtOAc/*n*-Hex) = 0.91+0.77 (two spots, 1:1).

***N*-Benzyl-*N*-((2*S*,3*S*)-3,6-dihydroxyhexan-2-yl)-4-methylbenzene sulfonamide (23)**

M ($C_{13}H_{19}NO$) = 205.296 g/mol; r_f (SiO_2, EtOAc/*n*-Hex) = 0.26 (2:1), 0.41 (3:1); ^1H NMR (400 MHz, $CDCl_3$) δ [ppm] = 7.71 (d, 1H, H-2´´, *J* = 8.5 Hz), 7.39 (d, 2H, H-3´, *J* = 6.8 H), 7.35-7.28 (m, 5H, H-3´, H-3´´), 4.67 (d, 1H, H-1´´$_a$, *J* = 15.6 Hz), 4.10 (d, 1H, H-1´$_b$, *J* = 15.8 Hz), 3.80-3.72 (m, 1H, H-2), 3.57-3.51 (m, 2H, H-6), 3.18 (td, 1H, H-3, *J* = 8.8, 2.4 Hz), 2.51 (s, 1H, OH), 2.43 (s, 3H, H-5´´), 2.13 (s, 1H, OH), 1.61-1.50 (m, 3H, H-4$_a$, H-5), 1.33-1.21 (m, 1H, H-4$_b$), 0.91 (d, 3H, H-1, *J* = 6.8 Hz); ^{13}C NMR (100 MHz, $CDCl_3$) δ [ppm] = 143.5 (C-4´´), 137.7 (C-2´), 137.3 (C-1´´), 129.8/128.8/128.2/127.9 (C-3´, C-3´´), 127.2 (C-2´´), 72.1 (C-3), 62.6 (C-6), 59.4 (C-2), 47.9 (C-1´), 30.3 (C-4), 28.5 (C-5), 21.5 (C-5´´), 14.5 (C-1); IR (film) ν [cm^{-1}] = 3515 (br), 3391 (br), 3108 (w), 3087 (w), 3063 (w), 3031 (w), 2927 (s), 2872 (m), 1814 (w), 1724 (w), 1652 (w), 1598 (w), 1495 (m), 1455 (m), 1391 (w), 1333 (vs.), 1306 (m), 1290 (m), 1204 (w), 1152 (vs.), 1092 (m), 1055 (w), 1026 (w), 1010 (w), 920 (w), 865 (w), 815 (w), 765 (w), 730 (s), 698 (w), 658 (s), 600 (w), 550 (s); HR-MS (ESI, $[C_{20}H_{28}NO_4S]^+$) calc. 378.1739 u, found 378.1736 u; $[\alpha]_D$ (c = 0.766 g/100 mL, $CHCl_3$, T = 21.5 °C) = +8.7.

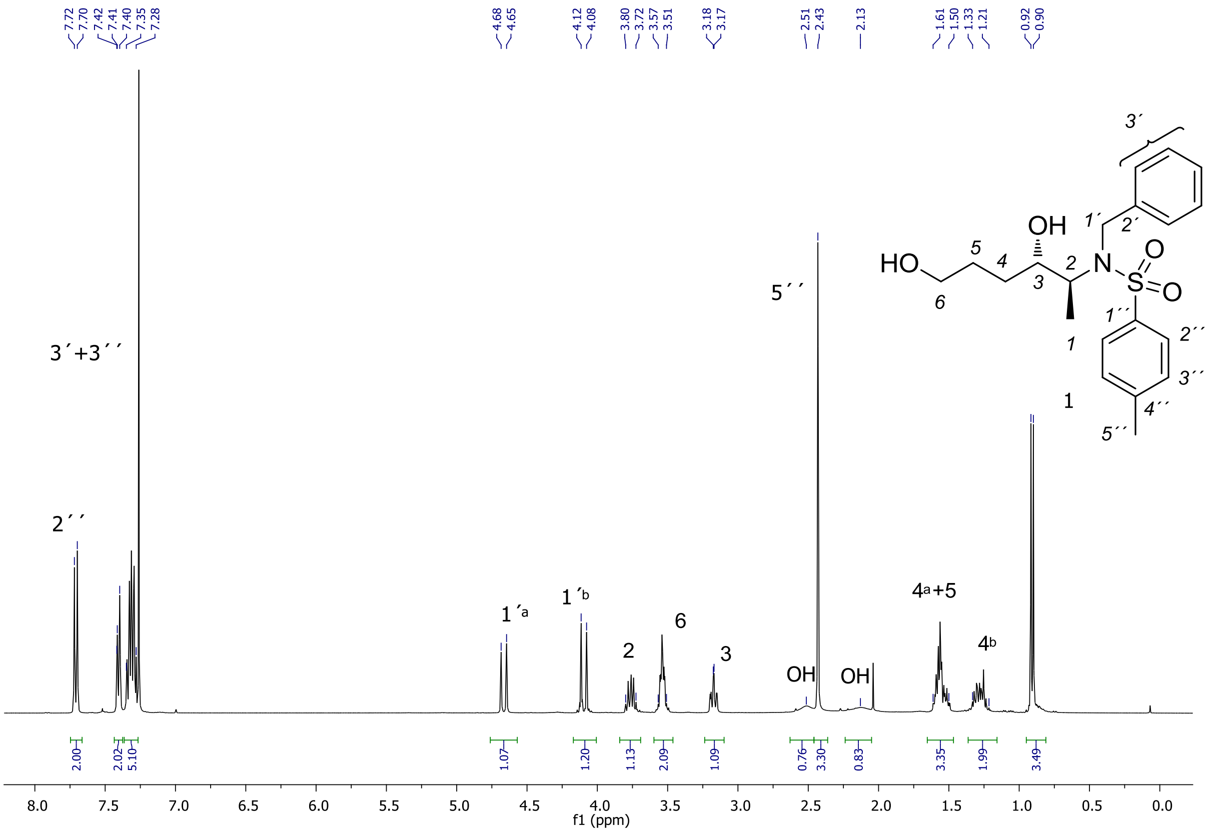

^{1}H NMR-spectra of the tosyl amide **23** in $CDCl_3$ (400 MHz).

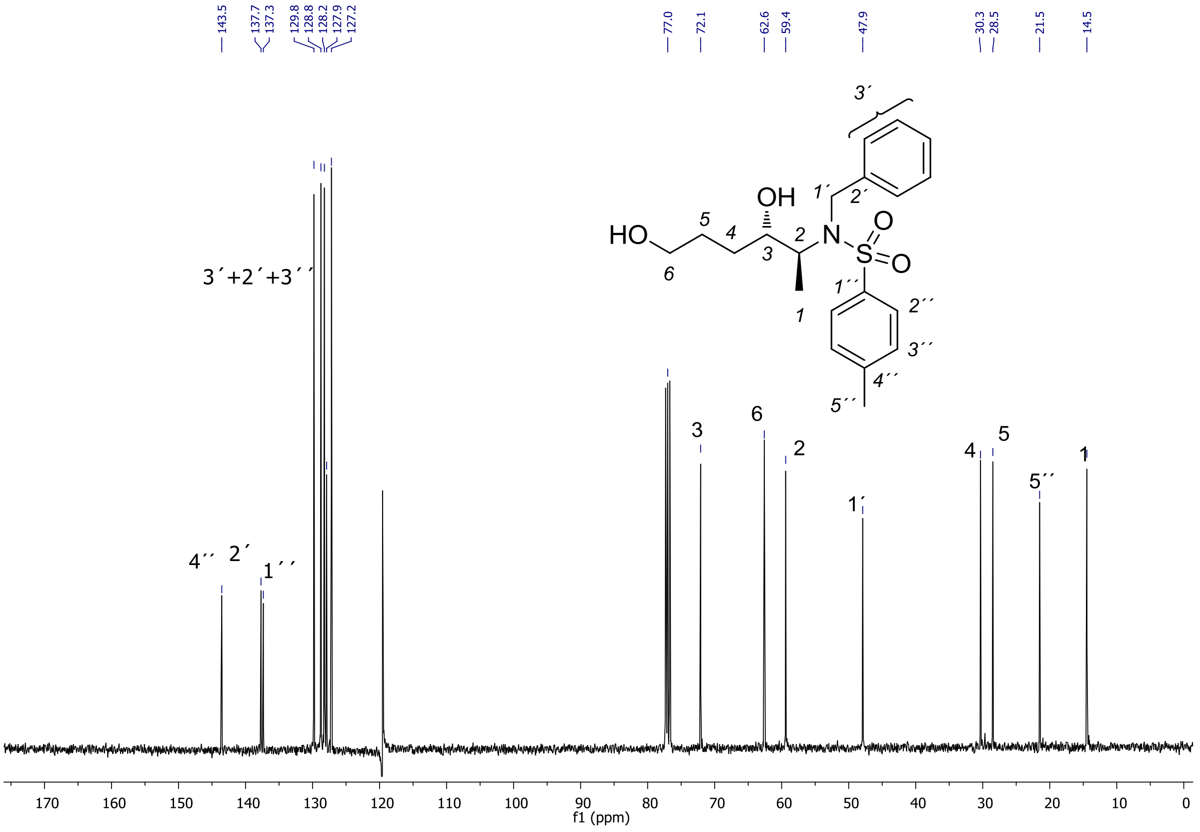

^{13}C NMR-spectra of the tosyl amide **23** in $CDCl_3$ (100 MHz).

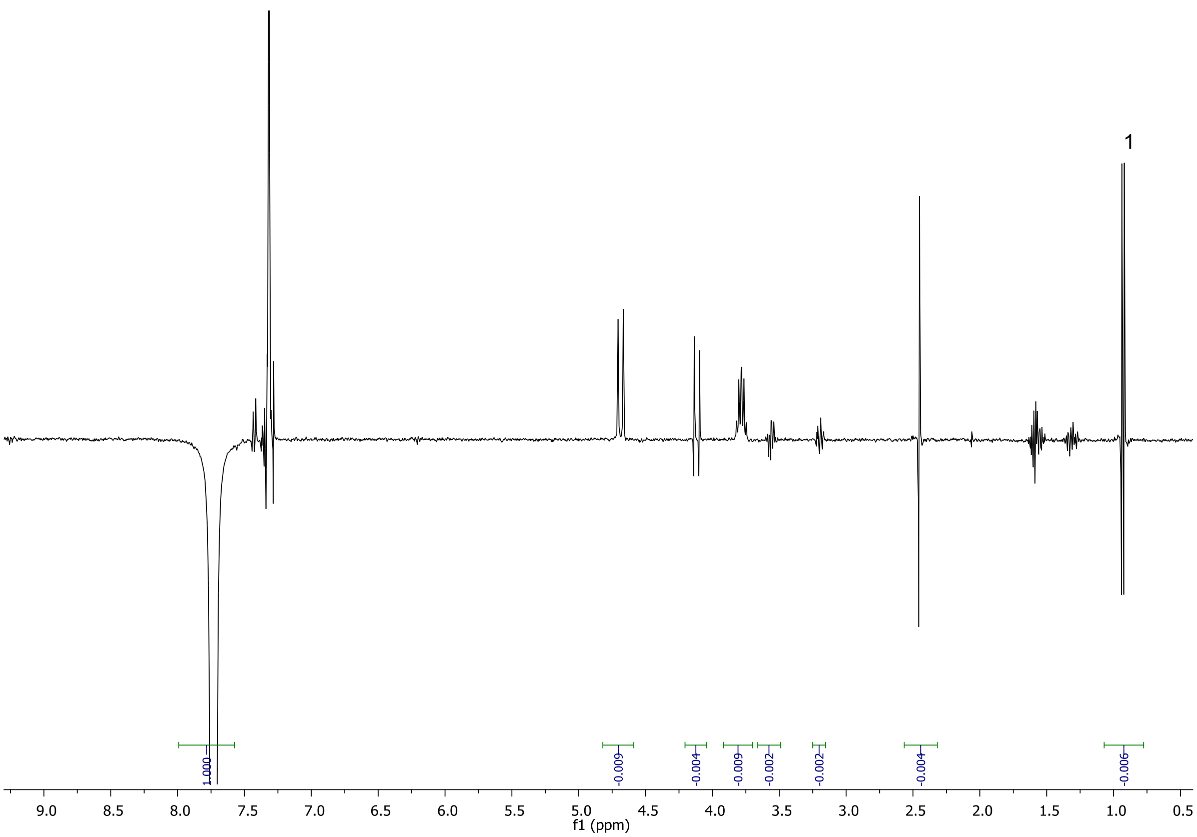

NOE-NMR-spectra (irradiation on H-2´) of the tosyl amide **23** in $CDCl_3$ (400 MHz).

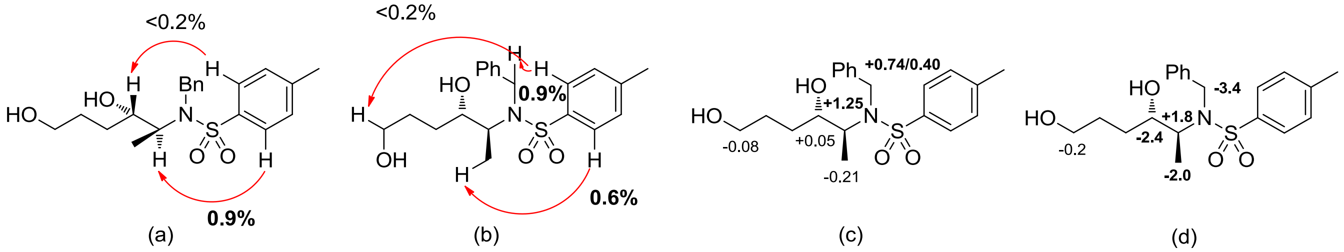

(a) and (b) observed NOE effects (irradiation on H-2´); changes of chemical shift in ^{1}H (c) and ^{13}C NMR (d) spectra compared to the starting material **9a** (“+” = shift to lower field, “-“ = shift to higher field).

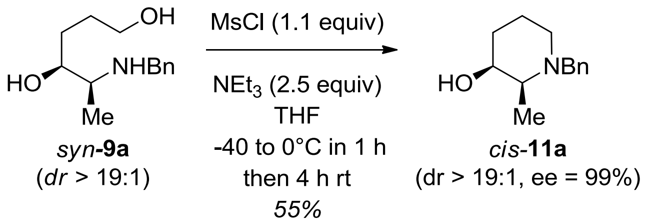

Under an atmosphere of argon the diol **9a** (205 mg, considering 13 mol residual CH_2Cl_2 → 0.88 mmol, dr > 19:1 *syn*/*anti*) was dissolved in THF (2 mL), Et_3N (0.30 mL, 2.20 mmol, 2.5 equiv) was added and the reaction solution was cooled to –40 °C. Then (75 μL, 0.97 mmol, 1.1 equiv) of MsCl were added as a solution in dry THF (1 mL) within 5 min, whereby NEt_3·HCl precipitated. The reaction suspension was allowed to warm to 0 °C within 1 h and the cooling bath was removed.

After 4 h of stirring at room temperature, the mixture was cooled in an ice bath and saturated $NaHCO_3$ solution (aq., 4 mL) was added. Subsequently, the mixture was extracted with EtOAc (3 x 4 mL), the combined organic phases were dried over $MgSO_4$ and concentrated under reduced pressure. The crude product was dissolved CH_2Cl_2 (5 mL), SiO_2 (0.7 g) was added and the solvent was evaporated under reduced pressure. After column chromatic purification on silica gel with iPrOH/*n*-Hex/Et_3N 2.5:100:0.8, concentration with $CHCl_3$ (2 x 5 mL) and drying in high vacuum for 3 h the piperidine **11a** (100 mg, 0.49 mmol, 55%) was obtained as a yellow oil (ee ≥ 99%, dr > 19:1). ^{1}H NMR indicated approximately 5 mol % of an unidentified side product.

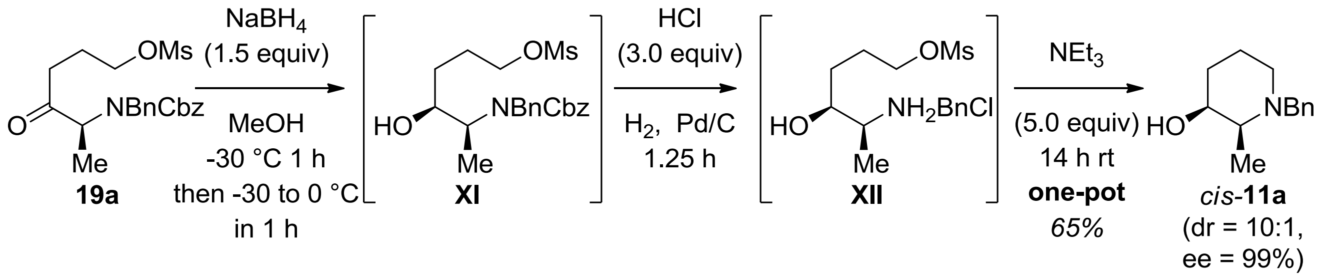

The ketone **19a** (397 mg, 0.92 mmol, for the synthesis see Supporting Informations of [2].) was dissolved in MeOH (3 mL, [**19a**] = 0.3 mol/L) and cooled to –30 °C. Thereby, approximately at –20 °C the starting material precipitated partly as a colorless solid. Next, $NaBH_4$ (52 mg, 1.38 mmol, 1.5 equiv) was added in one portion, the reaction mixture was stirred for 1 h at –30 °C and was then slowly brought to 0 °C remaining in the cooling bath over a time period of 1 h. Micro work up with 1 N HCl solution and EtOAc and TLC control revealed full conversion from **19a** (R_f (EtOAc/*n*-Hex 1:1) = 0.78) to the diol **XI** (R_f (EtOAc/*n*-Hex 1:1) = 0.38). After the addition of 32% HCl solution in water (0.27 mL, 2.76 mmol, 3.0 equiv), the cooling bath was removed, the reaction solution was degassed in high vacuum, commercial 10 wt % Pd/C (39 mg 0.037 mmol, 0.04 equiv) was added and the reaction suspension was stirred for 1.25 h under one atmosphere of H_2 at ambient temperature, whereon TLC control indicated full conversion from the carbamate **XI** to the ammonium salt **XII**.

To induce the cyclisation, Et_3N (0.64 mL, 4.60 mmol, 5.0 equiv) was added. After stirring for 14 h at ambient temperature, the reaction mixture was diluted with MeOH (5 mL), filtered through a thin layer of celite, the celite cake was washed with MeOH (2x5 mL) and the combined red colored filtrates were concentrated under reduced pressure. The residue was treated with 2 N NaOH (4 mL) solution in water, extracted with Et_2O (3 x 4 mL), the collected Et_2O phases were dried over $MgSO_4$ and concentrated under reduced pressure. In the following the crude product (238 mg of a red oil) was subjected to column chromatographic purification on silica gel with iPrOH/*n*-Hex/Et_3N 2.5:100:0.8 (thereby the crude product was dissolved in a minimum amount of the eluent). After concentration with CH_2Cl_2 (2 x 2mL) at the rotatory evaporator and drying in high vacuum for 2 h the piperidine **11a** (124 mg, 0.602 mmol, 65%) was obtained as a pale yellow oil with a dr of 10:1 *cis/trans* (according to ^{1}H NMR) and an ee ≥ 99%.

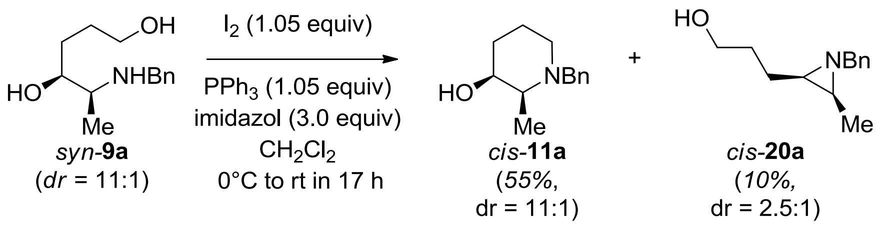

The diol **9a** (99 mg, 0.442 mmol, dr = 11:1 *syn/anti*) was dissolved in CH_2Cl_2 (2 mL), treated with imidazol (90 mg, 1.33 mmol, 3.0 equiv) and PPh_3 (120 mg, 0.404 mmol, 1.05 equiv) and cooled to 0 °C in an ice bath. Subsequent, iodine (115 mg, 0.464 mmol, 1.05 equiv) was added in two portions. The mixture was stirred at 0 °C until iodine had dissolved completely (15 min), the cooling bath was removed and the reaction solution was allowed to stir for 17 h at ambient temperature.

Then 1 N HCl solution in water (4 mL) was added, the aqueous acidic phase was washed with MTBE (3 x 4 mL), treated with saturated, aqueous $NaHCO_3$ solution (6 mL, pH = 7) and 3 N NaOH (1 mL) solution in water (pH ≥ 10) and extracted with EtOAc (3 x 5 mL) [23]. The combined EtOAc-extraction phases were dried over $MgSO_4$ and concentrated under reduced pressure to give 105 mg crude product as pale yellow oil. After column chromatographic purification on silica gel with MeOH/EtOAc 1:20 as eluent, concentration with $CHCl_3$ (2 x 5 mL to remove NEt_3) and drying in high vacuum for 1 h the piperidinol *cis*-**11a** (50 mg, 0.244 mmol, 55%, dr = 11:1 *cis/trans*) and the aziridine **20a** (9 mg 0.044 mmol, 10%, dr = 2.5:1 *cis/trans*) were isolated, both as pale yellow oils. The relative configuration of the minor and major diastereomer of **20a** was proven by NOE-NMR-spectroscopy. The important NOEs crucial for the assignment of the relative stereochemistry are indicated below

(2*S*,3*S*) and (2*R*,3*S*)-1-Benzyl-2-(3-hydroxypropyl)-3-methylaziridine (20a)

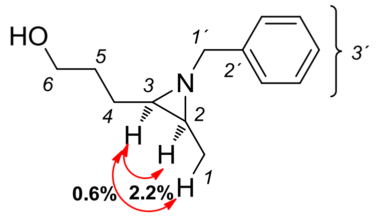

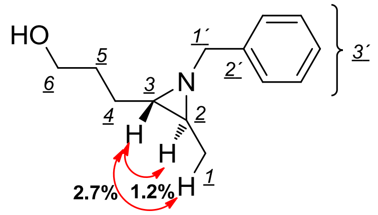

M ($C_{13}H_{19}NO$) = 205.296 g/mol; **r$_f$** (SiO_2, MeOH/CH_2Cl_2) = 0.35 (1:15); **^{1}H NMR** (400 MHz, $CDCl_3$, dr = 2.6:1 *cis/trans*) δ [ppm] = 7.36-7.21 (m, 10H, H-3´, H-3´), 4.32 (p, 1H, H-3, *J* = 4.4 Hz), 4.11-4.04 (m, 1H, H-2), 3.78 (d, 1Hm H-1´$_a$, *J* = 13.4 Hz, *dr* = 2.9:1 *cis/trans*), 3.55 (d, 1H, H-1´$_a$, *J* = 12.9 Hz), 3.43 (d, 1H, H-1´$_b$, *J* = 13.2 Hz, *dr* = 2.3:1 *cis/trans*), 3.36 (d, 1H, H-1´$_b$, *J* = 12.9 Hz), 3.14-3.08 (m, 1H, H-2), 2.93-2.87 (m, 2H, H-3, H-6$_a$), 2.66-2.60 (m, 1H, H-6$_a$), 2.36-2.31 (m, 1H, H-6$_b$, *dr* = 2.7:1 *cis/trans*), 2.29-2.24 (m, 1H, H-6$_b$), 2.02-1.58 (m, 8H, H-4, H-5, H-4, H-5), 1.52 (d, 3H, H-1, *J* = 7.0 Hz, *dr* = 2.5:1 *cis/trans*), 0.85 (d, 3H, H-1, *J* = 6.8 Hz); **^{13}C NMR** (100 MHz, $CDCl_3$) δ [ppm] = 139.4 (C-2´), 139.0 (C-2´), 128.8/128.7/128.6/ 128.5/2x128.3 (C-3´, C-3´), 68.4 (C-3), 60.8 (C-2), 58.8 (C-1´), 58.5 (C-2), 58.3 (C-3), 57.1 (C-1´), 54.9 (C-6), 46.9 (C-6), 27.9 (C-4), 26.4 (C-5), 23.8 (C-5), 23.5 (C-4), 16.8 (C-1´), 7.9 (C-1); **IR** (film) ν [cm^{-1}] = 3105 (w), 3084 (w), 3060 (w), 3026 (w), 2940 (m), 2871 (w), 2798

23. Instead of an extraction funnel a 20 mL syringe was used for the work up.

(w), 1722 (w), 1602 (w), 1494 (m), 1451 (w), 1376 (w), 1275 (w), 1220 (m), 1142 (w), 1109 (w), 1076 (w), 1038 (w), 1027 (w), 905 (w), 812 (w), 772 (vs), 733 (m), 698 (m), 664 (w); **HR-MS** (ESI, $[C_{13}H_{20}NO]^+$) calc. 206.1545 u, found 206.1550 u.

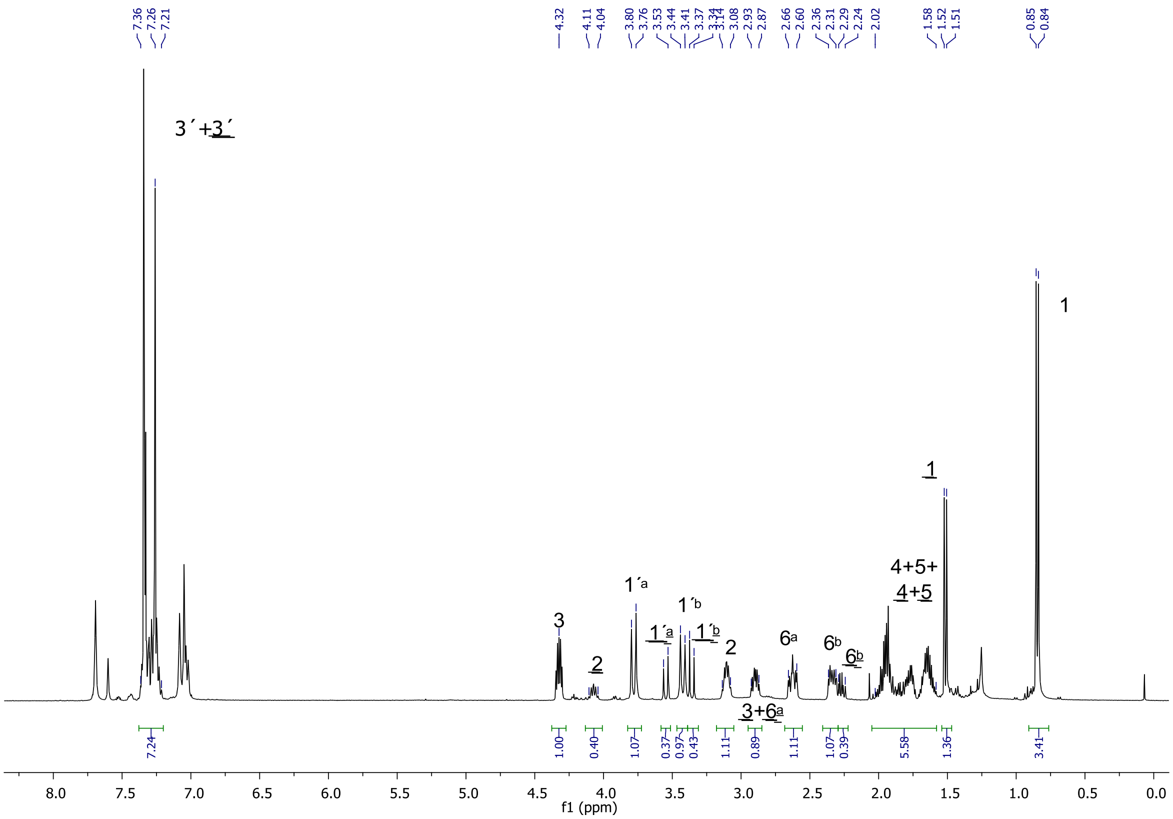

^{1}H NMR-spectra of a 2.5:1 mixture of the *cis*- and *trans*-epimers of aziridine **20a** in $CDCl_3$ (400 MHz).

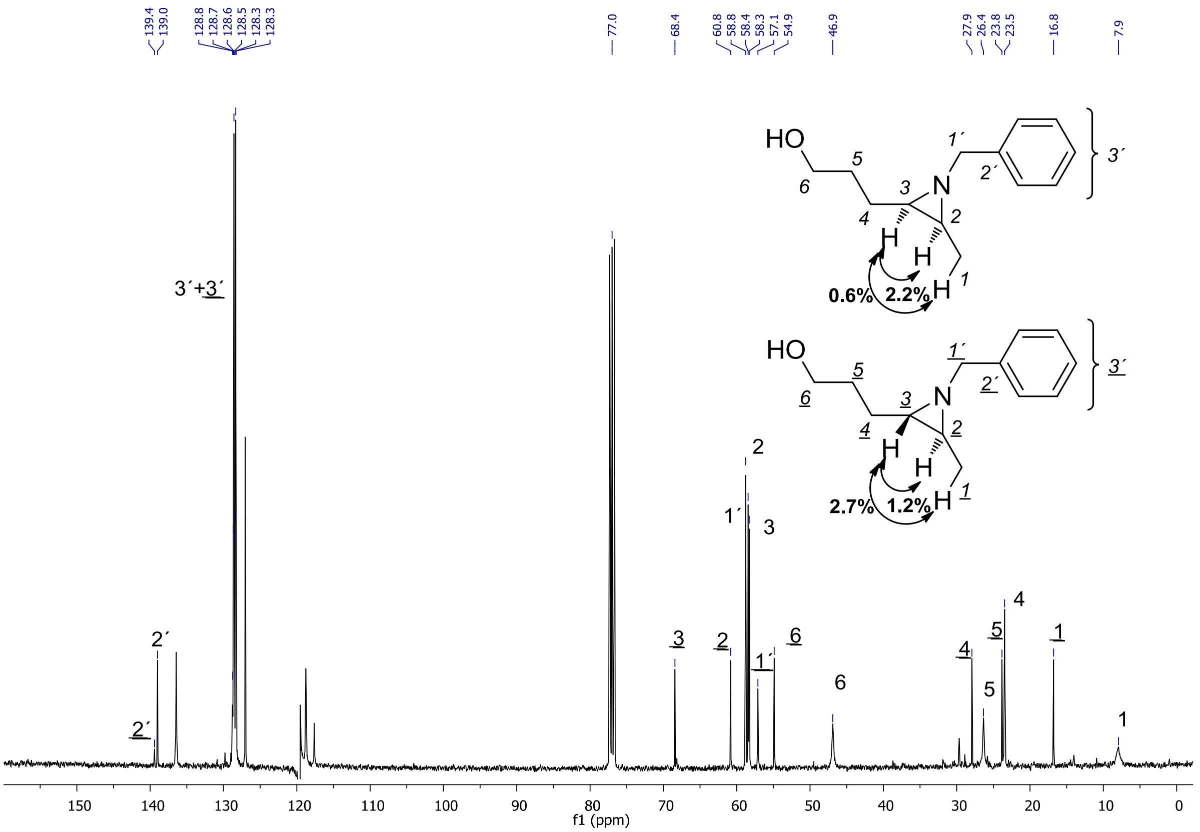

^{13}C NMR-spectra of a 2.5:1 mixture of the *cis*- and *trans*-epimers of aziridine **20a** in $CDCl_3$ (100 MHz).

3.7 Synthesis of heterocycles 13a–h and cyclisation precursors 12a–h (Table 2)

3.7.1 General procedure for the synthesis of Bn-protected amino alcohols 12a,b

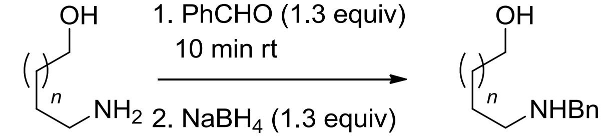

The amino alcohol **21** (1.0 equiv) was dissolved in MeOH ([**21**] = 0.5 mol/L), then benzaldehyde [24] (1.3 equiv) was added and the mixture was allowed to stir for 10 min at room temperature. Subsequently, the reaction was cooled in an ice bath, $NaBH_4$ (1.3 equiv) was added portionwise, the mixture was stirred for 0.25 h at 0 °C and 2 h at ambient temperature.

24. Benzaldehyde (10 mL) was diluted with 1 mL of Et_2O (so improve phase separation in the following washing), washed with three 5 mL portions of saturated $NaHCO_3$-solution in water and brine (5 mL) and dried over $MgSO_4$. Finally, Et_2O was removed under reduced pressure.

Next, the reaction suspension was cooled again to 0 °C, conc. HCl-solution in water (3.0 equiv) was added dropwise and MeOH was removed under reduced pressure (→ 100 mbar). The residue was diluted with water (1 mL/1 mmol **21**) and Et_2O (H_2O/Et_2O 2:1; pH of the mixture ≤ 0), the phases were separated and the aqueous phase was washed with further Et_2O (2x, H_2O/Et_2O 2:1). In the following, the aqueous phase was cooled in an ice bath, KOH (2.5 equiv) was added and dissolved under stirring, whereby an oil separated. The basic mixture was extracted with Et_2O (3x, H_2O/Et_2O 2:1), the combined extraction phases were dried over $MgSO_4$ and concentrated under reduced pressure. The crude benzylamine **12** was dissolved in 1 mL of chloroform and concentrated under reduced pressure (2x) to remove residual Et_2O and dried in high vacuum for 0.5 h under stirring (until the gas evolution ceased).

4-(Benzylamino)butanol (12a)

Yield: 541 mg (3.01 mmol, *quant*., colorless oil). Analytical data are given in the Supporting Information of [2].

5-(Benzylamino)pentanol (12b)

Yield: 896 mg (4.64 mmol, 93% colorless oil).

M ($C_{12}H_{19}NO$) = 193.285 g/mol; **^{1}H NMR** (400 MHz, $CDCl_3$) δ [ppm] =7.37-7.22 (m, 5H, H-3´), 3.78 (s, 2H, H-1´), 3.62 (t, 2H, H-5, *J* = 6.5 Hz), 2.65 (t, 2H, H-1, *J* = 7.0 Hz), 1.65-1.61 (m, 6H, H-2, H-4, NH, OH), 1.51-1.45 (m, 2H, H-3); **^{13}C NMR** (100 MHz, $CDCl_{3,}$) δ [ppm] = 140.3 (C-2´), 128.4/128.1/126.9 (C-3´), 62.7 (C-5), 54.1 (C-1´), 49.2 (C-1), 32.5 (C-4), 29.6 (C-2), 23.4 (C-3); **IR** (ATR) ν [cm^{-1}] = 3280 (br), 3082 (w), 3059 (w), 3025 (w), 2925 (s), 2854 (s), 1946 (w), 1873 (w), 1808 (w), 1602 (w), 1493 (w), 1452 (s), 1359 (w), 1308 (w), 1260 (w), 1200 (w), 1101 (m), 1075 (m), 1053 (s), 1028 (m), 730 (vs), 695 (vs); **GC-MS** (EI, 70 eV) m/z (%) = 193 (1, $[M]^+$) 120 (35), 106 (5), 91 (100, $[Bn]^+$), 77 (5, $[Ph]^+$), 65 (10, $[Cp]^+$), 51 (5), 39 (5).

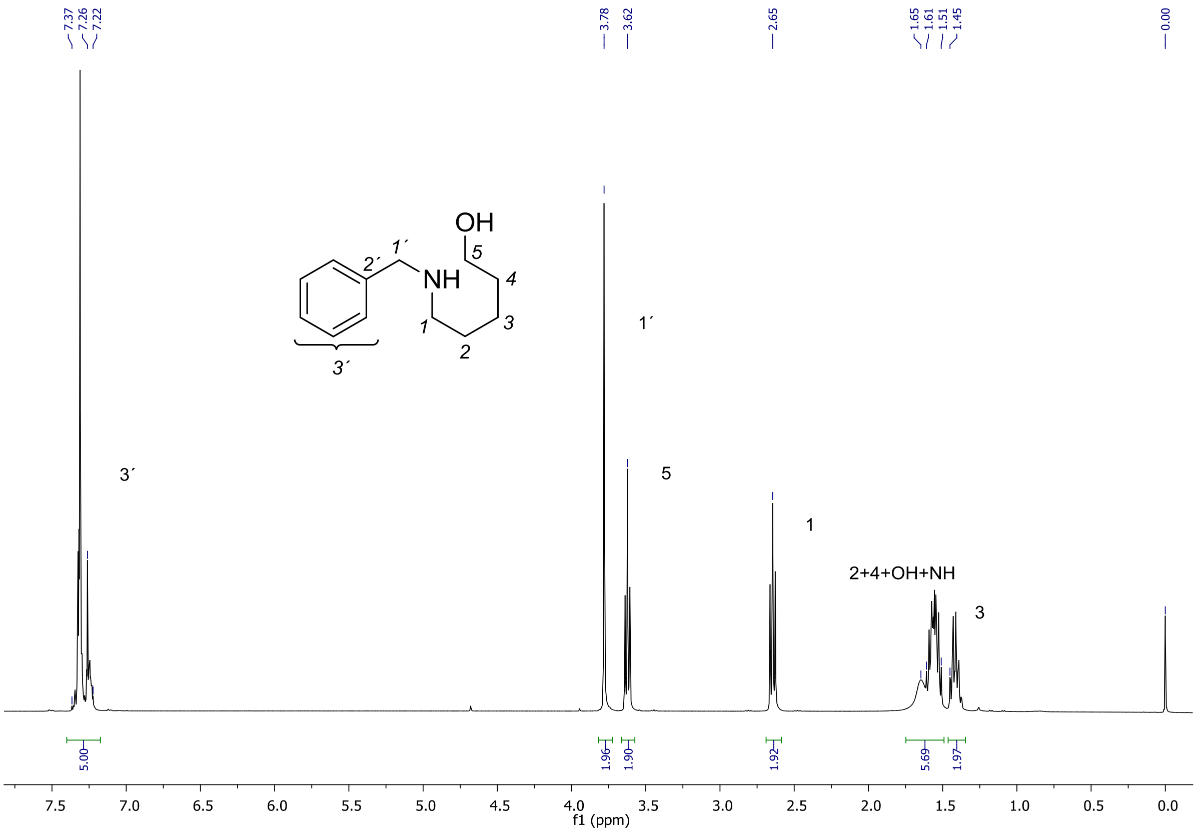

^{1}H NMR-spectra of the amino alcohol **12b** in $CDCl_3$ (400 MHz).

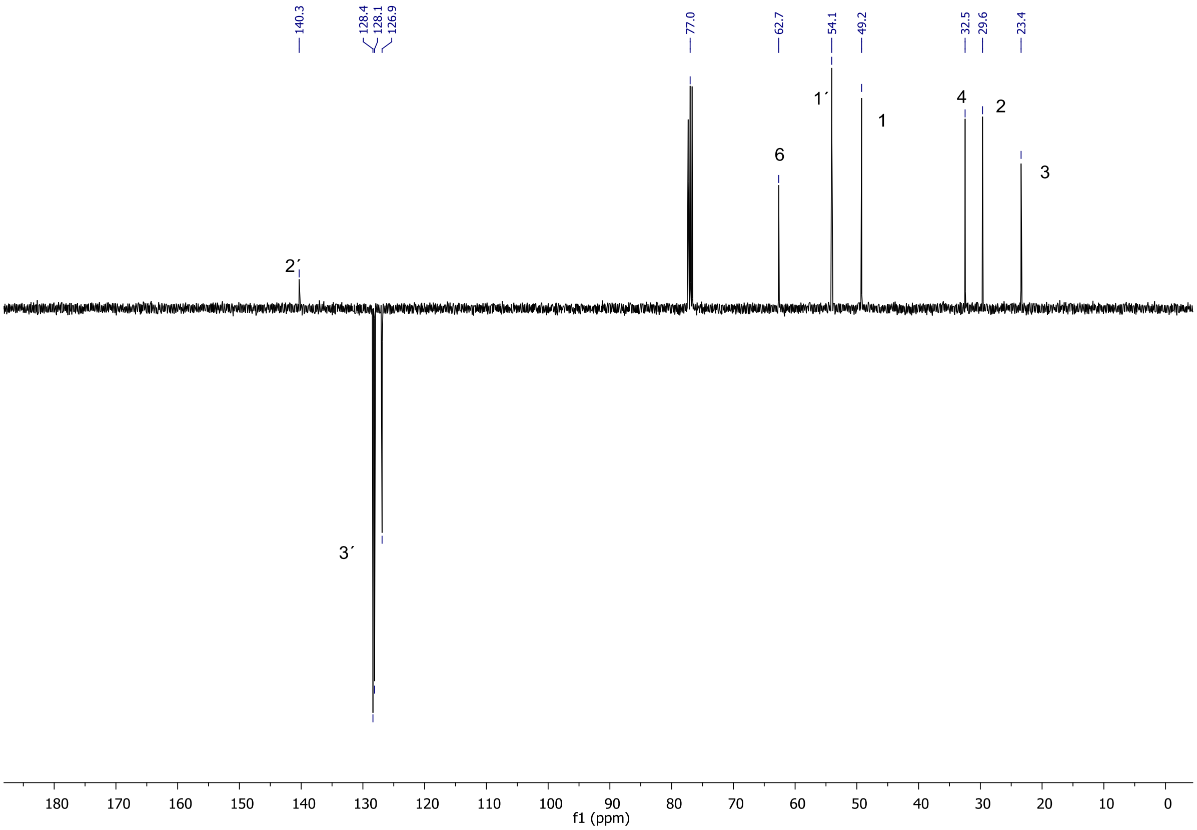

^{13}C NMR-spectra of the amino alcohol **12b** in $CDCl_3$ (100 MHz).

3.7.2 Synthesis of 4-(*tert*-butyloxycarbonylamino)-1-butanol (12c)

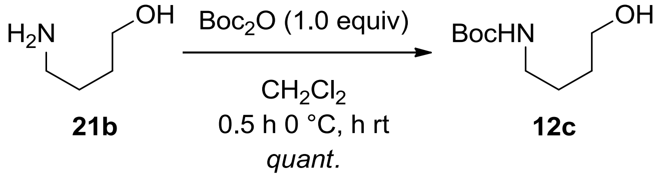

The amino alcohol **21b** (1.0 equiv, 2.87 mmol, 0.30 mL) was dissolved in 3 mL of CH_2Cl_2 ([**21b**] = 0.5 mL/L), cooled in an ice bath and Boc_2O (1.0 equiv, 2.87 mmol, 0.63 mL) was added. After stirring for 0.5 h at 0 °C, the cooling bath was removed and the mixture was allowed to stir for 2 h at ambient temperature. Subsequently, 3 mL of 1 N HCl solution (aq.) were added, the phases were separated, the organic layer was washed successively with 1 N HCl-, saturated $NaHCO_3$ solution in water and brine/H_2O 1:1 (each 3 mL) and dried over $MgSO_4$. After concentration with 2 mL of chloroform under reduced pressure for two times and drying in high vacuum under stirring for 0.5 h delivered the carbamate **12c** (2.99 mmol, quant., 567 mg) as a colorless oil.

M ($C_9H_{19}NO_3$) = 189.252 g/mol; **^{1}H NMR** (300 MHz, $CDCl_3$) δ [ppm] = 4.77 (s, 1H, NH), 3.66 (s, 2H, H-4), 3.15-3.14 (m, 2H, H-1), 2.58-2.35 (m, 1H, OH), 1.63-1.58 (m, 4H, H-2. H-3), 1.44 (s, 9H, H-3´´), **^{13}C NMR** (75 MHz, $CDCl_3$) δ [ppm] = 156.1 (C-1´), 79.1 (C-2´), 62.2 (C-4), 40.2 (C-1), 29.6 (C-3), 28.4 (C-3´), 26.5 (C-2); **IR** (ATR) ν [cm^{-1}] = 3342 (br), 3001 (w), 2974 (m), 2932 (m), 2868 (w), 1697 (vs), 1681 (vs), 1536 (s), 1503 (s), 1454 (m), 1391 (m), 1364 (s), 1275 (s), 1249 (s), 1165 (vs), 1038 (m), 912 (m), 865 (w), 729 (s); **GC-MS** (EI, 70 eV) m/z [u] = 133 (10, $[M-H_2C=C(CH)_3]^+$), 116 (5, $[M-OtBu]^+$), 98 (5), 88 (3, $[M-CO_2tBu]^+$) 71 (5), 57 (100, $[tBu]^+$), 41 (55, $[allyl]^+$).

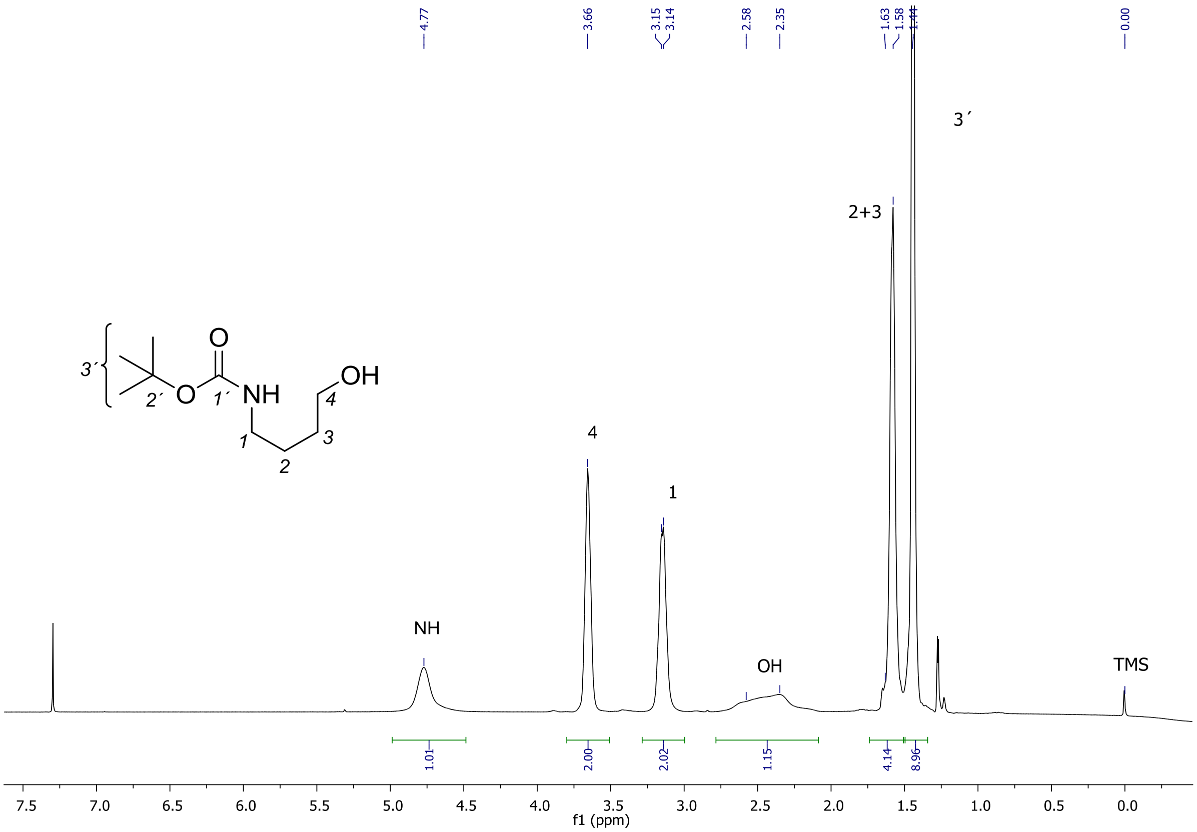

^{1}H NMR-spectra of the carbamate **12c** in $CDCl_3$ (400 MHz).

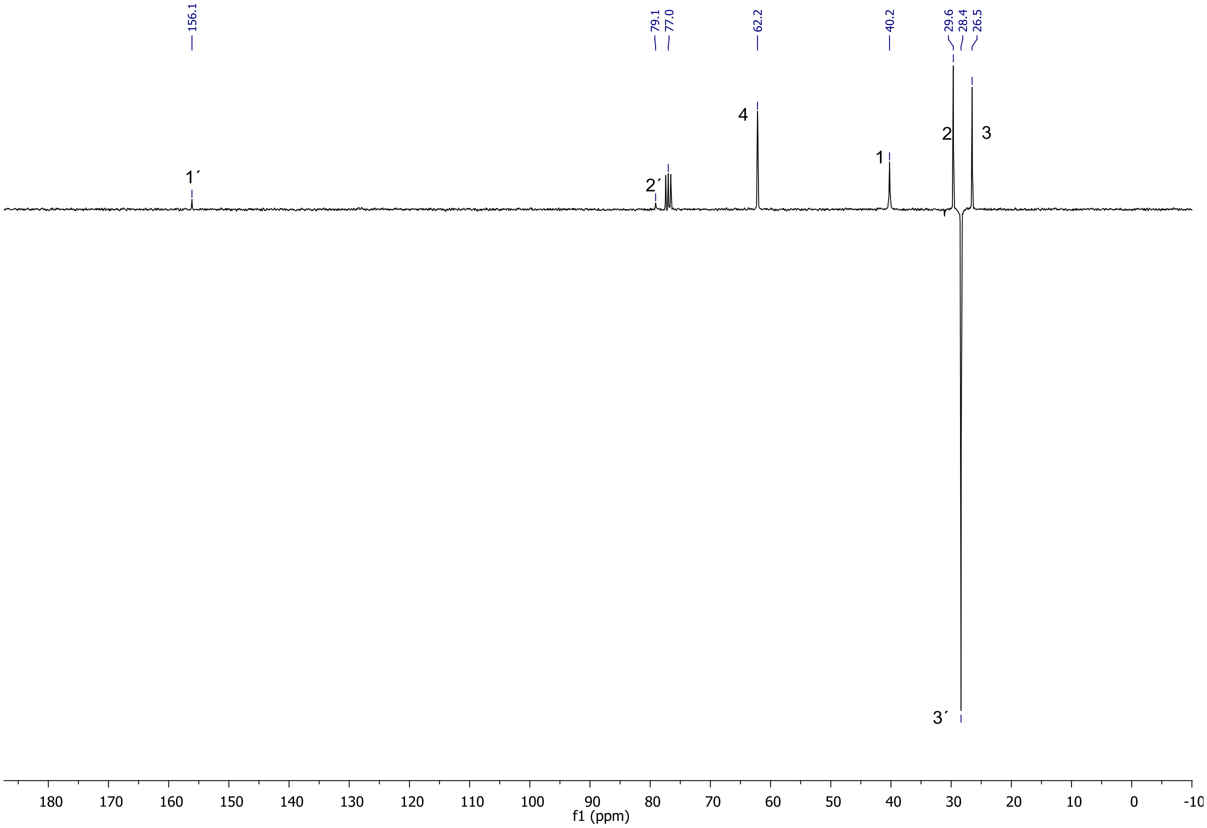

^{13}C NMR-spectra of the carbamate **12c** in $CDCl_3$ (100 MHz).

3.7.3 General procedure for the addition of Grignard-reagent 6 with carbonyl compounds 21d–h

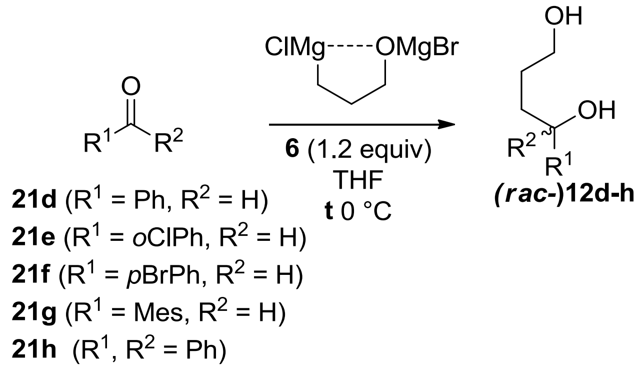

Under an atmosphere of argon the carbonyl compound **21** (1.0 equiv, 3–5 mmol) was dissolved in dry THF ([**21**] = 1 mol/L) and cooled to 0 °C. Then a 0.30 N solution of ClMg*n*PrOMgBr in THF/MeTHF (1.2 equiv) was added dropwise within 10–20 min. After stirring for a time period *t* at 0 °C, the reaction was quenched through the addition of saturated NH_4Cl-solution in water (2 mL/1 mmol **6**).

The THF/MeTHF was evaporated under reduced pressure (→200 mbar), the remaining mixture was diluted with water to dissolve precipitated NH_4Cl (usually 1/3 to 1/4 of the volume of the NH_4Cl solution) and CH_2Cl_2 (H_2O/CH_2Cl_2 2:1). If a precipitate still remained after adding water (up to 1/3 of the original volume of the aqueous phase), the mixture was neutralized to pH = 6–8 with conc. HOAc. Then the aqueous phase was separated and extracted with two further portions of CH_2Cl_2. The combined CH_2Cl_2-phases were dried over $MgSO_4$ and concentrated under reduced pressure. Finally the crude diol **12** was purified through column chromatography on silica (SiO_2/**12** 30–20:1) with EtOAc/*c*-Hex as eluent mixtures, concentrated in vacuo with chloroform (2x) and dried in high vacuum with stirring for a couple of hours. Analytical data of compounds **12d–h** are given in the Supporting Information of [2].

1-phenyl-1,4-butandiol (R^1 = Ph, R^2 = H; *rac*-12d)

t: 5 min; eluent: EtOAc/*c*-Hex 3:1 (crude **12d** dissolved in eluent); yield: 79% (655 mg, 3.94 mmol, colorless oil).

***rac*-1-(2-Chlorophenyl)-1,4-butandiol (R^1 = *o*ClPh, R^2 = H; *rac*-12e)**

t: 30 min; eluent: EtOAc/*c*-Hex 1:1→3:2 (crude **12e** was dissolved in the eluent); yield: 94% (668 mg, 3.35 mmol, colorless oil).

***rac*-1-(4-Bromophenyl)-1,4-butandiol (R^1 = *p*BrPh, R^2 = H; *rac*-12f)**

t: 20 min; eluent: EtOAc/*c*-Hex 2:1→5:2 (crude **12f** was dissolved in CH_2Cl_2 under heating) yield: 84% (982 mg, 4.01 mmol, colorless solid).

***rac*-1-(2,4,6-Trimethylphenyl)-1,4-butandiol (R^1 = 2,4,6-Me_3Ph, R^2 = H; *rac*-12g)**

t: 1 h; eluent: EtOAc/*c*-Hex 3:2 (crude **12g** was dissolved in the eluent); yield: 90% (631 mg, 3.03 mmol, colorless solid).

1,1-Diphenyl-1,4-butandiol (R^1, R^2 = Ph 12h)

t: 1.5 h; eluent: no chromatographic purification necessary; yield: quant. (1.276 g, 5.27 mmol, colorless solid).

3.7.4 General procedure for the synthesis of heterocycles 13a,b and *rac*-13d–h

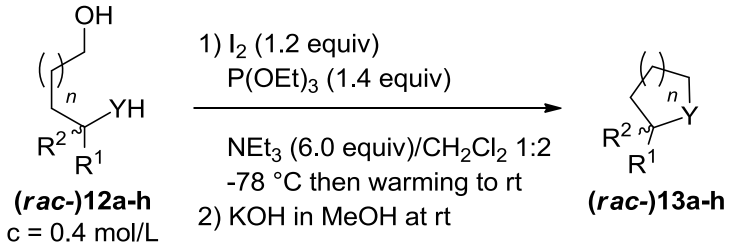

Under an atmosphere of argon the amino alcohol/diol (***rac***-)**12** (1.0 equiv, 1–2 mmol) was dissolved in NEt_3 (6.0 equiv) and dry CH_2Cl_2 (NEt_3/CH_2Cl_2 1:2), the mixture was cooled to –78 °C in an acetone/dry ice bath and treated with $P(OEt)_3$ (1.4 equiv). Subsequently, iodine (1.2 equiv) was added in one portion and the mixture was stirred at –78 °C until complete dissolution of iodine (2.5–5 h). Then the cooling bath was removed and the clear reaction solution was allowed to stir at ambient temperature for 0.5 h [25].

Work up with saponification of $OP(OEt)_3$ (all substrates)

In order to quench the reaction 4 N KOH-solution in MeOH (5.0 equiv) was added to the reaction suspension, the solvent was removed in vacuo (→ 50 mbar) and the residue was dried under reduced pressure (50 mbar) in a rotatory evaporator for 5–10 min. The residue was dissolved in 3 mL of H_2O and 2 mL of *n*-Pen, the organic phase was separated, the aqueous phase was extracted with two further 2 mL portions of *n*-Pen. To remove remaining traces of $O=P(OEt)_3$,[26] the combined organic phases were washed with five 2 mL portions of H_2O, dried over $MgSO_4$, concentrated under reduced pressure (→ 10 mbar) to deliver the (usually volatile) desired furan (*rac*-)**13**. For the work up, a 20 mL syringe rather than an extraction funnel was utilized.

Acidic work up to extracted $OP(OEt)_3$ (amine 13a,b)

The reaction suspension was cooled in an ice bath, conc. HCl-solution in water (8.0 equiv) was added dropwise and the resulting solution solution was concentrated under reduced pressure (→20 mbar). The residue was diluted with H_2O ([13] = 0.5 mol/L, pH of the mixture ≤ 0) and washed with EtOAc (5x, H_2O/EtOAc 1:1) [27]. The aqueous phase was cooled again to 0 °C, 0.85 g of K_2CO_3 (8.0 equiv) were

25. Except of substrate **12f** a quantitative conversion was achieved by slowly warming to reaction mixture from −78 °C to −40/−30 °C. However, by removing the cooling bath and stirring at ambient temperature as indicated the desired heterocycles **13** were isolated in essentially the same yields and purities, as by allowing the reaction mixture to warm to −40/−30 °C slowly.
26. After saponification crude **13** contained approximately 10−20 mol % of $O=P(OEt)_3$.
27. After washing with three portions of EtOAc, neutralisation with K_2CO_3 and extraction with *n*-Pen approximately 5 mol % of $OP(OEt)_3$ (referred to the product **13a** according to ^{1}H-NMR) remained in crude **13a**. While Et_2O is a too poor washing solvent to remove triethyl phosphate, with CH_2Cl_2 some of the product **13a** is co-extracted.

added carefully (CO_2 evolution), whereby an oil precipitated. Finally, the basic (pH = 10–11) mixture was extracted with *n*-Pen (3x, H_2O/*n*-Pen 1:1), the collected *n*-Pen-phases were dried over $MgSO_4$ and concentrated in vacuo (→10 mbar). The residue was diluted with *n*-Pen and concentrated again under reduced pressure (→10 mbar, repeated one more time) to remove residual NEt_3 yielding amine 13. Analytical data of compounds **13a** and **13d–h** are given in the Supporting Information of [2].

***N*-Benzylpyrrolidine (13b)**

Yield: 219 mg (1.36 mmol, 81%, pale yellow, thin oil) after acidic work up.

***N*-Benzylpiperidine (13b)**

Yield: 284 mg (1.62 mmol, 63%, yellow oil) after acidic work up.

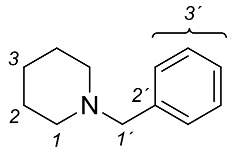

M ($C_{12}H_{17}N$) = 175.270 g/mol; ^{1}H NMR (300 MHz, $CDCl_3$) δ [ppm] = 7.31-7.19 (m, 5H, H-3´), 3.46 (s, 2H, H-2´), 2.37-2.35 (m, 4H, H-1), 1.60-1.53 (m, 4H, H-2), 1.45-1.42 (m, 1H, H-3); ^{13}C NMR (75 MHz, $CDCl_3$) δ [ppm] = 138.6 (C-2´), 129.2/128.0/126.8 (C-3´), 63.9 (C-2´), 54.5 (C-1), 26.0 (C-2), 24.4 (C-3); IR (ATR) ν [cm^{-1}] = 3081 (w), 3060 (w), 3024 (w), 2929 (vs), 2849 (w), 2790 (m), 2751 (m), 2682 (w), 1942 (w), 1872 (w), 1803 (w), 1600 (w), 1584 (w), 1492 (m), 1465 (w), 1452 (s), 1440 (m), 1390 (w), 1367 (m), 1344 (s), 1297 (w), 1269 (m), 1246 (m), 1196 (w), 1152 (s), 1111 (vs), 1065 (w), 1037 (s), 1027 (m), 994 (s), 962 (w), 905 (w), 860 (s), 787 (s), 731 (vs), 695 (vs); GC-MS (EI, 70 eV,) m/z [%] = 175 (60, [M]$^+$), 174 (65, [M-H]$^+$), 146 (5), 132 (5), 98 (30, [M-Ph]$^+$), 91 (100, [Bn]$^+$), 25 (15, [M-Bn]$^+$), 77 (5, [Ph]$^+$) 65 (15, [Cp]$^+$), 55 (10).

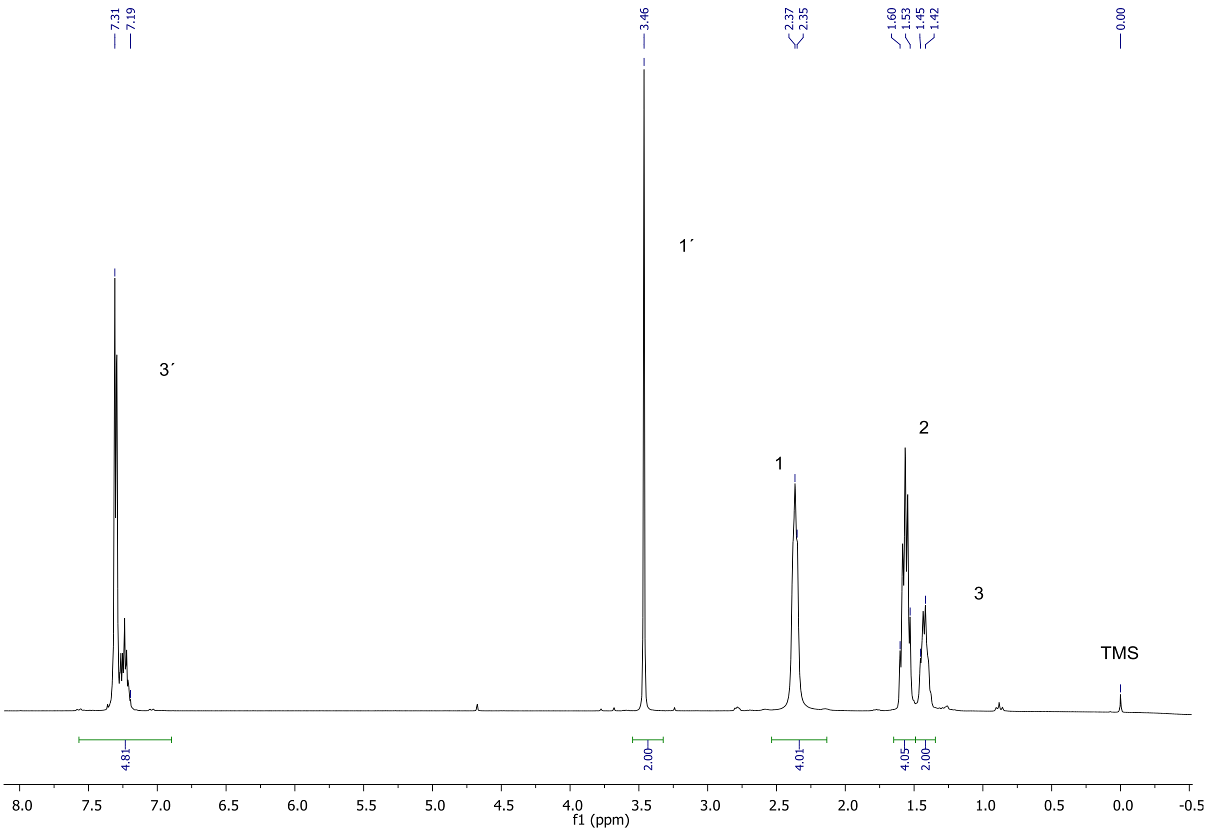

^{1}H NMR-spectra of piperidine **13b** in $CDCl_3$ (400 MHz).

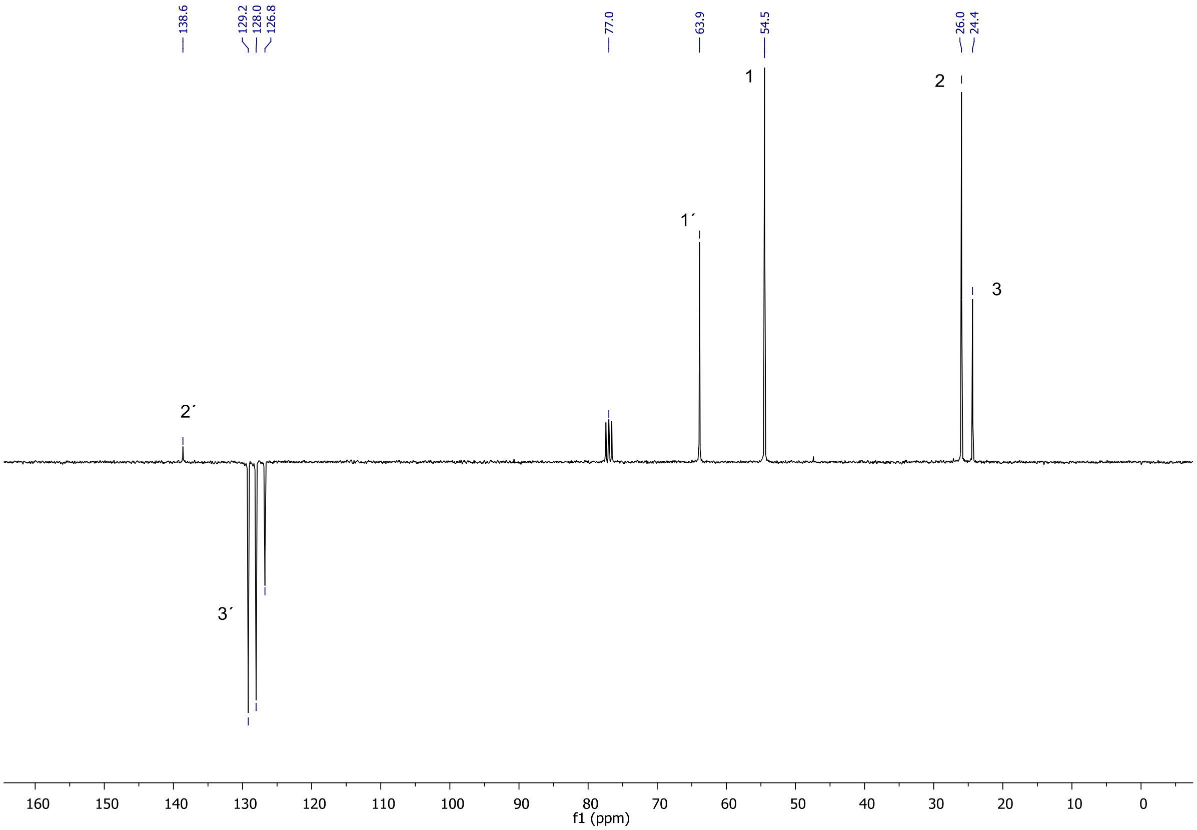

^{13}C NMR-spectra of piperidine **13b** in $CDCl_3$ (100 MHz).

***rac*-2-Phenyltetrahydrofuran (*rac*-13d)**

Yield: 138.6 mg (0.935, 92%, pale yellow oil)

***rac*-2-(2-Chlorophenyl)tetrahydrofuran (*rac*-13e)**

Yield: 189.5 mg (1.04 mmol, 85%, pale yellow oil).

***rac*-2-(4-Bromophenyl)tetrahydrofuran (*rac*-13f)**

Yield: 206.8 mg (0.91 mmol, 83%, pale yellow oil).

***rac*-2-(2,4,6-Trimethylphenyl)tetrahydrofuran(*rac*-13g)**

Yield: 316 mg (1.66 mmol, 84%, yellow oil).

2,2-Diphenyltetrahydrofuran (13h)

Yield: 211.7 mg (0.94 mmol, 82%, colorless solid)

3.8 Initial synthesis of *trans*-piperidinol 11a (Scheme 6)

3.8.1 Synthesis of (*S*)-2-(benzylamino)-6-hydroxy-3-hexanone (14a)

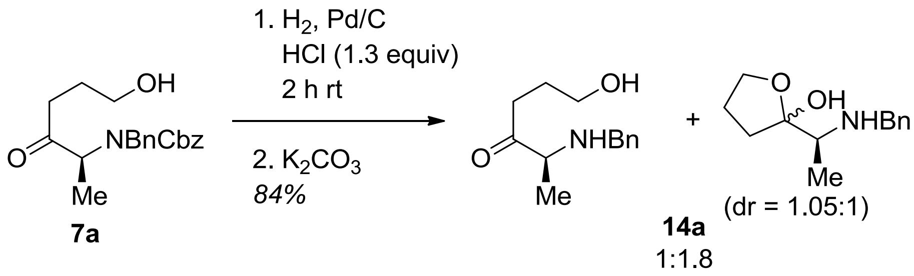

The ketone **7a** (1.682 g, 4.73 mmol, 1.0 equiv) was dissolved in MeOH (10 mL, [**7a**] = 0.5 mol/L) and cooled in an ice bath. Then 32% HCl solution in water (0.60 mL, 6.15 mmol, 1.3 equiv) and Pd/C (54 mg, 10 wt %) were added. Subsequently, the cooling bath was removed and the suspension was stirred under 1 atm of H_2 atmosphere (balloon) for 2 h.

In the following the reaction mixture was filtered through a thin layer of celite, the celite layer was purged with MeOH (2 x 5 mL) and the collected filtrates were concentrated under reduced pressure. The residue was dissolved in 1 N HCl solution (aq., 6 mL) and Et_2O (4 mL), the aqueous phase was washed with further Et_2O (2 x 4 mL), cooled in an ice bath and treated with an excess of K_2CO_3 (1.40 g, CO_2 evolution), whereby a pale yellow oil precipitated. The mixture (pH = 9) was diluted with water (2 mL) in order dissolve precipitated KCl, extracted with CH_2Cl_2 (3 x 6 mL), the combined CH_2Cl_2 phases were dried over $MgSO_4$ and concentrated under reduced pressure. After drying in high vacuum for 1 h the amine **14a** was obtained as a pale yellow oil (945 mg). Considering 20 mol % of residual CH_2Cl_2 according to ^{1}H NMR the ketone **14a** was isolated in a yield of 84% (3.97 mmol).

M ($C_{13}H_{19}NO_2$) = 221.296 g/mol; **r_f** (SiO_2, EtOAc/*n*-Hex) = 0.21 (1:1, tailing); **^{1}H NMR** (400 MHz, $CDCl_3$) δ [ppm] = 7.33-7.23 (m, 15H, H-3´, H-3´), 4.08-3.60 (m, 9H, H-6, H-1´, H-6, H-1´), 3.41 (q, 1H, H-2, *J* = 7.3 Hz, **2a**/**2b** 1:1.8)), 2.85 (q, 1H, H-2, diastereomer 1, *J* = 6.7 Hz, *dr* = 1:1.05), 2.79 (q, 1H, H-2, diastereomer 2, *J* = 6.4 Hz), 2.71-2.46 (m, 2H, H-4), 2.17-1.60 (m, 10H, H-5, H-4, H-5), 1.25 (d, 1H, H-1, *J* = 6.5 Hz, **2a**/**2b** 1:1.8 (*dr* = 1:1.05)), 1.16 (d, 1H, H-1, diastereomer 1, *J* = 6.2 Hz, *dr* = 1:1.05), 1.08 (d, 1H, H-1, diastereomer 2, *J* = 6.5 Hz); **^{13}C NMR** (100 MHz, $CDCl_3$) δ [ppm] = 214.0 (C-3), 140.4/3x140.0 (C-2´, C-2´), 128.5/2x128.4/128.2/128.1/128.0/127.1/127.0/ 126.9 (C-3´, C-3´), 107.0/106.1 (C-3´), 67.7/67.2 (C-6´), 61.9 (C-6´), 61.8 (C-2), 58.7/58.4 (C-2´), 51.9/51.6/51.4 (C-1´, C-1´), 36.1 (C-4), 34.2/33.3 (C-4), 26.4 (C-5), 25.0/23.9 (C-5), 18.1 (C-1), 16.5/13.9 (C-1´); **IR** (film) ν [cm^{-1}] = 3388 (br), 3323 (br), 3086 (w), 3063 (w), 3029 (w), 2967 (m), 2932 (s), 2875 (s), 1955 (w), 1881 (w), 1814 (w), 1770 (w), 1713 (vs), 1603 (w), 1586 (w), 1495 (w), 1454 (s), 1408 (w), 1372 (m), 1322 (w), 1238 (w), 1198 (w), 1106 (m), 1061 (s), 1028 (s), 913 (w), 847 (w), 745 (s), 699 (s), 599 (w), 489 (w); **HR-MS** (ESI, $[C_{13}H_{20}NO_3]^+$) calc. 222.1494 u, found 222.1495 u; **$[\alpha]_D$** from two different

batches (c = 1.216 g/100 mL, $CHCl_3$, T = 22.0 °C) = +14.5, (c = 0.554 g/100 mL, $CHCl_3$, T = 21.5 °C) = +16.3 °C.

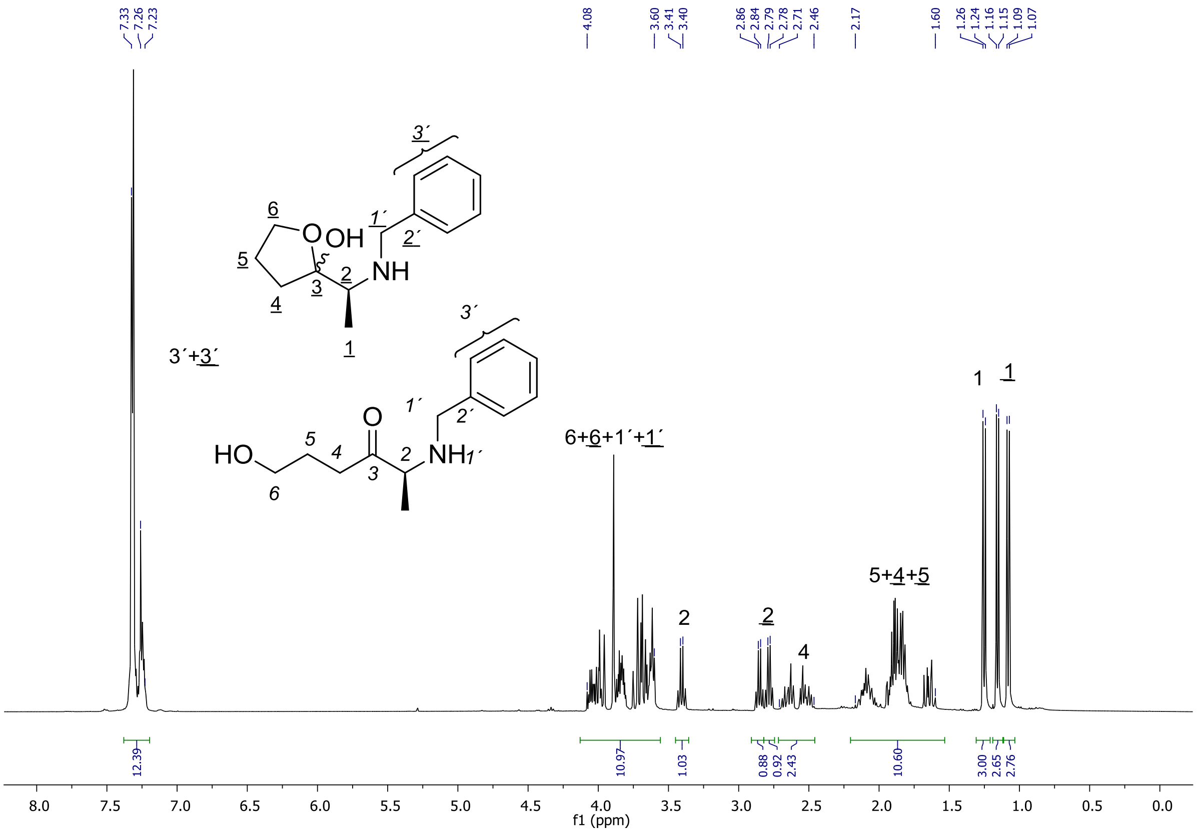

^{1}H NMR-spectra of the furane/ketone **14a** in $CDCl_3$ (400 MHz).

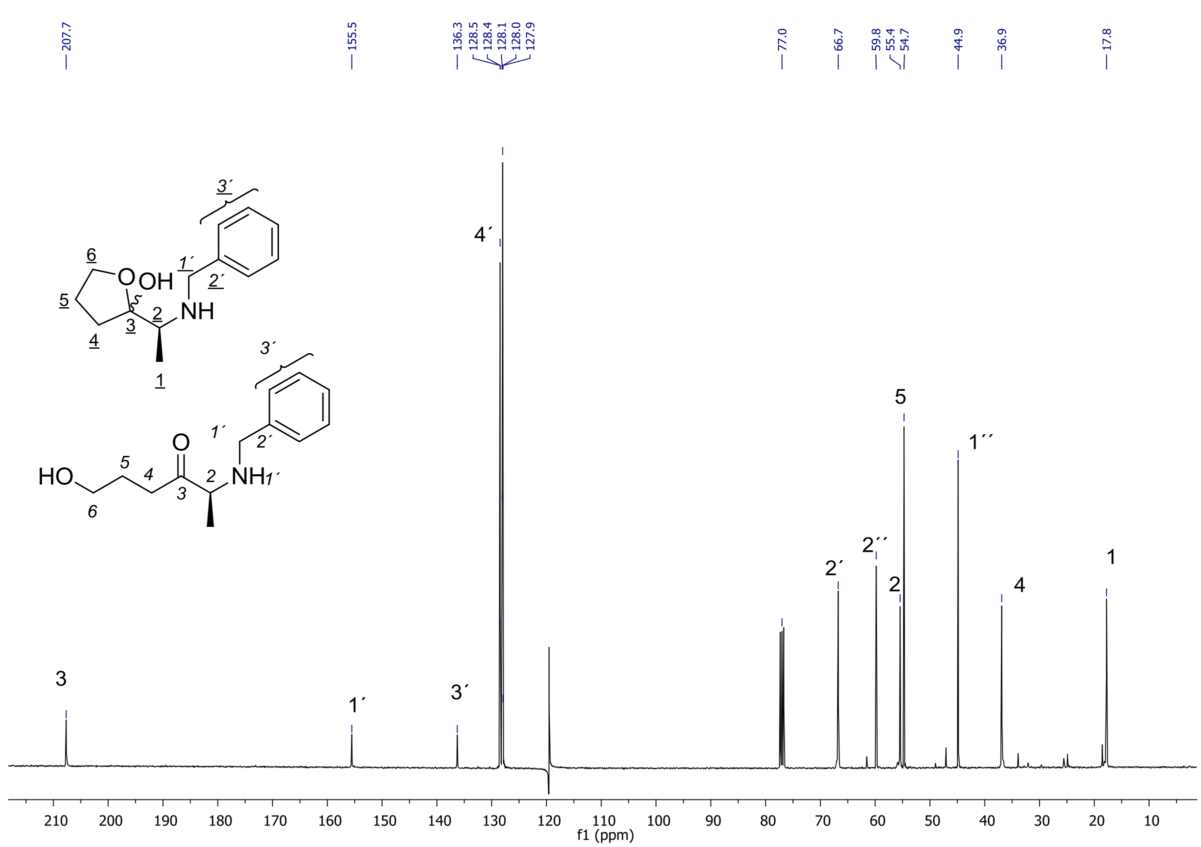

^{13}C NMR-spectra of the furane/ketone **14a** in $CDCl_3$ (400 MHz).

3.8.2 Synthesis of (2*S*,3*R*)-2-(benzylamino)-3,6-hexandiol (anti-9a)

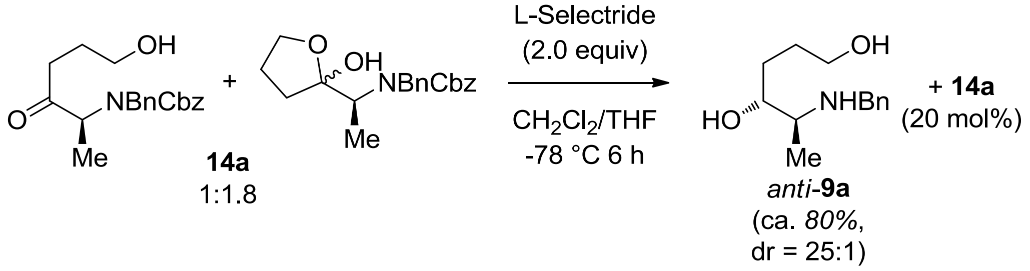

Under an atmosphere of argon the ketone **14a** (844 mg, 3.67 mmol, 10 mol % residual CH_2Cl_2, 1.0 equiv) was dissolved in dry CH_2Cl_2 (4 mL, [**14a**] = 0.5 mol/L), cooled to –78 °C and 1.0 N L-Selectride solution in THF (7.3 mL, 7.34 mmol, 2.0 equiv) was added dropwise within 15 min. After 6 h of stirring at –78 °C, glacial acetic acid (0.42 mL, 7.34 mmol, 2.0 equiv) and after 5 min of stirring 1 N HCl solution in water (8 mL) were added dropwise. Then the cooling bath was removed and the mixture was allowed to warm to room temperature. Subsequently, CH_2Cl_2 and THF were evaporated under reduced pressure, the residue (emulsion) was washed with Et_2O (2 x 4 mL), cooled in an ice bath and treated carefully portion-wise with K_2CO_3 (1.4 g, 10.0 mmol, CO_2-evolution). Precipitated KCl was dissolved with H_2O (2 mL), the mixture (**9a** precipitated as an oil) was extracted with CH_2Cl_2 (3 x 6 mL), the collected CH_2Cl_2-phases were dried over $MgSO_4$ and dried in high vacuum for 0.5 h. The crude

amine **14a** (886 mg, colorless oil) was converted further without additional purification. According to ^{1}H NMR **14a** was obtained with in a dr of 25:1 *anti/syn* and in 80% conversion.

M ($C_{13}H_{12}NO_2$) = 223.311 g/mol; **r$_f$** (SiO_2) = 0.19 (MeOH/EtOAc 1:5); **^{1}H NMR** (400 MHz, $CDCl_3$, dr = 25:1 *anti/syn*) δ [ppm] = 7.35-7.24 (m, 5H, H-3´), 3.83-3.62 (m, 5H, H-2, H-3, H-6, H-1´), 2.78-2.73 (m, 1H, H-2) 2.44 (s (broad), 3H, 2xOH, NH), 1.77-1.70 (m, 2H, H-4) 1.55-1.39 (m, 2H, H-5), 1.13 (d, 3H, H-1´´$_{syn}$, *J* = 6.4 Hz, dr = 1:25 *syn/anti*), 1.02 (d, 3H, H-1´´$_{anti}$, *J* = 6.6 Hz, dr = 25:1 *anti/syn*); **^{13}C NMR** (100 MHz, $CDCl_3$) δ [ppm] = 140.1 (C-2´), 128.5/128.0/127.1 (C-3´), 71.0 (C-3), 62.9 (C-6), 56.5 (C-2), 51.3 (C-1´), 30.4/29.8 (C-4, C-5), 14.3 (C-1´´); **IR** (film) ν [cm^{-1}] = 3402 (br), 3090 (w), 3064 (w), 3029 (w), 2941 (m), 2871 (w), 1644 (m), 1497 (w), 1453 (m), 1379 (w), 1059 (w), 1029 (w), 913 (s), 699 (s); **[α]$_D$** (c = 0.198 g/100 mL, $CHCl_3$, T = 22.0 °C) = +4.1.

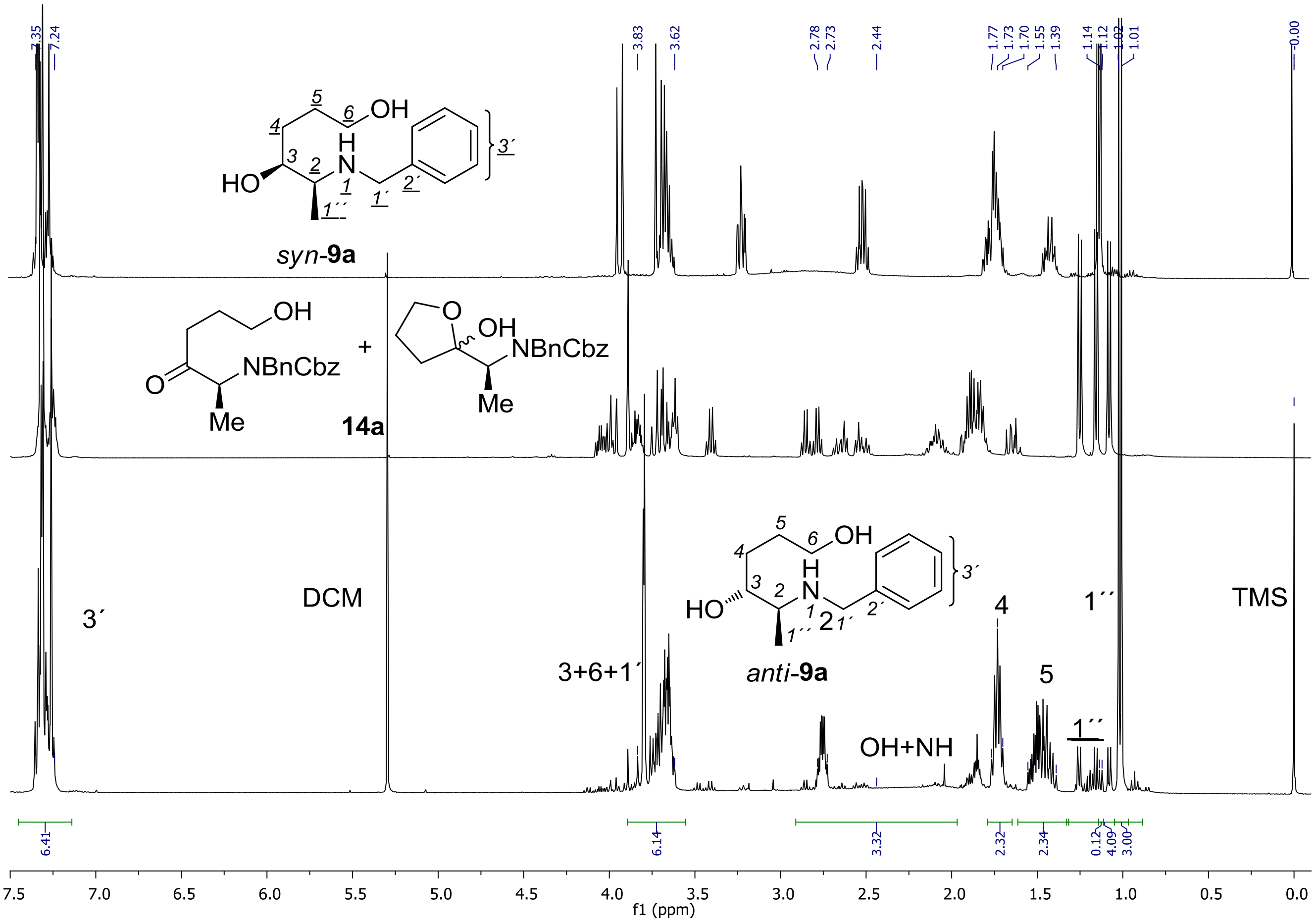

^{1}H NMR-spectra of the (minor) *syn*-amino alcohol *syn*-**9a**, the starting material **14a** and the reaction product *anti*-**9a** (dr = 25:1 *anti/syn*, order top to bottom, 400 MHz).

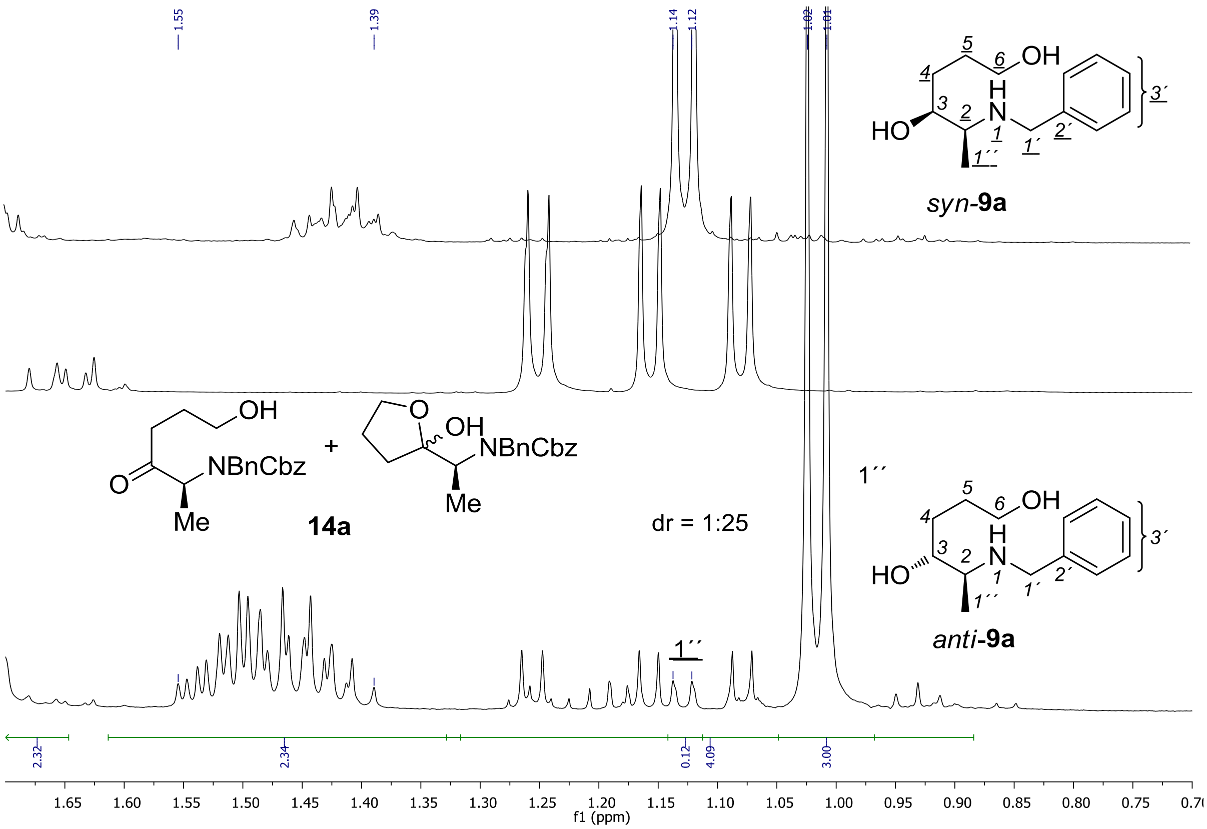

Enlargement of ^{1}H NMR-spectra of the (minor) *syn*-amino alcohol *syn*-**9a**, the starting material **14a** and the reaction product *anti*-**9a** showing the Me-signals (dr = 25:1 *anti/syn*, order top to bottom, 400 MHz).

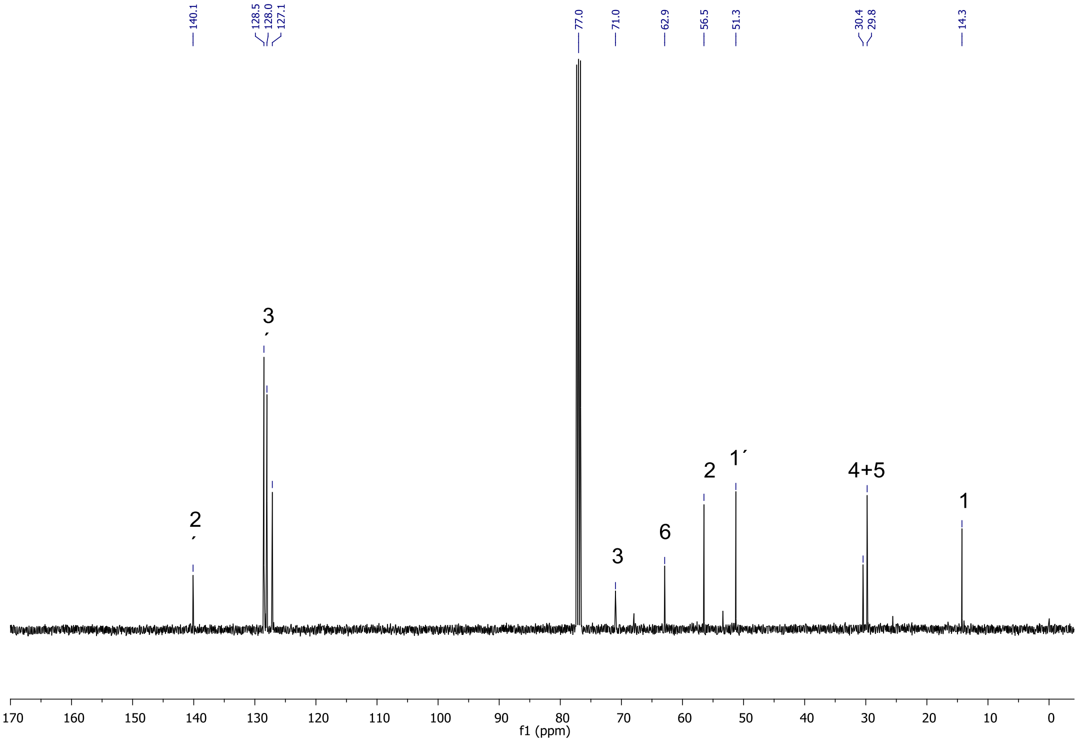

^{13}C NMR-spectra of the amino alcohol *anti*-**9a** in $CDCl_3$ (100 MHz).

3.8.3 Synthesis of (2*S*,3*R*)-1-benzyl-2-methyl-3-piperidinol (*trans*-11a)

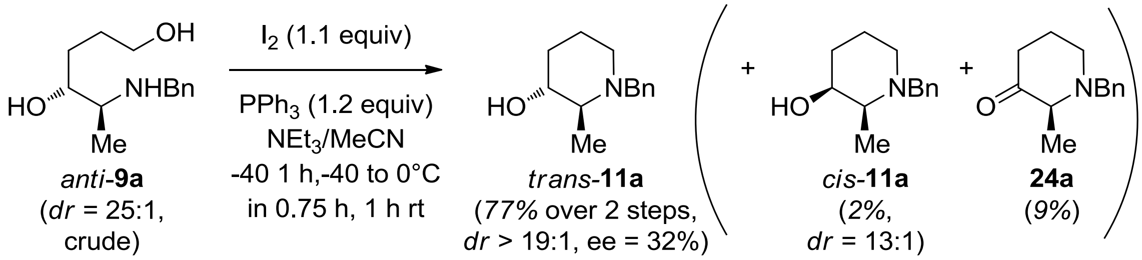

The piperidinol *trans*-**11a** was prepared according to the general procedure for the synthesis of Piperidinols *cis*-**11a–c** via Phosphane mediated Cyclodehydration (see chapter 3.6.2). Early (diluted) fractions during chromatographic preparation contained the *cis*-configured piperidine *cis*-**11a** (18 mg, 0.09 mmol, 2%, dr = 13:1). The major product *trans*-**11a** (700 mg, → 2.81 mmol, 77% (from **14a**) accounting 30 mol % of residual *c*-Hex, dr > 19:1, ee = 32%) was isolated as a pale yellow oil. Aside the ketone **24a** was isolated as a red oil in 9% yield resulting from cyclodehydration of the amino ketone **14a** due to incomplete conversion in the foregoing reduction step (R_f = 0.88 (iPrOH/*n*-Hex 1:5)). **$[\alpha]_D$** (*c* = 1.188 g/100 mL, $CHCl_3$, 23.0 °C) = +12.3. Remaining analytical data was in agreement with the piperidinol *trans*-**11a** prepared as described in Scheme 7.

HPLC-chromatograms of the racemic and enantioenriched piperidine *trans*-**11a** (*dr* > 19:1 *cis/trans*; separation conditions: column Chiralpak AI from Daicel Industries, flow 1 mL/min, eluent EtOH/*n*-Hex/NEt_3 1:100:0.1, detected wavelength 254 nm):

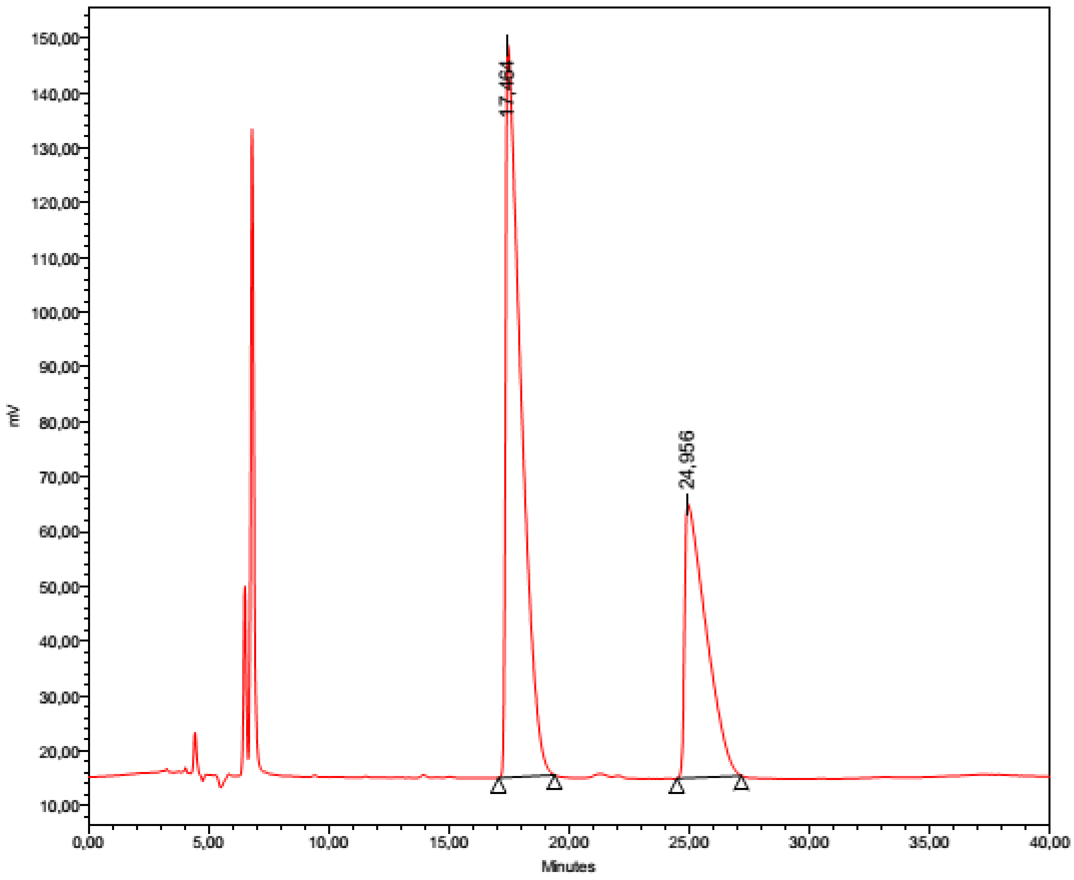

Retention Time (min)	Area	% Area	Height
17,464	5951097	65,76	133556
24,956	3098105	34,24	49867

3.9 Improved synthesis of *trans*-piperidinol 11a (Scheme 7)

Experimental procedures and analytical data of compounds **15a** and *trans*-**11a** (including HPLC chromatograms of derivative *trans*-**11a**) are present in the Supporting Information of [2].

3.10 Synthesis of *L-733,060* (Scheme 8)

Experimental procedures and analytical data including HPLC chromatograms of derivative *cis*-**16c** are given in the Supporting Information of [2].
